# Identification of a neutrophil-specific PIK3R1 mutation facilitates targeted treatment in a patient with Sweet syndrome

**DOI:** 10.1172/JCI162137

**Published:** 2023-01-03

**Authors:** Shreya Bhattacharya, Sayon Basu, Emily Sheng, Christina Murphy, Jenny Wei, Anna E. Kersh, Caroline A. Nelson, Joshua S. Bryer, Hovik A. Ashchyan, Katherine Steele, Amy Forrestel, John T. Seykora, Robert G. Micheletti, William D. James, Misha Rosenbach, Thomas H. Leung

**Affiliations:** 1 Dermatology Department, Perelman School of Medicine, University of Pennsylvania; Philadelphia, Pennsylvania, USA.; 2 Corporal Michael J. Crescenz Veterans Affairs Medical Center, Philadelphia, Pennsylvania, USA.

**Keywords:** Dermatology, Inflammation, Neutrophils, Signal transduction, Skin

## Abstract

**Background:**

Acute febrile neutrophilic dermatosis (Sweet syndrome) is a potentially fatal multiorgan inflammatory disease characterized by fever, leukocytosis, and a rash with a neutrophilic infiltrate. The disease pathophysiology remains elusive, and current dogma suggests that Sweet syndrome is a process of reactivity to an unknown antigen. Corticosteroids and steroid-sparing agents remain frontline therapies, but refractory cases pose a clinical challenge.

**Methods:**

A 51-year-old woman with multiorgan Sweet syndrome developed serious corticosteroid-related side effects and was refractory to steroid-sparing agents. Blood counts, liver enzymes, and skin histopathology supported the diagnosis. Whole-genome sequencing, transcriptomic profiling, and cellular assays of the patient’s skin and neutrophils were performed.

**Results:**

We identified elevated IL-1 signaling in lesional Sweet syndrome skin caused by a PIK3R1 gain-of-function mutation specifically found in neutrophils. This mutation increased neutrophil migration toward IL-1β and neutrophil respiratory burst. Targeted treatment of the patient with an IL-1 receptor 1 antagonist resulted in a dramatic therapeutic response and enabled a tapering off of corticosteroids.

**Conclusion:**

Dysregulated PI3K/AKT signaling is the first signaling pathway linked to Sweet syndrome and suggests that this syndrome may be caused by acquired mutations that modulate neutrophil function. Moreover, integration of molecular data across multiple levels identified a distinct subtype within a heterogeneous disease that resulted in a rational and successful clinical intervention. Future patients will benefit from efforts to identify potential mutations. The ability to directly interrogate the diseased skin allows this method to be generalizable to other inflammatory diseases and demonstrates a potential personalized medicine approach for patients with clinically challenging disease.

**Funding Sources:**

Berstein Foundation, NIH, Veterans Affairs (VA) Administration, Moseley Foundation, and H.T. Leung Foundation.

## Graphical abstract

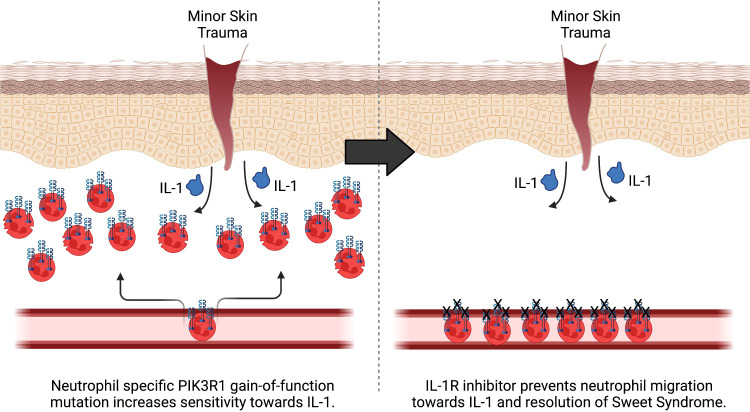

## Introduction

Individuals with Sweet syndrome (acute febrile neutrophilic dermatosis) present with fever, leukocytosis, and an eruption of erythematous plaques with a dense dermal neutrophilic infiltrate on histology (1, 2). Systemic multiorgan involvement may occur and carries a considerable mortality risk. Sweet syndrome is often categorized into classic (associated with infection, inflammatory disorders, vaccination, or pregnancy), malignancy-associated, and drug-induced subtypes (1–3). The molecular pathogenesis of Sweet syndrome remains unknown, but current dogma suggests a cell-extrinsic mechanism of neutrophil hypersensitivity to an unknown trigger leading to cytokine dysregulation during the inflammatory response (4). Systemic corticosteroids are the first-line therapy for Sweet syndrome, and their chronic use results in well-characterized morbidity. Steroid-sparing antiinflammatory agents such as colchicine, dapsone, and potassium iodide may be used for long-term management, but patients refractory to these agents pose a clinical challenge (4).

Here, we describe a patient with Sweet syndrome with systemic disease, who required chronic use of systemic corticosteroids and was hospitalized 4 times over 2 years for disease flares. Detailed genomic and transcriptomic studies identified the underlying genetic mutation and molecular mechanism responsible for her disease. We started therapy with a targeted treatment using the IL-1 receptor (IL-1R) inhibitor anakinra, which resulted in complete disease remission and enabled a tapering off of all systemic corticosteroids.

## Results

### Clinical presentation.

**Figure 1:**
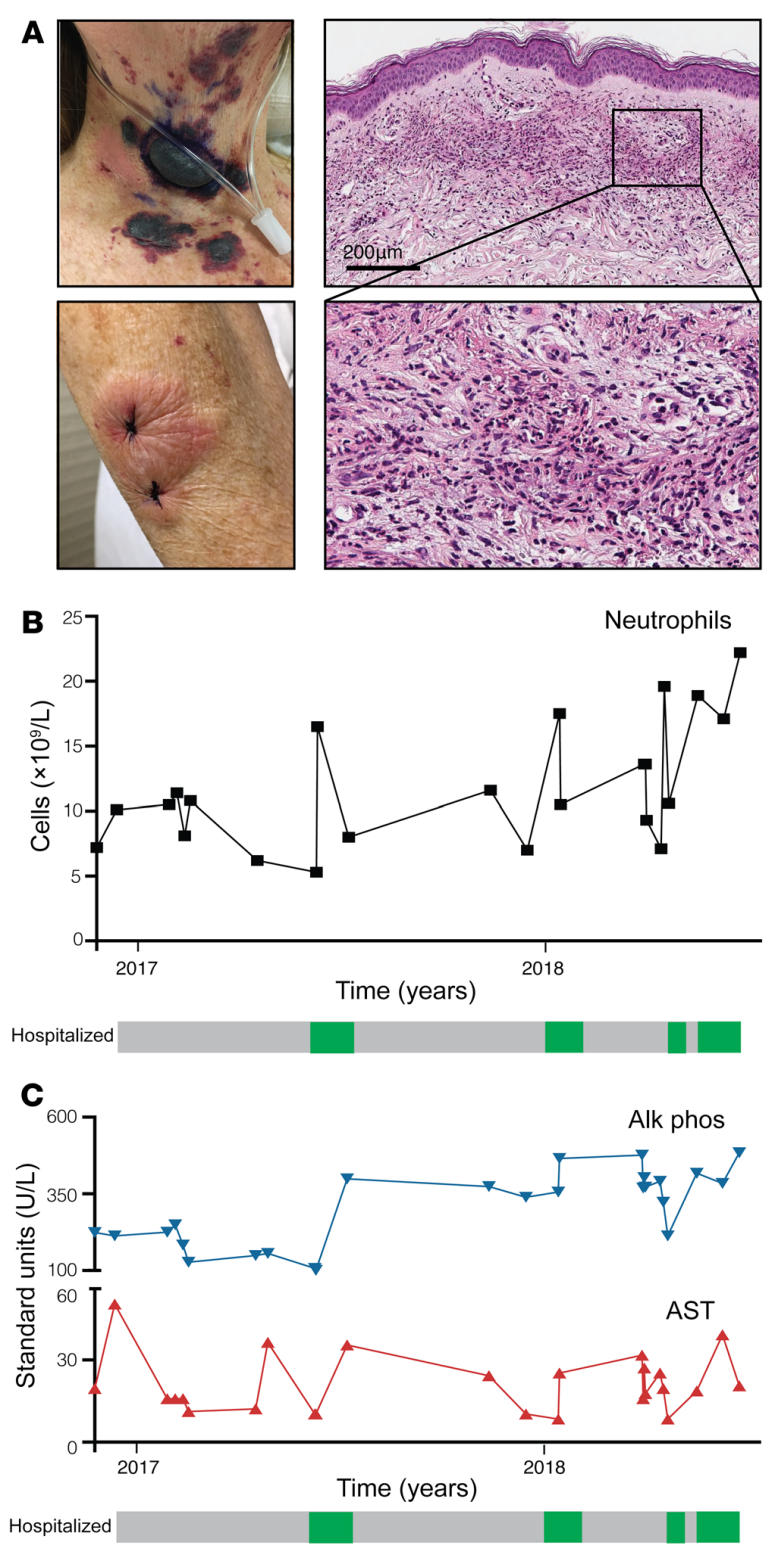
The patient with refractory Sweet syndrome required multiple hospitalizations to control the disease. (**A**) Clinical photographs of the patient’s skin lesions. Panels on the right are H&E-stained images of the patient’s skin biopsy, which demonstrate a diffuse dermal neutrophilic infiltration. Scale bar: 200 μM. Original magnification, ×20 (enlarged inset). (**B** and **C**) Time course of the patient’s peripheral neutrophilia (**B**) and liver enzyme fluctuations (**C**) during her multiple clinical flares. The patient’s 4 hospitalizations for disease flares are denoted by the green boxes. Alk phos, alkaline phosphatase; AST, aspartate aminotransferase.

A 51-year-old White woman with a past medical history of stable pulmonary hyalinizing granuloma was referred to dermatology for diffuse red plaques (Figure 1A and Supplemental Figure 1A; supplemental material available online with this article; https://doi.org/10.1172/JCI162137DS1). She reported joint pain and waxing and waning skin lesions over the preceding 6 months.

Skin biopsies repeatedly revealed typical dermal neutrophilic inflammation consistent with Sweet syndrome; histiocytoid morphology was not observed (Figure 1A and Supplemental Figure 1A).

The patient developed additional symptoms of fevers, neutrophilia, sterile pyuria, and right upper-quadrant abdominal pain with her disease flares, all of which responded to systemic steroid therapy (Figure 1, B and C). We conducted a thorough evaluation to rule out other causes of her presentation and to evaluate for underlying conditions associated with Sweet syndrome. Repeated elevations in transaminases and alkaline phosphatase during disease flares led to a referral to hepatology, and liver imaging demonstrated features of primary sclerosing cholangitis, but serologic workup and rheumatologic evaluation were negative for autoimmunity and infection, including negative antineutrophilic cytoplasmic antibodies and negative tissue cultures. Liver biopsy demonstrated periportal fibrosis and mild bile ductular proliferation with predominantly neutrophilic inflammation. Examination of the bone marrow revealed a normal karyotype, cytogenetics, and morphology. Review of her prior pulmonary hyalinizing granuloma pathology and biopsy of her sinuses ruled out IgG4-mediated disease. Taken together, we diagnosed the patient with multiorgan Sweet syndrome with cutaneous and hepatic involvement.

For 5 years, the patient was unable to taper off of prednisone without suffering a flare of her symptoms. Multiple steroid-sparing agents were trialed, including dapsone, potassium iodide, colchicine, hydroxychloroquine, sulfapyridine, and methotrexate. The patient developed significant steroid-related side effects including ecchymoses, weight gain, severe osteopenia, and spontaneous vertebral fractures. She continued to experience clinical flares and presented to the emergency department frequently, resulting in 4 hospital admissions within 2 years. During these flares, her complete blood count consistently showed leukocytosis and neutrophilia, and her transaminases and alkaline phosphatase were markedly elevated (Figure 1, B and C). Repeat skin biopsies remained consistent with Sweet syndrome (Supplemental Figure 1A).

Given the failure to identify an effective and safe long-term therapy and the patient’s significant morbidity from corticosteroids, we hypothesized that a molecular characterization of her skin may enable the identification of altered pathways that could be readily targeted by currently available FDA-approved therapeutic agents. Targeted augmentation of immunological pathways has become a powerful approach to treat human inflammatory diseases. In addition to this personalized treatment approach, understanding the transcriptomic changes in this patient’s disease could expand our insight into Sweet syndrome pathophysiology.

### Transcriptomic analysis of lesional skin reveals IL-1β–dominant inflammation.

**Figure 2:**
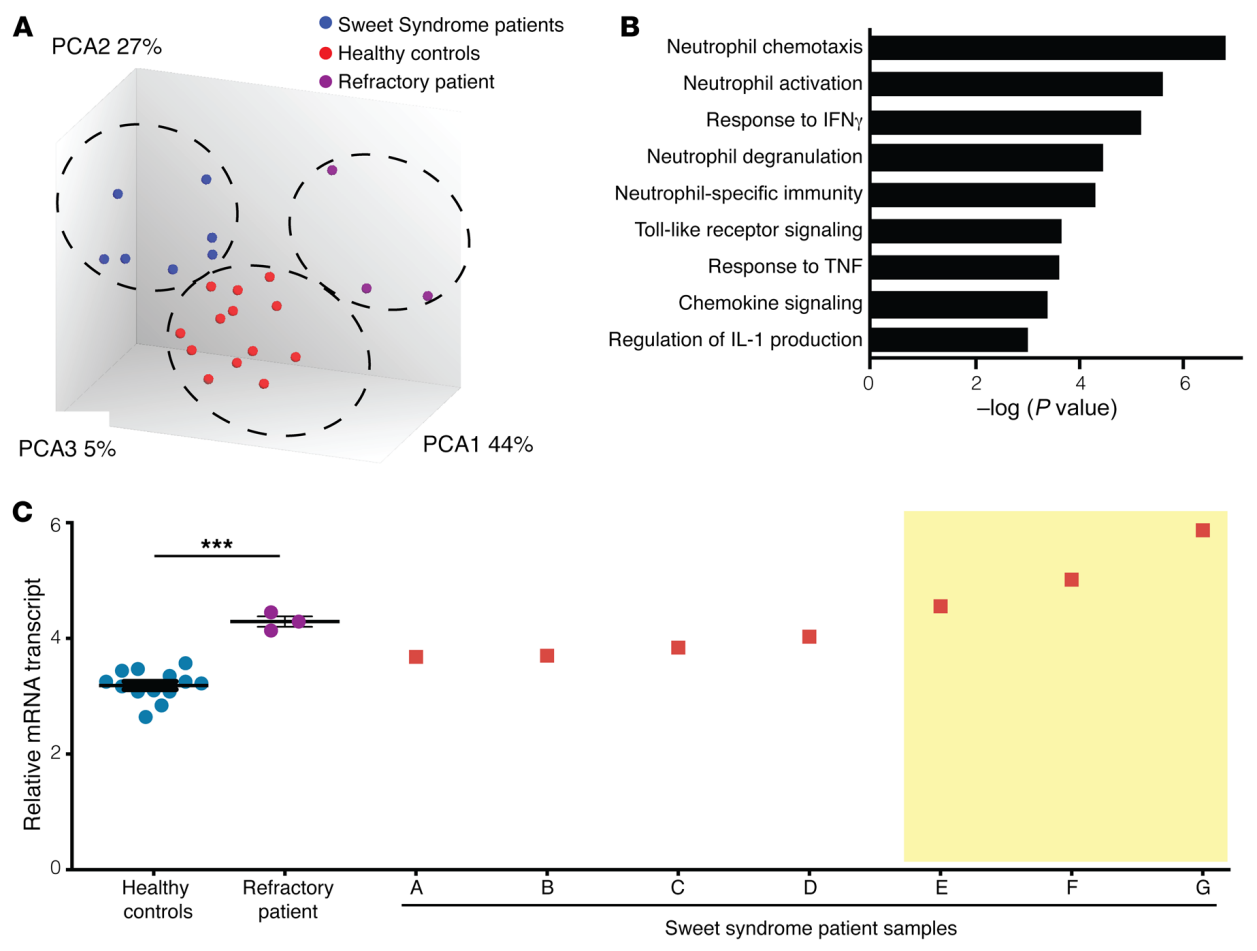
Refractory Sweet syndrome lesions reveal IL-1β–dominant inflammation. (**A**) PCA of gene expression generated from skin dermis from the patient with refractory Sweet syndrome (*n* = 3, purple), other patients with Sweet syndrome (*n* = 7, blue), and healthy controls (*n* = 13, red). (**B**) Gene ontology categories that were most highly enriched in the transcriptome of the refractory patient with refractory disease compared with the healthy controls. (**C**) Increased *IL1B* transcript levels were detected in the refractory patient’s dermis (purple) compared with levels in healthy control dermis (blue). Individual dermis samples from patients with Sweet syndrome exhibited varied levels of *IL1B* transcripts. Three patients had transcript levels greater than 2.5 SDs above the average for the healthy controls (yellow box). Data represent the mean ± SEM. ***P < 0.001, by 2-tailed Student’s *t* test.

To determine what genes were differentially expressed in the patient’s Sweet syndrome lesions, we performed microarray profiling on dermal RNA extracted from paraffin-embedded skin biopsy samples obtained from the patient (*n* = 3, taken from 3 consecutive years), other patients with Sweet syndrome (*n* = *7*), and healthy controls (*n* = 13). Principal component analysis (PCA) demonstrated a separation between the 3 groups, indicating that the patient’s disease was different from that of the other patients with Sweet syndrome whose samples were studied (Figure 2A). We identified 1,622 differentially expressed genes in the patient’s skin lesions compared with healthy control skin (FDR <0.1; fold change [FC], >1.5 or <–1.5; Supplemental Table 1). Gene ontogeny pathways and transcriptome analysis revealed enrichment for genes involving neutrophil-specific functions as well as genes involving neutrophil-specific functions and regulation of IL-1 production (Figure 2B). Indeed, *IL1B* gene expression was substantially increased in the refractory patient’s microarray samples (Figure 2C). We confirmed this result by quantitative PCR (qPCR) of additional dermal RNA samples from the patient and healthy controls (Supplemental Figure 1B). Among the seven other patients with Sweet syndrome, 3 exhibited a similar increase in *IL1B* transcript levels in their microarray compared with those of healthy controls (Figure 2C, yellow box). Finally, transcript levels of neutrophil marker genes (including myeloperoxidases, matrix metallopeptidases, arginases, and defensins) exhibited no significant increase in the patient with refractory disease or in the other patients with Sweet syndrome compared with the healthy controls, suggesting that the increase in IL-1β was not simply due to more neutrophil recruitment (Supplemental Table 1). Taken together, these data suggested a role for IL-1β production in a subset of patients with Sweet syndrome, including our index patient.

### PIK3R1 gain-of-function mutation in neutrophils promotes cell migration toward IL-1β.

**Figure 3:**
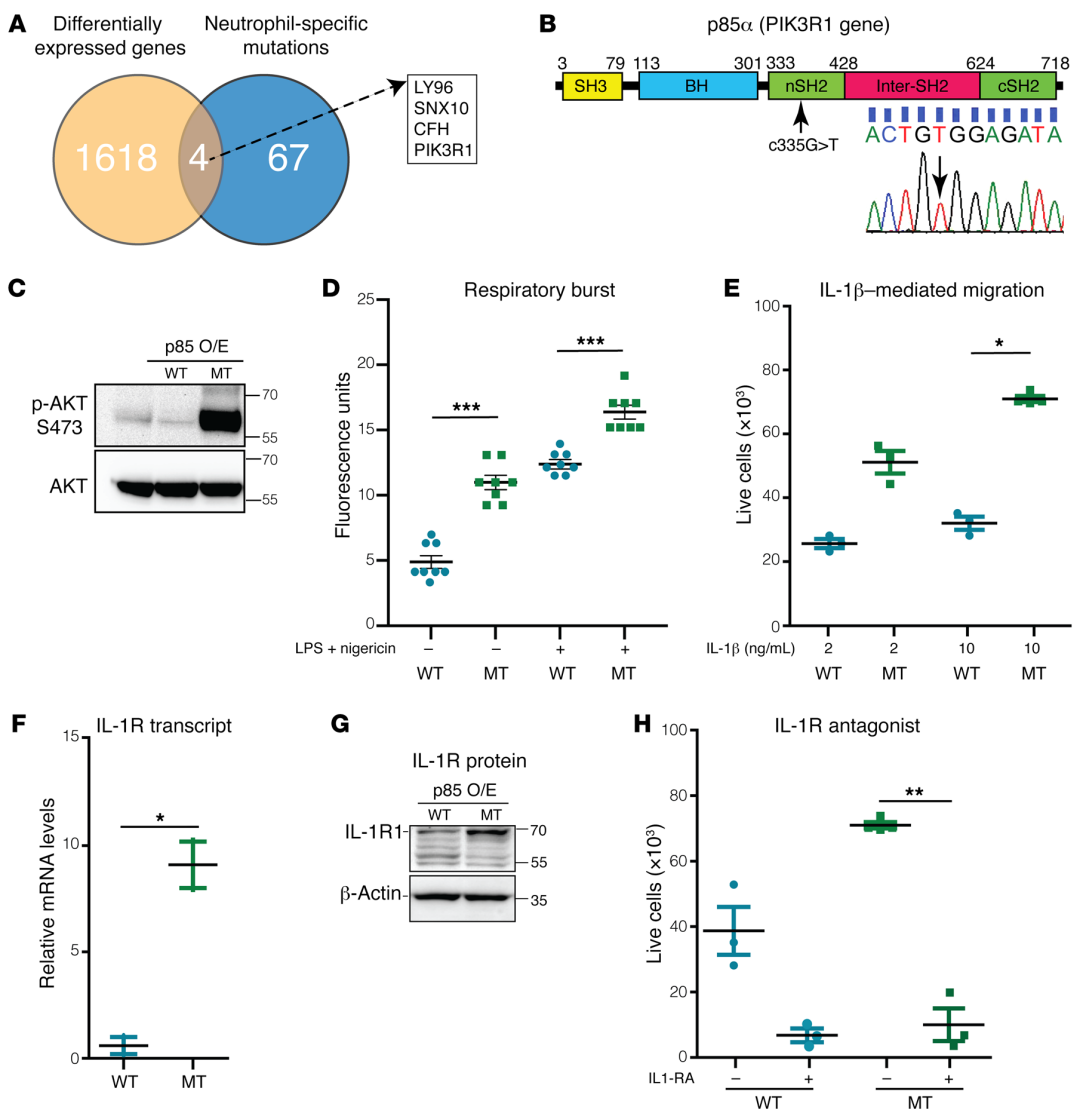
PIK3R1 gain-of-function mutation increases neutrophil migration toward IL-1β. (**A**) Venn diagram illustrates the intersection between differentially expressed genes and neutrophil-specific gene mutations in the patient with refractory disease. Gene names are listed in the box. (**B**) Schematic depicting the *PIK3R1* gene. The mutation location is identified by the black arrow. Below the schematic is the DNA sequence chromatogram that confirms the mutation. (**C**) Western blot demonstrates increased phosphorylation of AKT at S473 in overexpressed p.W335C p85 (MT) cells compared with overexpressed p85 (WT) cells. The experiment was repeated independently 3 times. (**D**) Cellular respiratory burst detected by DCFDA in LPS-treated WT and MT p85 cells (*n* = 8/group). (**E**) Transwell migration of WT and MT p85 cells in response to IL-1β (*n* = 6/group). (**F**) qPCR (*n* = 3/group) and (**G**) Western blot show increased IL-1R1 expression in MT p85 cells compared with WT p85 cells. The experiment was repeated independently 3 times. (**H**) Pharmacologic treatment with an IL-1RA blocked IL-1β–mediated Transwell migration (*n* = 3/group). Data represent the mean ± SEM. *P < 0.05 and **P < 0.01, and ***P < 0.001, by 2-tailed Student’s *t* test.

We next performed whole-genome sequencing of the patient’s skin-infiltrating neutrophils collected by laser-capture microscopy (LCM) to explore whether 1 or more somatic mutations could be contributing to their disease. Nonlesional skin epidermis was used to establish a reference genome and to rule out naturally occurring SNPs. Seventy-one missense and nonsense neutrophil-specific mutations were identified (Supplemental Table 2). Four of the mutated genes were among the differentially expressed genes identified in the patient (Figure 3A). Of these, a mutation in the phosphoinositide-3-kinase–regulatory subunit 1 (*PIK3R1*) gene was of particular interest, because PI3K signaling was among the differentially regulated pathways identified in our prior transcriptomic analysis. Sanger sequencing of peripheral blood neutrophils confirmed the missense mutation NC_000005.9:g.67588175G>T, which resulted in the substitution of tryptophan for cysteine in exon 8 (p.W335C) of the *PIK3R1* gene (Figure 3B).

PIK3R1 encodes the p85 regulatory protein of the PI3K/AKT signaling pathway, which is important for cellular proliferation, apoptosis, and migration (5, 6). The p.W335C mutation is located within the nSH2 domain, which mediates p85 binding to tyrosine kinase receptors and allows the p110 catalytic binding partner to phosphorylate and activate AKT (Figure 3B) (5, 6). To elucidate the function of this mutation, we used an established in vitro cell culturing system, in which human HL-60 promyeloblast cells exposed to DMSO differentiate into a neutrophil-like state (7). We generated stable HL-60 cell lines that overexpressed WT and mutant p.W335C (Mut) p85 proteins by lentiviral transduction. Flow cytometric analysis confirmed the appropriate differentiation of these cell lines (Supplemental Figure 2 and Supplemental Figure 3A). Differentiated cells were used for all subsequent experiments.

mTOR is a well-established target of AKT, and mTOR acts through 2 functionally distinct complexes, mTOR complex 1 (mTORC1) and -2 (mTORC2) (5, 6). mTORC1 is the rapamycin-sensitive protein complex that regulates protein synthesis, and we observed no difference in mTORC1 activation between WT and Mut p85 cells as assessed by phosphorylation of the classical downstream target S6 (Supplemental Figure 3B). mTORC2 is the rapamycin-insensitive protein complex that regulates cell proliferation, survival, and migration. mTORC2 phosphorylates AKT specifically at S473, and Mut p85 cells exhibited increased phosphorylated AKT (p-AKT) at S473 compared with WT p85 cells (Figure 3C). We observed no differences between WT and Mut p85 cells in cell proliferation or survival (Supplemental Figure 3, C and D). However, WT and Mut p85 cells demonstrated notable differences in neutrophil respiratory burst and cellular migration.

We assessed neutrophil respiratory activity using the 2,7-dichlorofluorescein diacetate (DCFDA) dye in LPS- and nigericin-treated WT and Mut p85 cells. Mut p85 cells exhibited increased neutrophil respiratory burst compared with WT p85 cells (*n* = 8/group) (Figure 3D). Two cytokines, IL-1β and IL-8, are powerful inducers of chemotaxis in neutrophils (8–11). Although IL-8 had a similar effect in WT and Mut p85 cells, IL-1β increased the migration of Mut p85 cells compared with WT cells in a dose-dependent manner (Figure 3E and Supplemental Figure 3F). Consistent with this, Mut p85 cells also had higher transcript levels of cell migration–associated genes compared with WT p85 cells (Supplemental Figure 3E). Prior work demonstrated that AKT, the kinase downstream of PI3K, can induce the expression of IL-1R1 (12). Indeed, Mut p85 cells expressed higher levels of the IL-1R1 transcript and protein than did WT p85 cells (Figure 3, F and G). Furthermore, treatment with an IL-1R1 antagonist (IL-1RA), anakinra, blocked IL-1β–mediated migration of Mut p85 cells (Figure 3H). Finally, LPS- and nigericin-treated Mut p85 cells did not secrete more IL-1β protein than WT p85 cells (Supplemental Figure 3G).

Activated PI3KΔ syndrome (APDS) type 2 results from germline gain-of-function PIK3R1 mutations that activate both mTORC1 and mTORC2. We introduced the canonical p85 deletion mutant (amino acids 434–475) into HL-60 cells (13), and differentiated cells did not exhibit increased migration toward IL-1β (Supplemental Figure 4A). We also noted that Mut p85 promyeloblasts, the progenitor cell type for neutrophils, did not show increased phosphorylation of AKT at S473 compared with WT p85 promyeloblasts (Supplemental Figure 4B). These results may explain why our patient with refractory disease did not share a similar phenotype with APDS2 patients and had a normal bone marrow biopsy.

Taken together, we demonstrate that PIK3R1 p.W335C is a gain-of-function missense mutation that activates the PI3K pathway specifically through mTORC2, enhances neutrophil respiratory burst, and increases cell migration toward IL-1β.

### Clinical remission of refractory Sweet syndrome with anti–IL-1R1 antagonist.

**Figure 4:**
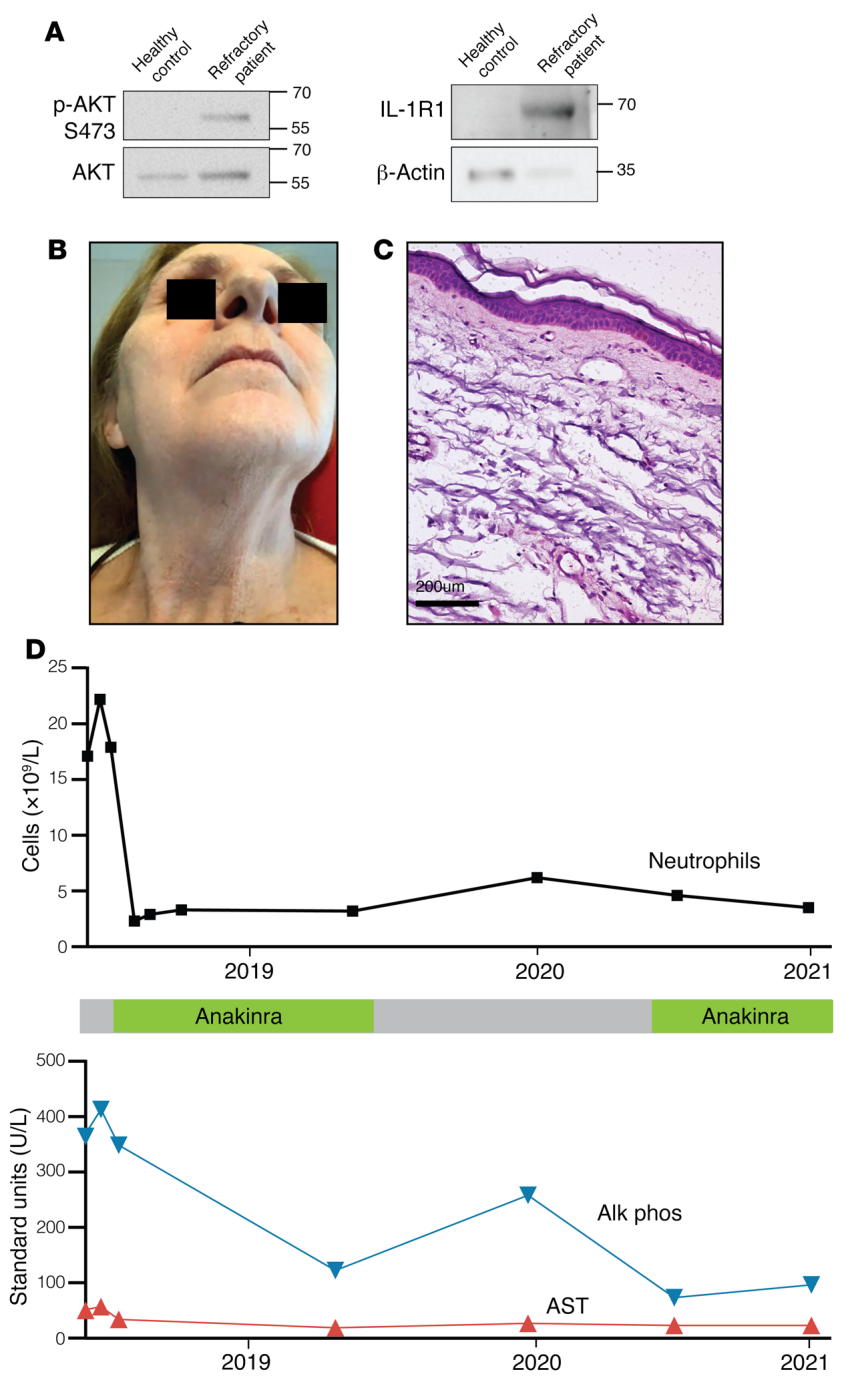
Clinical remission of refractory Sweet syndrome with an IL-1RA. (**A**) Neutrophils from the patient with refractory Sweet syndrome exhibited increased AKT activation and IL-1R1 expression. The experiment repeated 2 times. (**B**) Photograph shows disease clearance following anakinra treatment. (**C**) H&E staining of post-treatment skin biopsy with an absence of neutrophilic infiltration. (**D**) Neutrophil and liver enzyme counts for the patient with refractory disease after initiation of anakinra treatment. When anakinra was paused, her clinical symptoms, reflected in laboratory values, returned over several days.

We confirmed that primary neutrophils from the refractory patient also had increased p-AKT (S473) and IL-1R1 protein levels (Figure 4A). Neutrophils and macrophages differentiate from the granulocyte-monocyte progenitor cell. The p.W335C mutation was not detected in primary macrophages from the patient with refractory disease by Sanger sequencing (Supplemental Figure 5). We concluded that this mutation is strictly within the neutrophil lineage. These results suggested that IL-1R1 blockade could benefit our patient with refractory Sweet syndrome. Remarkably, treatment with anakinra achieved a dramatic therapeutic response with complete resolution of symptoms and allowed tapering off of all systemic corticosteroids (Figure 4, B–D). Notably, upon trials of stopping anakinra, the patient’s disease flared within days, with recurrence of fevers, skin lesions, neutrophilia, liver enzyme elevation, and right upper-quadrant pain. Re-initiation of therapy again led to complete disease remission (Figure 4D). Notably, we isolated neutrophils after anakinra treatment and still detected the p.W335C mutation (Figure 3B). Since starting anakinra, the patient has not experienced any further flares or hospitalizations, nor has she required additional corticosteroid use over the past 3 years. Given our findings, we queried her family history and found no family members with Sweet syndrome.

## Discussion

We describe the use of transcriptomics and genomic analysis to identify a gain-of-function PIK3R1 mutation in neutrophils from a patient with Sweet syndrome. The mutation increased the expression of IL-1R1, cell migration toward IL-1β, and neutrophil respiratory burst. Patients with Sweet syndrome typically exhibit pathergy, which is the induction of dermatosis-associated skin lesions at the site of minor skin trauma. Although the molecular mechanism causing pathergy remains undetermined, we speculate that incidental trauma triggers the release of additional IL-1 from injured cells and creates a signaling gradient that induces the recruitment of IL-1–sensitive, PIK3R1-mutant neutrophils to the skin. These insights led to successful treatment with an IL-1β antagonist, which blocked PIK3R1-mutant neutrophils from being recruited to the skin.

Dysregulated PI3K/AKT signaling is the first signaling pathway linked to Sweet syndrome. PIK3R1 mutations are associated with genetic and acquired human diseases (13–17), and this particular mutation has not to our knowledge been previously identified. As mentioned above, germline gain-of-function PIK3R1 mutations may cause APDS2 syndrome, but these mutations are generally exon-skipping, splice-site, or deletion variants (13, 18). The canonical APDS2 deletion mutant in our neutrophil-like cell line did not increase IL-1β–mediated cell migration. Cell-type specific and biochemical differences may explain the lack of phenotypic overlap between patients with APDS2 and our patient. Additional work is needed to understand how PIK3R1 specifically activates the mTORC1 and mTORC2 complexes. Finally, FDA-approved mTOR inhibitors, including sirolimus, are used to treat PI3K-mediated processes. However, these inhibitors specifically target mTORC1, and we do not believe they will be effective in this subset of patients with mTORC2-dominant Sweet syndrome.

This study counters current dogma that Sweet syndrome is a reactive process, where an unidentified signal activates normal neutrophils and recruits them to the skin. The finding of a somatic mutation that confers sensitivity to IL-1β demonstrates that Sweet syndrome may be caused by a cell-intrinsic process. Sweet syndrome is a member of a larger group of noninfectious neutrophilic dermatoses, including pyoderma gangrenosum, pustular psoriasis, and Behcet’s disease. Patients with Behcet’s disease and pyoderma gangrenosum have been reported to harbor somatic mutations in *NFKB1* that modulate IL-1β production (19–21). And patients with germline errors of immunity may also develop neutrophilic dermatoses, including mutations in *PSTPIP1*, *NLRP3*, and *IL36RN* (22). Thus, a common theme emerges from this highly analogous data that neutrophilic dermatoses may result from inherited or acquired mutations affecting signaling pathways that modulate neutrophil function. Some cases of Sweet syndrome are associated with malignancy, most commonly hematologic disease (1, 2). The finding of a somatic mutation without a concurrent malignancy is an important distinction in this case. Future patients with Sweet syndrome will benefit from a systematic approach to identify these potential mutations.

How does this mutation only affect neutrophils? As mentioned earlier, neutrophils and macrophages share a common progenitor cell, and patient primary macrophages did not harbor the mutation. Myeloblasts are the common progenitor cell type for neutrophils, basophils, and eosinophils and represent the cell of origin for myeloproliferative neoplasms. Our patient lacked overt changes to her basophil or eosinophil populations. Neutrophil-specific diseases do exist, including chronic neutrophilic leukemia, a rare but lethal disorder involving increased neutrophil infiltration and proliferation in the blood, bone marrow, and other organs. A subset of these patients carries activating point mutations in the *CSF3R* gene (23). Therefore, genetic mutations specifically targeting neutrophils may occur in a cell-specific stem cell population downstream from the myeloblast stage. In our patient with refractory Sweet syndrome, the return of clinical symptoms when pausing anakinra confirmed that her circulating neutrophils had a permanent sensitivity to IL-1β. It is interesting that mutated promyeloblasts carrying our missense mutation did not exhibit increased AKT activity, and more work is needed to understand how neutrophil differentiation uncovers this change.

Sequencing of the patient’s neutrophils revealed a single T nucleotide peak in the *PIK3R1* gene at position 335, and sequencing of the patient’s macrophages and skin revealed a single G nucleotide peak (Figure 3B, Supplemental Figure 5, and Supplemental Table 1). The absence of a heterozygous gain-of-function mutation was unusual. There are 2 possible explanations for this difference. The first, that the patient acquired the same somatic mutation in both alleles of the *PIK3R1* gene, is less likely. More likely is that the patient developed a mutation in a single allele of the *PIK3R1* gene within a neutrophil progenitor cell. This change conferred a selective advantage and resulted in the eventual loss of the second allele. Similar loss of heterozygosity is frequently identified in dysplastic syndromes and cancers (24). This finding suggests that additional screening of the patient’s neutrophil counts over time may be warranted, and more work is needed to understand this mechanism.

Increased IL-1β transcript levels were seen in 3 of the 7 other patients with Sweet syndrome, and some patients may exhibit increased circulating IL-1β (25–28). These data suggest that increased IL-1β signaling may play a role in a subset of patients with Sweet syndrome. Indeed, IL-1 blockade has been reported anecdotally to be effective in other cases of refractory disease (25–28). Nevertheless, a limitation of this study is that our findings were in a single patient and thus need to be extended to a larger cohort.

In summary, integration of molecular data across multiple levels helped identify a distinct pathotype within a heterogeneous disease that resulted in a successful clinical intervention enabling disease control. This rational and personalized therapy is, to our knowledge, the first example of identification of a targeted treatment for Sweet syndrome. The ability to directly interrogate diseased skin allows this approach to be generalizable to other inflammatory disorders.

## Methods

### Transcriptomic analysis.

LCM was performed on formalin-fixed, paraffin-embedded (FFPE) blocks to capture the dermis from 10 μm sections. RNA was isolated using the AllPrep DNA/RNA FFPE Kit (QIAGEN). RNA (10 ng) was provided to the University of Pennsylvania Genomic Analysis Core for downstream processing. cDNA was prepared and biotinylated using the GeneChip WT Pico Reagent Kit (Thermo Fisher Scientific). Clariom D Human array plates were used for hybridization of labeled cDNA followed by washing and staining and then scanned using the GeneChip 3000 7G system (Thermo Fisher Scientific). CEL files were generated after quality control and robust multiarray average (RMA). Further analysis was performed using Transcriptome Analysis Console (TAC) software. The filtering criteria for the transcriptional network for the differentially expressed genes were a FDR of less than 0.1 and a FC of greater than 1.5 or less than –1.5.

For qPCR, RNA isolation was performed using the RNeasy Mini Kit (QIAGEN). RNA (1 μg) was converted to cDNA by SuperScript IV VILO (Invitrogen, Thermo Fisher Scientific). Samples were run on a 7900HT Fast Real-Time PCR System (Applied Biosystems) with Sybr Green Universal PCR Master Mix (Thermo Fisher Scientific). The primer sequences are listed in Supplemental Table 3.

### Sequencing.

Infiltrating dermal neutrophils and nonlesional epidermis were isolated by LCM from FFPE sections. Genomic DNA was isolated using the DNeasy Blood and Tissue Kkit (QIAGEN). Sequencing was performed at the Beijing Genome Institute USA (BGI), where sample quality control, library construction using TruSeq DNA PCR-Free Library Prep Kit, and sequencing using DNBSEQ sequencing technology were performed. The sequencing depth was 30×, and bioinformatics analysis was performed by BGI. For Sanger sequencing, peripheral blood neutrophils were isolated using dextran sedimentation and Ficoll-Paque density gradients. Genomic DNA was isolated using the DNeasy Blood and Tissue Kit (QIAGEN), and PCR amplification of the PIK3R1 genomic region were performed. Sanger sequencing was performed at the University of Pennsylvania core facility.

### Cell culturing.

HL-60 cells, a human promyeloblast cell line, were obtained from the American Type Culture Collection (ATCC). Cells were grown in IMDM (catalog 12440053, Thermo Fisher Scientific) supplemented with 10% FBS and penicillin/streptomycin. For differentiation into a neutrophil-like state, HL-60 cells were treated with 1.3% DMSO in complete IMDM media for 5 days.

### Flow cytometry.

Flow cytometric analysis was performed with CD11b-APC (catalog 301329, BioLegend) and CD14-FITC (catalog 325603, BioLegend) to validate HL-60 cell differentiation as previously described (7). Cells were collected on day 5 for Western blot analysis and migration assays. Data were analyzed with FlowJo software, version 10 (Tree Star).

### Generation of PIK3R1-overexpressing cell lines.

pBluescriptR plasmid containing the human full-length PIK3R1 coding sequence (Horizon Discovery, clone ID: 30528412) was purchased. PIK3R1 was cloned into a lentiviral FUW plasmid (Addgene, plasmid 14882). A Q5 Site-Directed Mutagenesis Kit (New England BioLabs [NEB]) was used to incorporate the G335T mutation into the PIK3R1 coding sequence, and the mutation was confirmed by PCR amplicon sequencing. Lentiviral transduction was performed using standard methods. HL-60 cells were infected with lentivirus in the presence of 8 μg/mL polybrene (MilliporeSigma) for 48 hours. Antibiotic selection with Zeocin (200 μg/mL for 7 days) was used to select positive clones.

### Cellular functional assays.

Cell survival was assessed by a trypan blue dye exclusion assay on days 3 to 6 after DMSO addition to HL-60 cells. A Countess Automated Cell Counter (Thermo Fisher Scientific) was used to quantify live cells. Cell proliferation was assessed by a CyQuant direct cell proliferation assay (catalog C35011, Thermo Fisher Scientific). A Transwell migration assay was performed on differentiated HL-60 cells on day 5. The top chamber of the Transwell contained cells in IMDM media with DMSO, and the bottom chamber contained IMDM supplemented with IL-1β or IL-8. To inhibit migration, IL-1RA (catalog SRP3084, MilliporeSigma) was added to the top chamber. The number of live cells in the bottom chamber was quantified at 16 to 18 hours by a trypan blue dye exclusion assay. Cellular respiratory burst was detected using the cell-permeable redox-sensitive fluorescent probe DCFDA according to the manufacturer’s protocol (Cayman Industries). Briefly, 20 × 10^3^ cells were seeded in a 96-well plate. Cells were treated with 100 ng/mL LPS for 4 hours followed by 10 μM nigericin for 1 hour. H_2_O_2_ (100 μM) for 1 hour served as a positive control. Cells were washed with PBS and incubated with 20 μM DCFDA for an hour. Cells were washed with PBS to remove unassimilated DCFDA, and fluorescence was read on a plate reader: excitation, 485/20, emission, 528/20 (BioTekHT). IL-1β secretion was detected by ELISA (R&D Systems). Cells were treated with 100 ng/mL LPS for 4 hours followed by 10 μM nigericin for 1 hour.

### Western blot analysis.

Whole-cell lysates were prepared using RIPA buffer with PMSF and protease and phosphatase inhibitors (Invitrogen, Thermo Fisher Scientific). Total cell lysate (30 μg) was used for protein quantification by Pierce BCA protein assay (catalog 23225, Thermo Fisher Scientific). The cell lysate was resolved using 4%–12% NuPage gels (Thermo Fisher Scientific) and transferred onto a nitrocellulose membrane using a wet transfer system (Bio-Rad). After blocking in 5% milk, membranes were incubated with a primary antibody overnight at 4°C. The following primary antibodies were used: anti-AKT (1:1,000, catalog 9272, Cell Signaling Technology); anti–p-AKT (1:1,000, catalog 9271, Cell Signaling Technology); anti–S6 kinase (1:1,000, catalog 9202, Cell Signaling Technologies); anti–p-S6 kinase (1:1,000, catalog 9204, Cell Signaling Technology); anti–β-actin (1:5,000, catalog 4970, Cell Signaling Technology); anti–PI3K p85a (1:1,000, catalog 60225, Proteintech); and anti–IL-1R1 (1:2,000, catalog PA5-97866, Thermo Fisher Scientific). Membranes were washed and incubated with an anti-rabbit or mouse HRP-linked secondary antibody (anti-rabbit, catalog 7074S, Cell Signaling Technology; anti-mouse, catalog sc-2005, Santa Cruz Biotechnology) and were visualized by chemiluminescence using an ECL prime kit (catalog RPN2232, MilliporeSigma) and the ChemiDoc imaging system (Bio-Rad Laboratories). Densitometric analysis was performed using ImageLab software (Bio-Rad).

### Data and materials availability.

Microarray data were deposited in the NCBI’s Gene Expression Omnibus (GEO) database (GEO GSE190335).

### Statistics.

The data presented in this study combined all experiments, and unless noted, all experiments were repeated 3–5 times independently. Experiments were not randomized, and investigators were not blinded to allocation during the experiments and outcome assessments, unless noted in the text. A 2-tailed Student’s *t* test was used to determine significance, with *P* values of less than 0.05 considered significant (with **P < 0.01 and ***P < 0.001 indicating higher levels of significance). When appropriate, specific *P* values are provided in figure legends.

### Study approval.

The study protocol was approved by the IRB of the University of Pennsylvania School of Medicine. All patients provided written informed consent prior to participation in the study. The funding sources did not participate in any part of the trial, from conception through manuscript preparation. The diagnosis of Sweet syndrome was made by trained dermatologists and dermatopathologists using published criteria based on clinical and histologic findings. Within the informed consent, we explicitly explained that our protocol was for discovery work and that patients should have no expectation of a specific medical intervention, treatment, or altered approach to modify their health outcome. Blood and skin biopsies were obtained from the participants. Healthy control skin samples were deidentified discarded tissue obtained from the Skin Biology and Disease Resource Center at the University of Pennsylvania. Written informed consent was provided for clinical photographs appearing in the manuscript.

## Author contributions

CAN, JSB, HAA, KS, AF, JTS, RGM, WDJ, and MR collected clinical samples. S Bhattacharya, S Basu, ES, CM, JW, AEK, and THL conceptualized the experiments, designed the methodology, and performed experiments. S Bhattacharya and THL wrote the manuscript. S Bhattacharya, S Basu, CAN, JSB, KS, RGM, WDJ, and MR reviewed and edited the manuscript.

## Supplementary Material

Supplemental data

ICMJE disclosure forms

## Version 1 11/10/2022

In-Press Preview

## Version 2 01/03/2023

Electronic publication
