# Supplementary material for: Identification of a neutrophil-specific PIK3R1 mutation facilitates targeted treatment in a patient with Sweet syndrome
Source: J Clin Invest. 2023 Jan 3;133(1):e162137. doi: 10.1172/JCI162137 (PMC9797331; doi:10.1172/JCI162137)
Supplement: Supplemental data [file jci-133-162137-s090.pdf]

Supplementary Materials

Supplemental Figure 1. Refractory Sweet syndrome lesions revealed by clinical evidence and dominant IL1β inflammation.

Supplemental Figure 2. Differentiation of HL60 promyeloblasts to a neutrophil-like state.

Supplemental Figure 3. Refractory Sweet syndrome lesions reveal dominant IL1β inflammation.

Supplemental Figure 4. Characterization of p.W335C mutant p85.

Supplemental Figure 5. Refractory patient macrophages do not carry p.W335C PIK3R1 mutation.

Supplemental Table 1. Microarray profiling of refractory Sweet syndrome patient and healthy controls dermis.

Supplemental Table 2. Neutrophil-specific mutations in refractory Sweet Syndrome patient.

Supplemental Table 3. Primer sequences.

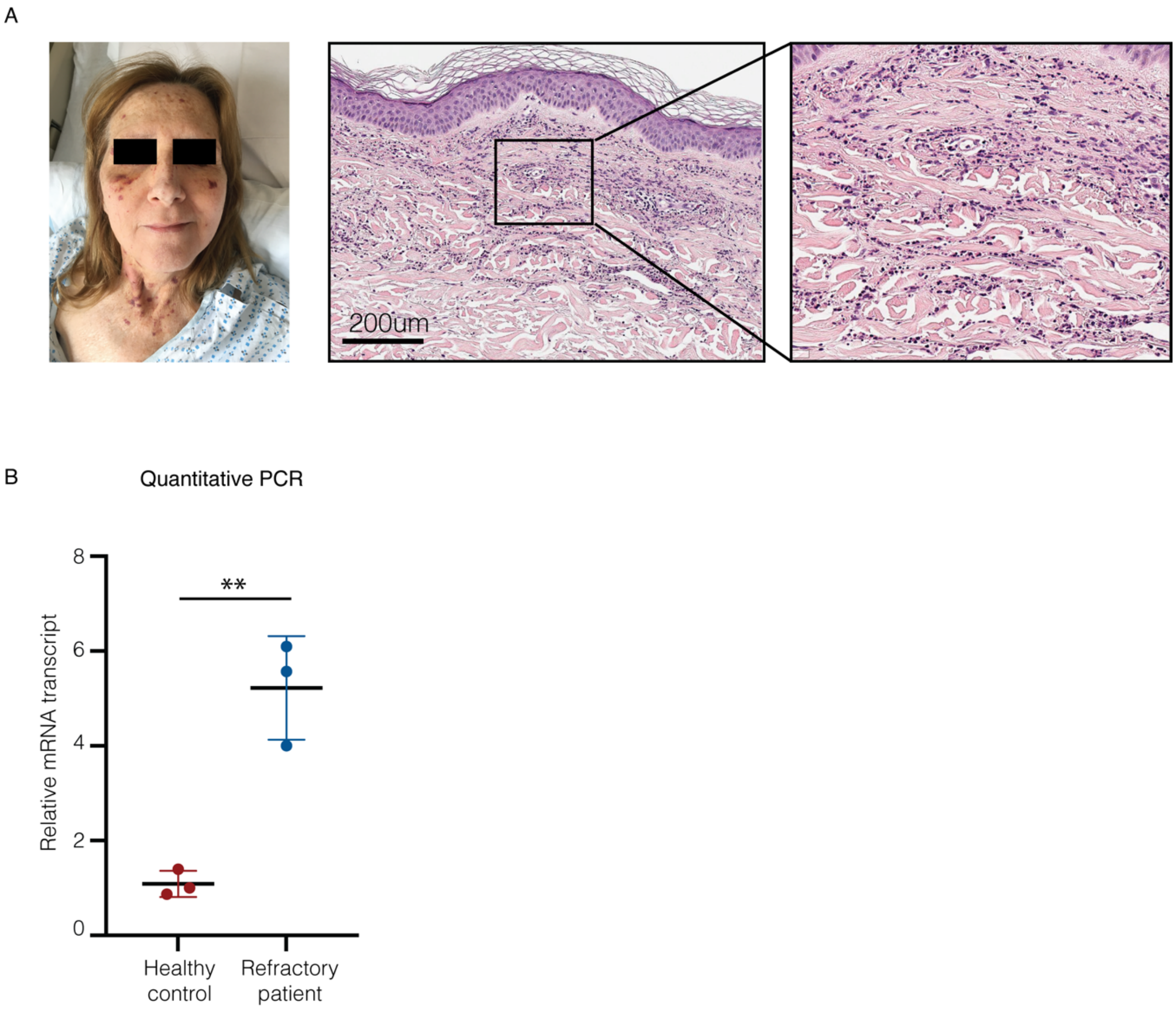

Supplemental Figure 1. Refractory Sweet syndrome lesions exhibit IL1β mediated inflammation.

(A) Additional patient photograph and a second hematoxylin and eosin stained skin biopsy that displays a diffuse neutrophilic infiltration in the dermis. (B) Increased IL1β transcript detected by quantitative-PCR in the dermis of the refractory patient compared to healthy control dermis (*n*=3 in each group). Data represents mean ± SEM. 2-tailed Student's t test. **P<0.01.

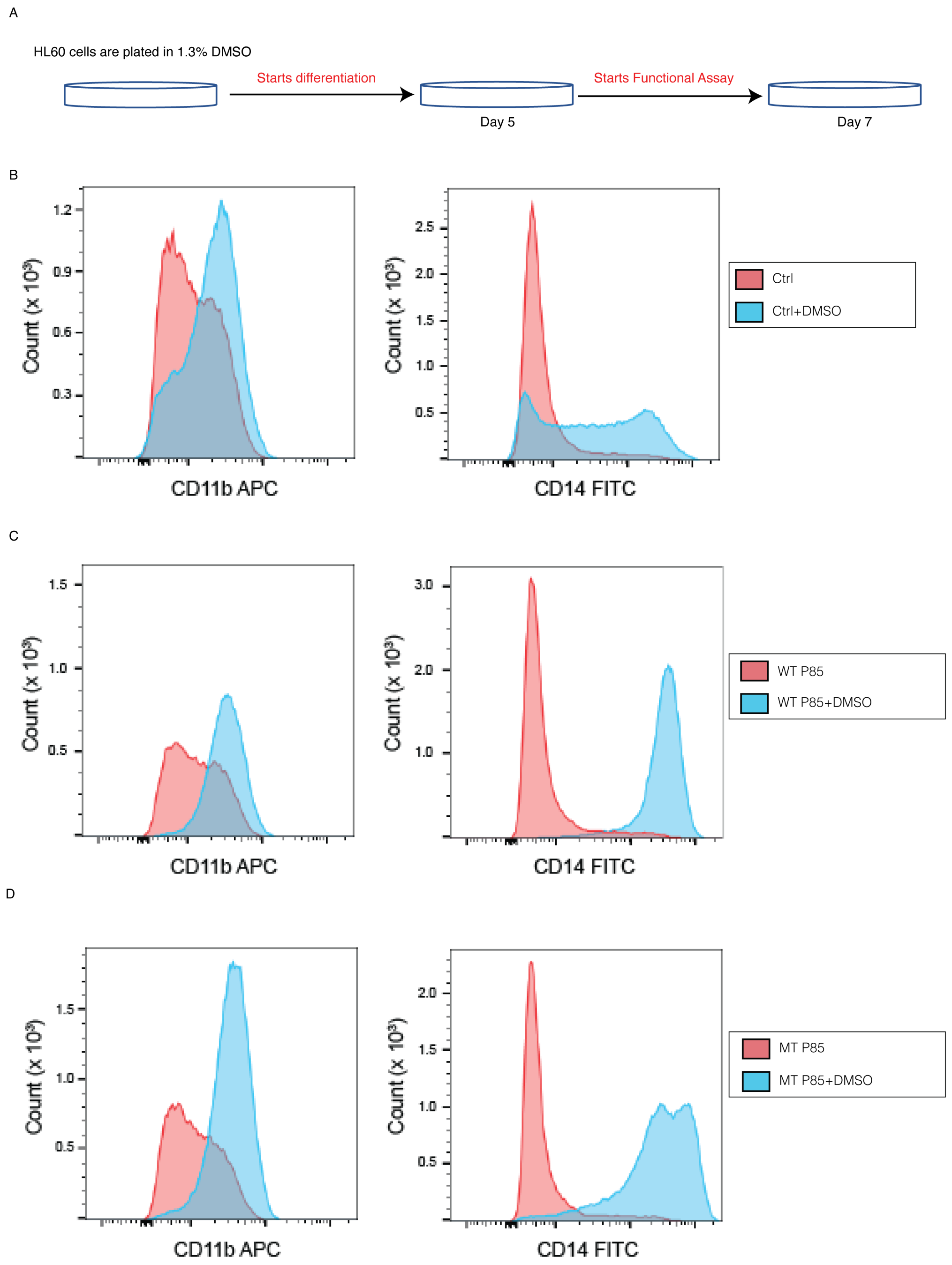

Supplemental Figure 2. Differentiation of HL60 promyeloblasts to a neutrophil-like state. (**A**) Schematic of HL60 differentiation. HL60 promyeloblasts were differentiated into a neutrophil-like state with DMSO treatment. Neutrophil-like state was assessed by flow cytometry and defined by increased expression of CD11b and CD14. (**B**)-(**D**) Flow cytometry analysis shows appropriate HL60 cell differentiation; control HL60 cells with and without DMSO treatment (B), wild-type p85 overexpressed HL60 cells with and without DMSO treatment (C), and W335C mutant p85 overexpressed HL60 cells with and without DMSO treatment (D). Experiment repeated twice independently.

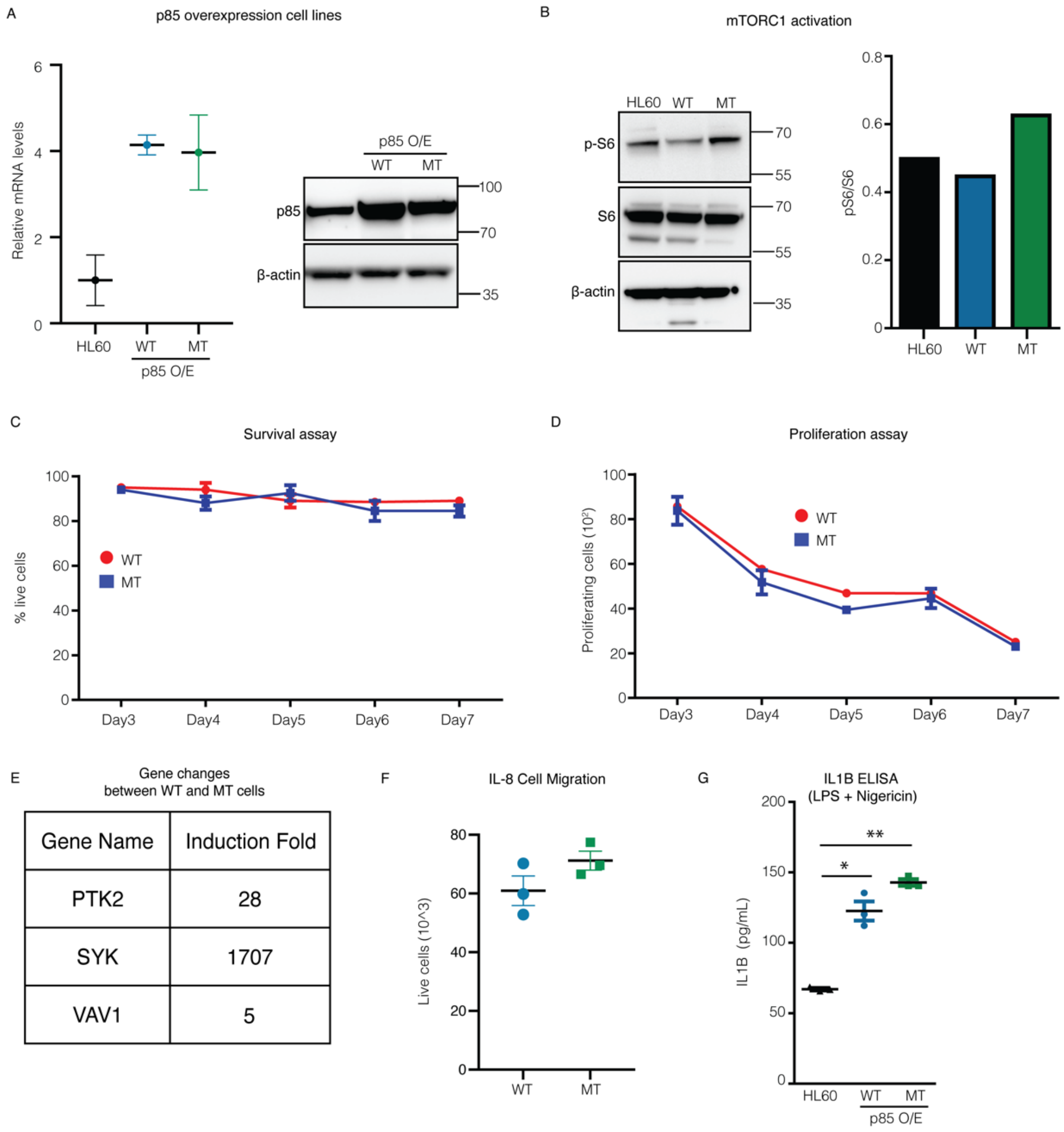

Gene Name	Induction Fold
PTK2	28
SYK	1707
VAV1	5

Supplemental Figure 3. Characterization of W335C mutant p85 overexpressed cells. (**A**) RNA transcript (*n*=3 in each group) and protein levels of p85 in wildtype (WT, blue) and W335C mutant (MT, green) p85 overexpressed HL60 cells. Control (black bar) are non-transduced HL-60 cells. (**B**) No difference in phosphorylated S6 protein was observed between WT and MT p85 cells. Control (black bar) are non-transduced HL-60 cells. (**C**) and (**D**) No difference in cell survival (Trypan Blue dye exclusion) or cell proliferation (CyQuant) between WT and MT p85 cells was detected (*n*=6 in each group). (**E**) Cell migration gene transcripts are induced in MT p85 cells compared to WT control. (**F**) No difference in Transwell migration towards IL-8 between WT and MT p85 cells (*n*=3 in each group). (**G**) IL-1β protein secretion measured in supernatant of LPS+Nigericin treated cells (*n*=3 in each group). All experiments were repeated independently 2-3 times. Data represents mean ± SEM. 2-tailed Student's t test. *P<0.05; **P<0.01.

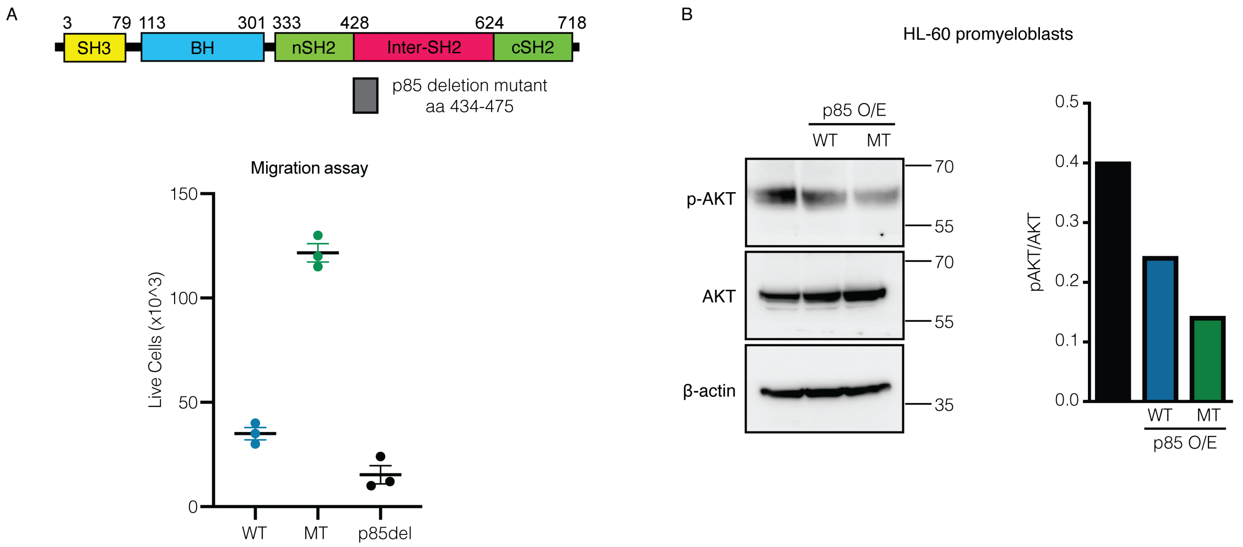

Supplemental Figure 4. Characterization of p.W335C mutant.

(**A**) Differentiated HL-60 cells carrying p85 deletion (aa434-475, p85del) do not exhibit increased cell migration towards IL1β (*n*=3 in each group). (**B**) Representative image demonstrating no difference in phosphorylated AKT protein in undifferentiated wild-type (WT) and W335C mutant (MT) p85 overexpressed HL60 cells. Control (black bar) are non-transduced HL-60 cells. The right panel is protein quantification. All experiments were repeated independently 2-3 times.

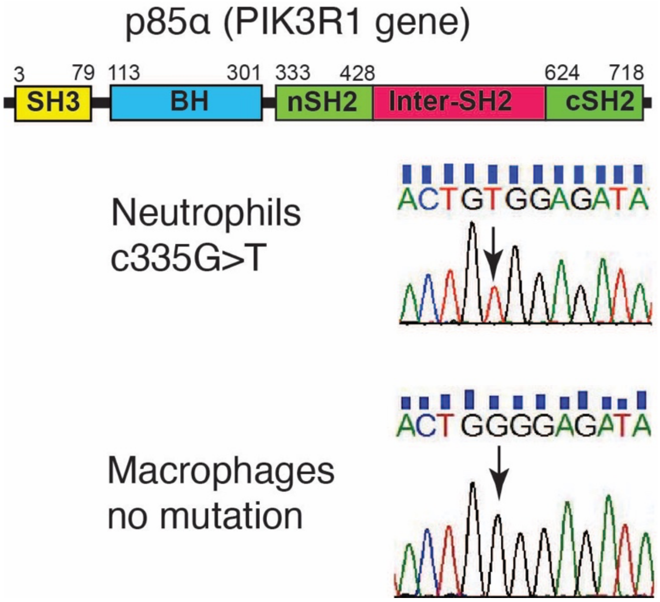

Supplemental Figure 5. Refractory patient macrophages do not carry the p.W335C PIK3R1 mutation. Sanger sequencing of patient macrophages.

Supplemental Table 1: Microarray profiling of Refractory Sweet Syndrome patient and Healthy Controls dermis

#%annotation=Clariom_D_Human.r1.na36.hg38.a1.transcript.csv

#%comparison= Refractory Sweet Syndrome vs Healthy_Control

ID	Refractory Sweet Syndrome Avg (log2)	Healthy_ Control Avg (log2)	Fold Change	P-val	FDR P-val	Gene Symbol	Description	Group
TC1000010870.hg.1	16.4	7.94	352.82	1.05E-08	0.0002	SRGN	Memczak2013 ANTISENSE, coding, INTERNAL, UTR3 best transcript NM_002727	NonCoding
TC0400011052.hg.1	11.93	3.99	246.4	1.29E-05	0.0126	CXCL9	chemokine (C-X-C motif) ligand 9	Coding
TC0X00007149.hg.1	18	11.05	123.8	0.0012	0.0985	TIMP1	TIMP metallopeptidase inhibitor 1	Multiple_Complex
TC1900008869.hg.1	15.41	9.04	82.81	4.09E-10	4.90E-05	AC245036.6		Precursor_microRNA
TC0100009403.hg.1	11.84	6.15	51.39	2.24E-06	0.0051	CHI3L2	chitinase 3-like 2	Multiple_Complex
TC1200012749.hg.1	13.77	8.2	47.43	0.0004	0.0667	CD163	CD163 molecule	Multiple_Complex
TC0100010032.hg.1	11.47	5.93	46.63	1.82E-06	0.0046	S100A9	S100 calcium binding protein A9	Multiple_Complex
TC1200008133.hg.1	12.85	7.33	45.77	5.31E-05	0.0261	LYZ	lysozyme	Multiple_Complex
TC1900008871.hg.1	14.33	8.82	45.53	1.44E-09	4.90E-05	AC245036.5		Precursor_microRNA
TC1900008872.hg.1	14.33	8.82	45.53	1.44E-09	4.90E-05	AC245036.4		Precursor_microRNA
TC1900008873.hg.1	14.33	8.82	45.53	1.44E-09	4.90E-05	AC245036.2		Precursor_microRNA
TC0X00009981.hg.1	11.22	5.85	41.54	4.42E-07	0.002	IL2RG	interleukin 2 receptor, gamma	Multiple_Complex
TC0100010341.hg.1	11.08	5.81	38.64	1.56E-05	0.0142	FCER1G	Fc fragment of IgE, high affinity I, receptor for; gamma polypeptide	Multiple_Complex
TC0100009394.hg.1	14.06	8.95	34.62	6.80E-06	0.0092	CD53	CD53 molecule	Multiple_Complex
TC0500007138.hg.1	9.11	4.33	27.57	0.0002	0.0495	IL7R	interleukin 7 receptor	Multiple_Complex
TC0700012183.hg.1	10.6	6.31	19.57	3.47E-05	0.0204	sparblee	Transcript Identified by AceView	Unassigned
TC1900008870.hg.1	12.36	8.28	16.91	1.24E-08	0.0002	AC245036.3		Precursor_microRNA
TC0700010774.hg.1	8.78	4.76	16.21	5.23E-05	0.0258	TRGJP1	T cell receptor gamma joining P1	Coding
TC0900007947.hg.1	10.54	6.58	15.51	0.0002	0.0452	stertuby	Transcript Identified by AceView	Unassigned
TC0100015753.hg.1	11.86	7.94	15.17	5.07E-06	0.0079	smobee	Transcript Identified by AceView	Unassigned
TC0100014857.hg.1	8.1	4.28	14.15	5.67E-05	0.0272	GBP5	guanylate binding protein 5	Multiple_Complex
TC1600006890.hg.1	8.4	4.6	14	0.0005	0.0699	zeesmey	Transcript Identified by AceView	Coding
TC0200015242.hg.1	7.8	4.22	11.92	0.0005	0.0679	STAT1	signal transducer and activator of transcription 1	Multiple_Complex
TC0600007598.hg.1	10.01	6.49	11.49	7.88E-05	0.0315	AIF1	allograft inflammatory factor 1	Multiple_Complex

TC1900008874.hg.1	12.03	8.53	11.31	1.32E-07	0.001	MIR8061	microRNA 8061	Precursor_ microRNA
TC1400010195.hg.1	10.14	6.64	11.24	1.24E-05	0.0124	WARS	tryptophanyl-tRNA synthetase	Multiple_Co mplex
TC0300012169.hg.1	11.9	8.42	11.17	0.0005	0.0697	RNU4-62P	RNA, U4 small nuclear 62, pseudogene [Source:HGNC Symbol;Acc:HGNC:469 98]	Small_RNA
TC1700007557.hg.1	8.31	4.86	10.94	9.65E-07	0.0034	CCL2	chemokine (C-C motif) ligand 2	Multiple_Co mplex
TC1100007510.hg.1	10.8	7.38	10.73	0.0002	0.0467	keeny	Transcript Identified by AceView	Coding
TC0900011308.hg.1	8.68	5.3	10.4	6.12E-06	0.0085	foshor	Transcript Identified by AceView	Unassigned
TC0100018529.hg.1	6.76	3.56	9.17	5.09E-05	0.0255	SELE	selectin E	Multiple_Co mplex
TC0300012815.hg.1	8.12	4.94	9.08	2.07E-06	0.0048	P2RY13	purinergic receptor P2Y, G-protein coupled, 13	Coding
TC0100010310.hg.1	7.26	4.08	9.05	4.65E-05	0.0243	SLAMF7	SLAM family member 7	Multiple_Co mplex
TC0700012182.hg.1	9.15	5.98	8.95	0.0004	0.0636	NAMPT	nicotinamide phosphoribosyltransfer ase	Multiple_Co mplex
TC0600013231.hg.1	10.13	6.98	8.88	0.0013	0.1	SGK1	serum/glucocorticoid regulated kinase 1	Multiple_Co mplex
TC0500010553.hg.1	8.89	5.75	8.8	0.0003	0.0523	FYB	FYN binding protein	Multiple_Co mplex
TC0500011583.hg.1	6.54	3.41	8.79	0.0008	0.0829	saneme	Transcript Identified by AceView	Coding
TC1300008760.hg.1	9.56	6.49	8.39	9.06E-05	0.0336	EPSTI1	epithelial stromal interaction 1 (breast)	Multiple_Co mplex
TC1400010721.hg.1	9.69	6.63	8.32	1.88E-05	0.0156	PSME2; MIR7703	proteasome activator subunit 2; microRNA 7703	Multiple_Co mplex
TC1300008842.hg.1	8.34	5.34	8.02	3.09E-06	0.0059	chufoby	Transcript Identified by AceView	Unassigned
TC0600007597.hg.1	9.83	6.84	7.94	1.16E-07	0.0009	LST1	leukocyte specific transcript 1	Multiple_Co mplex
TC1200007251.hg.1	10.85	7.91	7.69	0.0005	0.0679	KIAA1551	KIAA1551	Multiple_Co mplex
TC0500008943.hg.1	11.67	8.79	7.38	8.56E-07	0.0031			NonCoding
TC0100015858.hg.1	6.88	4.03	7.24	4.38E-05	0.0232	S100A8	S100 calcium binding protein A8	Multiple_Co mplex
TC1400006623.hg.1	7.52	4.66	7.23	0.0002	0.0452	TRAJ56	T cell receptor alpha joining 56	Coding
TC0500012470.hg.1	9.6	6.83	6.8	7.09E-05	0.0303	CD74	CD74 molecule, major histocompatibility complex, class II invariant chain	Multiple_Co mplex
TC1100010733.hg.1	8.39	5.74	6.25	0.0004	0.0635	SPI1	Spi-1 proto-oncogene	Multiple_Co mplex
TC0300008542.hg.1	7.96	5.34	6.11	4.44E-06	0.0072	CD86	CD86 molecule	Multiple_Co mplex
TC1600009683.hg.1	6.74	4.14	6.07	1.60E-06	0.0043	IGSF6	immunoglobulin superfamily, member 6	Multiple_Co mplex
TC1800007680.hg.1	11.09	8.5	6.01	0.0002	0.0444	CNDP2	CNDP dipeptidase 2 (metallopeptidase M20 family)	Multiple_Co mplex
TC0400007836.hg.1	8.22	5.68	5.81	0.0004	0.0629	CXCL8	chemokine (C-X-C motif) ligand 8	Multiple_Co mplex
TC0300007259.hg.1	6.89	4.41	5.58	3.18E-09	8.65E-05	CCRL2	chemokine (C-C motif) receptor-like 2	Multiple_Co mplex
TC0100009866.hg.1	5.99	3.51	5.57	2.10E-08	0.0003	FCGR1A	Fc fragment of IgG, high affinity Ia, receptor (CD64)	Multiple_Co mplex
TC0100010245.hg.1	7.13	4.69	5.42	2.16E-05	0.0165			NonCoding

TC0300013336.hg.1	6.96	4.52	5.41	3.81E-07	0.002	LAMP3	lysosomal-associated membrane protein 3	Multiple_Complex
TC0800007978.hg.1	7.94	5.54	5.25	0.0008	0.0817	LY96	lymphocyte antigen 96	Coding
TC0800006980.hg.1	6.9	4.54	5.14	1.49E-06	0.0043	SLC39A14	solute carrier family 39 (zinc transporter), member 14	Multiple_Complex
TC1600007957.hg.1	10	7.66	5.06	0.0004	0.0671	MT2A	metallothionein 2A	Multiple_Complex
TC1100009191.hg.1	6.57	4.24	5.03	0.0004	0.0658	IL10RA	interleukin 10 receptor, alpha	Multiple_Complex
TC0800011822.hg.1	7.79	5.53	4.82	0.0003	0.0555	RNU7-181P	RNA, U7 small nuclear 181 pseudogene [Source:HGNC Symbol;Acc:HGNC:45715]	Small_RNA
TC1400007890.hg.1	6.58	4.36	4.68	0.0002	0.0452	GPR65	G protein-coupled receptor 65	Coding
TC0100009417.hg.1	7.12	4.91	4.61	3.80E-05	0.0215	C1orf162	chromosome 1 open reading frame 162	Multiple_Complex
TC1600007241.hg.1	6.26	4.06	4.59	1.32E-06	0.004			NonCoding
TC2100008000.hg.1	11.84	9.65	4.55	8.03E-06	0.01	GART	phosphoribosylglycinamide formyltransferase, phosphoribosylglycinamide synthetase, phosphoribosylaminoimidazole synthetase	Multiple_Complex
TC1700008984.hg.1	6.64	4.46	4.52	3.20E-07	0.0019	SEC14L1; SCARNA16; SNHG20; MIR6516	SEC14-like lipid binding 1; small Cajal body-specific RNA 16; small nucleolar RNA host gene 20; microRNA 6516	Multiple_Complex
TC0600010816.hg.1	6.58	4.41	4.52	3.24E-06	0.0059			NonCoding
TC0500013400.hg.1	6.47	4.32	4.43	2.53E-06	0.0054			NonCoding
TC0200014693.hg.1	6.58	4.43	4.43	0.0003	0.056	CYTIP	cytohesin 1 interacting protein	Multiple_Complex
TC1200011845.hg.1	6	3.85	4.43	8.64E-09	0.0002	SELPLG	selectin P ligand	Coding
TC1600007243.hg.1	7.93	5.78	4.41	1.87E-05	0.0156	stoyweebu	Transcript Identified by AceView	Coding
TC0200013342.hg.1	6.56	4.46	4.28	0.0006	0.073	AC133644.2	novel transcript	NonCoding
TC0X00006626.hg.1	5.56	3.46	4.28	1.25E-05	0.0124	TLR8	toll-like receptor 8	Coding
TC1100012478.hg.1	5.26	3.18	4.21	1.98E-07	0.0013	CD3D	CD3d molecule, delta (CD3-TCR complex)	Multiple_Complex
TC0700007430.hg.1	8.7	6.66	4.1	0.0012	0.0964	RNU6-241P	RNA, U6 small nuclear 241, pseudogene [Source:HGNC Symbol;Acc:HGNC:47204]	Multiple_Complex
TC0500008438.hg.1	6.86	4.83	4.09	0.0011	0.0945	teyslawby	Transcript Identified by AceView	Coding
TC0300011517.hg.1	5.81	3.79	4.05	1.68E-05	0.0147	PROK2	prokineticin 2	Coding
TC1100008330.hg.1	6.66	4.66	4	0.0008	0.0826	IL18BP	interleukin 18 binding protein	Multiple_Complex
TC1200012593.hg.1	6.61	4.61	4	3.26E-06	0.0059	CLEC4A	C-type lectin domain family 4, member A	Coding
TC2200008592.hg.1	5.48	3.54	3.85	9.47E-06	0.0106	APOL2	apolipoprotein L, 2	Multiple_Complex
TC2200007287.hg.1	8.52	6.59	3.82	0.0001	0.0412	CYTH4	cytohesin 4	Multiple_Complex
TC1600007530.hg.1	7.58	5.65	3.81	0.0005	0.0699	ITGAM	Transcript Identified by AceView, Entrez Gene ID(s) 3684	Unassigned
TC1700006638.hg.1	8.85	6.92	3.8	0.0004	0.0624	ARRB2	arrestin, beta 2	Multiple_Complex

TC1700009389.hg.1	8.35	6.43	3.8	0.0009	0.0877			NonCoding
TC0700013611.hg.1	5.13	3.23	3.72	7.29E-05	0.0307	TFEC	transcription factor EC	NonCoding
TC0700009677.hg.1	7.07	5.19	3.68	0.0002	0.0428	GIMAP4	GTPase, IMAP family member 4	Multiple_Complex
TC1300009598.hg.1	6.82	4.95	3.65	5.81E-05	0.0277	GPR183	G protein-coupled receptor 183	Coding
TC1400006618.hg.1	5.51	3.64	3.65	0.0006	0.0736	TRDJ2	T cell receptor delta joining 2	Coding
TC0100014852.hg.1	8.33	6.47	3.62	0.0002	0.0436	GBP1	guanylate binding protein 1, interferon-inducible	Multiple_Complex
TC0500009382.hg.1	7.54	5.68	3.61	0.0003	0.0604	soyskeeby	Transcript Identified by AceView	Unassigned
TC1700012388.hg.1	5.06	3.21	3.6	0.0004	0.0667			NonCoding
TC1500007357.hg.1	5.45	3.61	3.59	3.40E-06	0.006	MTND3P12	mitochondrially encoded NADH:ubiquinone oxidoreductase core subunit 3 pseudogene 12 [Source:HGNC Symbol;Acc:HGNC:52072]	Multiple_Complex
TC1200008689.hg.1	5.83	4	3.56	2.57E-07	0.0016	glyjawby	Transcript Identified by AceView	Unassigned
TC1700007591.hg.1	8.94	7.13	3.52	0.0003	0.0585	E2F3P1	Transcript Identified by AceView, Entrez Gene ID(s) 1872	Unassigned
TC0X00007962.hg.1	9.58	7.77	3.5	0.0002	0.0492	RNU6-345P	RNA, U6 small nuclear 345, pseudogene [Source:HGNC Symbol;Acc:HGNC:47308]	Small_RNA
TC1000010341.hg.1	6.05	4.25	3.5	0.0001	0.0396	NAMPTP1	nicotinamide phosphoribosyltransferase pseudogene 1 [Source:HGNC Symbol;Acc:HGNC:17633]	Pseudogene
TC0X00007033.hg.1	5.5	3.7	3.49	1.02E-05	0.0111			NonCoding
TC0500007154.hg.1	8.12	6.34	3.45	8.87E-05	0.0333	SLC1A3	solute carrier family 1 (glial high affinity glutamate transporter), member 3	Multiple_Complex
TC0900007115.hg.1	7.67	5.88	3.45	0.0005	0.0724	GLIPR2	GLI pathogenesis-related 2	Multiple_Complex
TC0600009597.hg.1	9.36	7.57	3.45	0.0002	0.0428	TNFAIP3	tumor necrosis factor, alpha-induced protein 3	Multiple_Complex
TC1200006957.hg.1	5.62	3.84	3.43	0.0001	0.0371	PTPRO	protein tyrosine phosphatase, receptor type, O	Multiple_Complex
TC0200015243.hg.1	7.92	6.15	3.42	1.22E-05	0.0124	mohuyo	Transcript Identified by AceView	Unassigned
TC0100009555.hg.1	5.64	3.87	3.4	0.0001	0.0359	CD2	CD2 molecule	Coding
TC0300007257.hg.1	5.32	3.56	3.4	9.37E-06	0.0106	CCR5	chemokine (C-C motif) receptor 5 (gene/pseudogene)	Multiple_Complex
TC0100010241.hg.1	6.18	4.41	3.39	0.0007	0.0806	MNDA	myeloid cell nuclear differentiation antigen	Multiple_Complex
TC0X00006452.hg.1	5.32	3.56	3.39	6.10E-05	0.028	serree	Transcript Identified by AceView	Coding
TC0Y00006450.hg.1	5.32	3.56	3.39	6.10E-05	0.028	varbo	Transcript Identified by AceView	Coding
TC1900008057.hg.1	5.94	4.18	3.39	0.0003	0.0529	ZFP36	ZFP36 ring finger protein	Coding
TC1000009296.hg.1	6.06	4.3	3.39	2.72E-05	0.0183	PTPRE	protein tyrosine phosphatase, receptor type, E	Multiple_Complex

TC1500010160.hg.1	5.41	3.66	3.37	0.0009	0.0881	CTSH	cathepsin H	Multiple_Complex
TC1700010314.hg.1	6.8	5.05	3.37	1.80E-05	0.0154	EVI2A; EVI2B	ecotropic viral integration site 2A; ecotropic viral integration site 2B	Coding
TC0X00007538.hg.1	8.94	7.19	3.37	6.96E-05	0.0299	RNY4P23	RNA, Ro-associated Y4 pseudogene 23 [Source:HGNC Symbol;Acc:HGNC:42473]	NonCoding
TC0400010449.hg.1	5.64	3.89	3.36	0.0005	0.0679	TLR10	toll-like receptor 10	Multiple_Complex
TC0X00006891.hg.1	4.94	3.22	3.3	3.19E-05	0.0192	GK	glycerol kinase	Multiple_Complex
TC1900008668.hg.1	5.89	4.17	3.29	6.97E-06	0.0092	FPR2	formyl peptide receptor 2	Coding
TC0900009490.hg.1	6.31	4.59	3.29	0.0001	0.0371			NonCoding
TC1600011060.hg.1	8.04	6.33	3.27	8.39E-06	0.0102	COTL1	coactosin-like F-actin binding protein 1	Multiple_Complex
TC1100007937.hg.1	6.13	4.43	3.24	0.0002	0.051	karsha	Transcript Identified by AceView	Coding
TC0400008861.hg.1	6.15	4.45	3.24	0.0002	0.0482	IL15	interleukin 15	Multiple_Complex
TC0500013401.hg.1	5.89	4.22	3.2	9.45E-05	0.0344	LCP2	lymphocyte cytosolic protein 2	Multiple_Complex
TC0800007688.hg.1	7.43	5.76	3.19	2.32E-06	0.0052	LYN	LYN proto-oncogene, Src family tyrosine kinase	Multiple_Complex
TC2000007686.hg.1	5.8	4.13	3.17	0.0002	0.0511	LINC01272	long intergenic non-protein coding RNA 1272	Multiple_Complex
TC0X00006958.hg.1	8.3	6.64	3.17	2.44E-05	0.0171	CYBB	cytochrome b-245, beta polypeptide	Multiple_Complex
TC1400010507.hg.1	5.02	3.36	3.17	1.02E-05	0.0111	IGHVIII-38-1	immunoglobulin heavy variable (III)-38-1 (pseudogene)	Multiple_Complex
TC1200010334.hg.1	7.62	5.96	3.15	1.13E-05	0.0119	RP11-278C7.4	novel transcript, antisense to DNM1L	NonCoding
TC1200008688.hg.1	6.97	5.32	3.14	3.03E-05	0.0186	tachaw	Transcript Identified by AceView	Coding
TC0300007067.hg.1	8.56	6.91	3.14	6.56E-08	0.0006	MYD88	myeloid differentiation primary response 88	Multiple_Complex
TC1000008927.hg.1	10.14	8.49	3.13	0.0008	0.0826	VTI1A	vesicle transport through interaction with t-SNAREs 1A	Multiple_Complex
TC0600011213.hg.1	8.07	6.43	3.12	0.0012	0.0987			NonCoding
TC0700013527.hg.1	8.41	6.78	3.09	0.0007	0.0787	SKAP2	src kinase associated phosphoprotein 2	Multiple_Complex
TC0X00007199.hg.1	5.72	4.12	3.04	0.0003	0.0585	WAS	Wiskott-Aldrich syndrome	Multiple_Complex
TC1400008198.hg.1	8.7	7.11	3.01	0.0009	0.0871	MIR342	microRNA 342	Precursor_microRNA
TC2100008160.hg.1	6.46	4.88	2.99	0.0006	0.0741			NonCoding
TC0500013316.hg.1	5.16	3.58	2.97	9.77E-05	0.035	CD180	CD180 molecule	Multiple_Complex
TC0100009395.hg.1	5.31	3.74	2.97	0.0003	0.0585	CD53	Memczak2013 ALT_ACCEPTOR, ALT_DONOR, coding, INTERNAL, intronic best transcript NM_001040033	NonCoding
TC0500007158.hg.1	6.12	4.55	2.97	8.21E-05	0.0319	blawflaw	Transcript Identified by AceView	Unassigned
TC1100011568.hg.1	7.47	5.91	2.95	9.86E-05	0.0352	FCHSD2	FCH and double SH3 domains 2	Multiple_Complex
TC0800007233.hg.1	10.38	8.83	2.94	3.59E-05	0.0207	jerbeyby	Transcript Identified by AceView	Coding

TC0200012527.hg.1	8.09	6.55	2.92	0.0008	0.0826	slarjoybu	Transcript Identified by AceView	Coding
TC1700008049.hg.1	5.47	3.93	2.91	9.90E-06	0.0109	FMNL1	formin like 1	Multiple_Complex
TC1100013014.hg.1	6.2	4.66	2.9	0.0003	0.0598	MS4A7	membrane-spanning 4-domains, subfamily A, member 7	Multiple_Complex
TC1000007883.hg.1	6.6	5.07	2.89	0.0001	0.0359	SRGN	serglycin	Multiple_Complex
TC1000010237.hg.1	8.3	6.77	2.89	0.0003	0.0585	Y_RNA	Y RNA [Source:RFAM;Acc:RF00019]	NonCoding
TC0500013388.hg.1	6.1	4.58	2.87	1.47E-07	0.0011	HAVCR2	hepatitis A virus cellular receptor 2	Multiple_Complex
TC1500010184.hg.1	4.85	3.33	2.87	0.0002	0.0467	BCL2A1	BCL2-related protein A1	Coding
TC1000010523.hg.1	5.81	4.29	2.87	0.0002	0.0436	ANTXRLP1	anthrax toxin receptor-like pseudogene 1	Multiple_Complex
TC0X00009903.hg.1	6.54	5.02	2.85	0.0008	0.0826			NonCoding
TC0500011581.hg.1	5.72	4.21	2.85	0.0006	0.0727	leejor	Transcript Identified by AceView	Coding
TC0900009979.hg.1	6.31	4.81	2.84	0.0012	0.0989			NonCoding
TC1700012191.hg.1	6.29	4.8	2.82	0.0002	0.0452	CD68	CD68 molecule	Coding
TC0400012180.hg.1	6.24	4.75	2.8	1.38E-05	0.0128	RNU6-1285P	RNA, U6 small nuclear 1285, pseudogene [Source:HGNC Symbol;Acc:HGNC:48248]	Small_RNA
TC1100010666.hg.1	9.14	7.66	2.8	0.0006	0.0755			NonCoding
TC0900011305.hg.1	6.31	4.83	2.79	7.68E-06	0.0097	TNC	tenascin C	Multiple_Complex
TC2000009514.hg.1	5.67	4.21	2.75	2.06E-06	0.0048	SUMO1P1	SUMO1 pseudogene 1	Multiple_Complex
TC1900010689.hg.1	4.62	3.16	2.75	5.85E-05	0.0277			NonCoding
TC0X00006448.hg.1	4.83	3.37	2.74	0.0001	0.0383	BX649553.4		Precursor_microRNA
TC0Y00006446.hg.1	4.83	3.37	2.74	0.0001	0.0383	BX649553.4		Precursor_microRNA
TC0100009697.hg.1	6.17	4.71	2.74	6.15E-06	0.0085	FCGR1C	Fc fragment of IgG, high affinity Ic, receptor (CD64), pseudogene	Multiple_Complex
TC1200012565.hg.1	4.83	3.38	2.73	1.13E-05	0.0119			Coding
TC1800006557.hg.1	5.1	3.65	2.72	0.0011	0.0935			NonCoding
TC0700008735.hg.1	5.35	3.91	2.71	0.0002	0.0452			NonCoding
TC1900011898.hg.1	6.64	5.21	2.7	0.0002	0.0472	MIR23A	microRNA 23a	Multiple_Complex
TC0X00010684.hg.1	5.89	4.46	2.69	0.0001	0.0395	CT47A9; CT47A4; CT47A11	cancer/testis antigen family 47, member A9; cancer/testis antigen family 47, member A4; cancer/testis antigen family 47, member A11	Coding
TC0X00010685.hg.1	5.89	4.46	2.69	0.0001	0.0395	CT47A3; CT47A10	cancer/testis antigen family 47, member A3; cancer/testis antigen family 47, member A10	Coding
TC0X00010686.hg.1	5.89	4.46	2.69	0.0001	0.0395	CT47A10; CT47A8; CT47A9	cancer/testis antigen family 47, member A10; cancer/testis antigen family 47, member A8; cancer/testis antigen family 47, member A9	Coding
TC0X00010688.hg.1	5.89	4.46	2.69	0.0001	0.0395	CT47A6; CT47A4	cancer/testis antigen family 47, member A6;	Coding

TC0X00010689.hg.1	5.89	4.46	2.69	0.0001	0.0395	CT47A3	cancer/testis antigen family 47, member A4 cancer/testis antigen family 47, member A3	Coding
TC0X00010690.hg.1	5.89	4.46	2.69	0.0001	0.0395	CT47A2	cancer/testis antigen family 47, member A2	Coding
TC0X00011394.hg.1	5.89	4.46	2.69	0.0001	0.0395	CT47A12; CT47A8	cancer/testis antigen family 47, member A12; cancer/testis antigen family 47, member A8	Coding
TC1000007529.hg.1	7.79	6.36	2.69	0.0013	0.1	katero	Transcript Identified by AceView	Coding
TC0800007231.hg.1	4.73	3.31	2.68	0.0002	0.0446	vyshor	Transcript Identified by AceView	Unassigned
TC0700010940.hg.1	5.83	4.41	2.67	0.0006	0.0769	RP4-647J21.1	novel transcript, overlapping MYO1G	NonCoding
TC0600006544.hg.1	4.33	2.91	2.67	1.35E-06	0.004			NonCoding
TC1200012448.hg.1	6.41	4.99	2.67	0.001	0.0891	soblarbu	Transcript Identified by AceView	Coding
TC1100013152.hg.1	5.11	3.7	2.66	7.93E-05	0.0315	SAA2; SAA2-SAA4; SAA4	serum amyloid A2; SAA2-SAA4 readthrough; serum amyloid A4, constitutive	Multiple_Complex
TC1600006927.hg.1	6.96	5.56	2.65	0.0004	0.0667	snasmey	Transcript Identified by AceView	Unassigned
TC1700007618.hg.1	5.49	4.08	2.65	0.0004	0.0619	AC069363.1	novel transcript, antisense CCL3	NonCoding
TC1600007829.hg.1	12.7	11.29	2.64	0.0003	0.0591	CYLD	cylindromatosis (turban tumor syndrome)	Multiple_Complex
TC1700009378.hg.1	9.53	8.14	2.64	0.0009	0.0857	PITPNA	phosphatidylinositol transfer protein, alpha	Multiple_Complex
TC0600012849.hg.1	6.95	5.55	2.64	0.0005	0.0696	RNU6-906P	RNA, U6 small nuclear 906, pseudogene [Source:HGNC Symbol;Acc:HGNC:47869]	Small_RNA
TC0600007325.hg.1	6.96	5.56	2.63	2.62E-06	0.0054	POM121L6P	POM121 transmembrane nucleoporin-like 6 pseudogene	Multiple_Complex
TC1900009546.hg.1	5.41	4.01	2.63	0.0004	0.0631	PRAM1	PML-RARA regulated adaptor molecule 1	Multiple_Complex
TC1100006995.hg.1	4.97	3.57	2.63	0.0011	0.0945	SAA1	serum amyloid A1	Multiple_Complex
TC0600009535.hg.1	4.77	3.38	2.63	0.0013	0.1	pleekluby	Transcript Identified by AceView	Unassigned
TC0700010941.hg.1	5.53	4.14	2.63	3.86E-05	0.0216	MYO1G	myosin IG	Multiple_Complex
TC1100013120.hg.1	7.25	5.85	2.63	0.0004	0.0612	TCONS_l2_00005353	Salzman2013 ANNOTATED, ncRNA, OVERLAPTX, OVEXON best transcript TCONS_l2_00005353; Salzman2013 ANNOTATED, INTERNAL, ncRNA, OVERLAPTX, OVEXON best transcript TCONS_l2_00005353	NonCoding
TC1800007941.hg.1	5.47	4.08	2.62	0.0009	0.0878	nokey	Transcript Identified by AceView	Coding
TC1400009355.hg.1	7.03	5.64	2.61	0.0004	0.0667	TRMT5	tRNA methyltransferase 5	Multiple_Complex
TC0X00009947.hg.1	6.85	5.46	2.61	0.0002	0.0506			NonCoding

TC0100015856.hg.1	6.29	4.91	2.61	2.79E-06	0.0057	S100A12	S100 calcium binding protein A12	Coding
TC0900011918.hg.1	4.71	3.33	2.6	1.06E-05	0.0114	FCN1	ficolin (collagen/fibrinogen domain containing) 1	Coding
TC0500010763.hg.1	9	7.63	2.59	0.0004	0.0615			NonCoding
TC0200009537.hg.1	5.59	4.23	2.58	1.93E-05	0.0156	KYNU	kynureninase	Multiple_Complex
TC1200011409.hg.1	5.35	3.99	2.57	0.0005	0.0717	slorgly	Transcript Identified by AceView	Coding
TC2200008146.hg.1	4.48	3.12	2.57	0.0001	0.0395	RSPH14	radial spoke head 14 homolog (Chlamydomonas)	Coding
TC0X00006892.hg.1	8.02	6.66	2.57	9.73E-06	0.0108	GK-IT1; GS1-484O17.5	GK intronic transcript 1 (non-protein coding) [Source:HGNC Symbol;Acc:HGNC:41339]; novel transcript; GK intronic transcript 1 [Source:HGNC Symbol;Acc:HGNC:41339]	NonCoding
TC0500011630.hg.1	4.94	3.58	2.56	0.0006	0.0739			NonCoding
TC2200009269.hg.1	5.21	3.85	2.56	4.69E-06	0.0075	APOBEC3A	apolipoprotein B mRNA editing enzyme, catalytic polypeptide-like 3A	Multiple_Complex
TC1700007636.hg.1	5.11	3.75	2.56	0.0001	0.0395	PIGW	phosphatidylinositol glycan anchor biosynthesis class W	Coding
TC1900007222.hg.1	5.33	3.98	2.56	1.22E-05	0.0124	shokaw	Transcript Identified by AceView	Coding
TC1500010650.hg.1	4.79	3.44	2.55	0.0011	0.0952			NonCoding
TC0300010175.hg.1	5.96	4.62	2.54	6.09E-05	0.028	RNU4ATAC17P	RNA, U4atac small nuclear 17, pseudogene [Source:HGNC Symbol;Acc:HGNC:46903]	Small_RNA
TC1900011240.hg.1	6.99	5.64	2.54	0.0009	0.0862	soywee	Transcript Identified by AceView	Coding
TC0200012911.hg.1	5.55	4.2	2.54	0.0004	0.0652	kleekobu	Transcript Identified by AceView	Coding
TC1300009090.hg.1	7.26	5.92	2.53	0.0005	0.0679	flyshorbu	Transcript Identified by AceView	Coding
TC1600007240.hg.1	4.86	3.52	2.52	0.0002	0.051	PRKCB	protein kinase C, beta	Multiple_Complex
TC1200007613.hg.1	5.07	3.74	2.52	0.0004	0.0617	jarchar	Transcript Identified by AceView	Unassigned
TC0700011561.hg.1	4.75	3.42	2.51	2.19E-06	0.005	CCL24	chemokine (C-C motif) ligand 24	Coding
TC1100009202.hg.1	5.41	4.08	2.51	3.91E-06	0.0066	CD3G	CD3g molecule, gamma (CD3-TCR complex)	Multiple_Complex
TC1900011770.hg.1	5.93	4.6	2.51	2.76E-05	0.0185	C5AR1	complement component 5a receptor 1	Multiple_Complex
TC0700006662.hg.1	5.92	4.6	2.51	0.001	0.0931	C1GALT1	core 1 synthase, glycoprotein-N-acetylgalactosamine 3-beta-galactosyltransferase 1	Multiple_Complex
TC1200007255.hg.1	5.08	3.76	2.51	0.0002	0.0477	RP11-50I19.2	novel transcript	NonCoding
TC0500007159.hg.1	6.61	5.29	2.51	0.0006	0.0727	CTD-2353F22.2	TEC	Unassigned
TC1100007777.hg.1	5.18	3.85	2.51	0.0003	0.0552	storglubu	Transcript Identified by AceView	Coding

TC1200009829.hg.1	5.65	4.32	2.5	4.96E-05	0.0252	CLEC4E	C-type lectin domain family 4, member E	Multiple_Complex
TC1600006658.hg.1	7.27	5.95	2.49	2.71E-05	0.0183	IL32	interleukin 32	Multiple_Complex
TC0500007838.hg.1	6.64	5.32	2.49	0.001	0.0888	RNU6ATAC36P	RNA, U6atac small nuclear 36, pseudogene [Source:HGNC Symbol;Acc:HGNC:46935]	Small_RNA
TC0400008443.hg.1	11.08	9.77	2.48	0.0003	0.0585	LARP7	La ribonucleoprotein domain family, member 7	Multiple_Complex
TC1200008874.hg.1	5.49	4.17	2.48	0.0002	0.051	klutybo	Transcript Identified by AceView	Coding
TC1500007930.hg.1	5.81	4.5	2.48	0.0004	0.0672	RP11-797A18.3	novel transcript, antisense to TSPAN3	NonCoding
TC0X00008731.hg.1	7.66	6.35	2.47	0.0005	0.0681	VMA21	VMA21 vacuolar H+-ATPase homolog (S. cerevisiae)	Multiple_Complex
TC1300007596.hg.1	4.55	3.24	2.47	0.0011	0.0962			NonCoding
TC0200008005.hg.1	5.91	4.6	2.47	9.72E-05	0.035	DYSF	dysferlin	Multiple_Complex
TC0100011458.hg.1	5.85	4.54	2.47	8.16E-05	0.0319	TRAF3IP3	TRAF3 interacting protein 3	Multiple_Complex
TC1200008696.hg.1	6.56	5.25	2.47	1.52E-05	0.0139	rekare	Transcript Identified by AceView	Unassigned
TC1000008106.hg.1	7.27	5.97	2.47	0.0005	0.0679	MIR606	microRNA 606	Precursor_microRNA
TC0Y00007217.hg.1	5.14	3.83	2.47	0.0002	0.0489	RP11-473E1.1		Pseudogene
TC0800006476.hg.1	5.26	3.96	2.46	0.0006	0.0755	blybly	Transcript Identified by AceView	Unassigned
TC1300006517.hg.1	5.65	4.35	2.46	0.001	0.0891			NonCoding
TC0X00008370.hg.1	5.99	4.7	2.45	0.0004	0.0619			NonCoding
TC0700006984.hg.1	5.54	4.25	2.45	0.0011	0.0935	SNX10	sorting nexin 10	Multiple_Complex
TC0500011716.hg.1	4.94	3.65	2.45	0.0002	0.0477			NonCoding
TC1000007477.hg.1	4.76	3.47	2.45	0.0007	0.0798	dyklorbu	Transcript Identified by AceView	Unassigned
TC1700012110.hg.1	6.56	5.27	2.44	4.65E-08	0.0005	SECTM1	secreted and transmembrane 1	Multiple_Complex
TC1100010962.hg.1	5.68	4.39	2.44	0.0001	0.0405	MPEG1	macrophage expressed 1	Coding
TC2200006927.hg.1	6.24	4.96	2.44	2.43E-05	0.0171	CTA-390C10.9	novel transcript	NonCoding
TC1600008041.hg.1	5.77	4.48	2.43	0.0003	0.0555	AC010287.1	Uncharacterized protein [Source:UniProtKB/TrEMBL;Acc:A0A0B4J2H1]	Coding
TC0200006468.hg.1	9.96	8.68	2.43	0.0001	0.0372	AC116609.2; AC116609.3	novel transcript	NonCoding
TC0400008301.hg.1	4.78	3.5	2.42	0.0004	0.0619	DDX3P3	DEAD (Asp-Glu-Ala-Asp) box polypeptide 3, pseudogene 3 [Source:HGNC Symbol;Acc:HGNC:42173]	Multiple_Complex
TC0400008786.hg.1	5.26	3.98	2.42	0.0004	0.0664	RNU6-531P	RNA, U6 small nuclear 531, pseudogene [Source:HGNC Symbol;Acc:HGNC:47494]	Small_RNA

TC0100015352.hg.1	7.13	5.85	2.42	8.89E-05	0.0333	NRAS	neuroblastoma RAS viral (v-ras) oncogene homolog	Multiple_Complex
TC1400007277.hg.1	5.88	4.61	2.42	7.92E-05	0.0315	RNU6-1204P	RNA, U6 small nuclear 1204, pseudogene [Source:HGNC Symbol;Acc:HGNC:48167]	Small_RNA
TC0300007521.hg.1	4.72	3.46	2.4	1.92E-05	0.0156			NonCoding
TC1300006911.hg.1	4.61	3.34	2.4	0.0004	0.0629			NonCoding
TC0400010578.hg.1	5.32	4.06	2.4	0.0002	0.0514	YIPF7	Yip1 domain family member 7	Multiple_Complex
TC0500012084.hg.1	5.05	3.79	2.39	0.0004	0.0659	pleyjy	Transcript Identified by AceView	Coding
TC0100010772.hg.1	6.09	4.83	2.39	0.0006	0.074	EIF4A1P11	eukaryotic translation initiation factor 4A1 pseudogene 11 [Source:HGNC Symbol;Acc:HGNC:37932]	Multiple_Complex
TC1000012180.hg.1	4.57	3.32	2.39	8.75E-05	0.0333	blorruby	Transcript Identified by AceView	Coding
TC2200007273.hg.1	5.88	4.63	2.38	7.37E-06	0.0095	CSF2RB	colony stimulating factor 2 receptor, beta, low-affinity (granulocyte-macrophage)	Multiple_Complex
TC0300010756.hg.1	4.09	2.84	2.38	0.0001	0.0359			NonCoding
TC2000008186.hg.1	4.78	3.53	2.37	0.0004	0.0636			NonCoding
TC1600007347.hg.1	6.96	5.72	2.37	3.71E-05	0.021			NonCoding
TC0500009381.hg.1	5.45	4.21	2.36	3.87E-05	0.0216	soyskeeby	Transcript Identified by AceView	Unassigned
TC0500007721.hg.1	6.56	5.33	2.36	0.0005	0.0681	LOC728575	Transcript Identified by AceView, Entrez Gene ID(s) 728575	Coding
TC0200014998.hg.1	6.42	5.18	2.36	2.39E-05	0.0171	cherleybo	Transcript Identified by AceView	Coding
TC1400008710.hg.1	7.71	6.48	2.35	0.0005	0.0699	RNU6-1046P	RNA, U6 small nuclear 1046, pseudogene [Source:HGNC Symbol;Acc:HGNC:48009]	Small_RNA
TC0900011579.hg.1	5.91	4.67	2.35	0.0008	0.0826	huhamu	Transcript Identified by AceView	Unassigned
TC0800009620.hg.1	6.12	4.89	2.35	0.0008	0.0819	RP11-589N15.2	novel transcript, sense intronic to CTSB	NonCoding
TC1200009010.hg.1	5.36	4.14	2.34	0.0002	0.0429	SNRPGP18	small nuclear ribonucleoprotein polypeptide G pseudogene 18 [Source:HGNC Symbol;Acc:HGNC:49374]	Multiple_Complex
TC0900011252.hg.1	5.96	4.74	2.34	0.0002	0.0446	FKBP15	FK506 binding protein 15	Multiple_Complex
TC0100010567.hg.1	5.33	4.1	2.33	0.0003	0.0564			NonCoding
TC1200006658.hg.1	4.14	2.92	2.33	0.001	0.0913	woybleyby	Transcript Identified by AceView	Coding
TC0300010161.hg.1	4.69	3.48	2.32	0.0002	0.047	GRM7-AS3	GRM7 antisense RNA 3	NonCoding
TC0100016656.hg.1	4.69	3.48	2.32	0.0009	0.0885	NCF2	neutrophil cytosolic factor 2	Multiple_Complex
TC0200008951.hg.1	6.68	5.47	2.32	0.0002	0.0424	RNU6-1180P	RNA, U6 small nuclear 1180, pseudogene [Source:HGNC	Small_RNA

							Symbol;Acc:HGNC:48143]	
TC0500007588.hg.1	5.72	4.51	2.32	0.0005	0.0679	RNU6-294P	RNA, U6 small nuclear 294, pseudogene [Source:HGNC Symbol;Acc:HGNC:47257]	Small_RNA
TC1100006742.hg.1	4.93	3.73	2.31	0.0012	0.0964			NonCoding
TC0600007048.hg.1	5.49	4.28	2.31	0.0004	0.0658	bleesneybu	Transcript Identified by AceView	Coding
TC0700006445.hg.1	4.69	3.49	2.3	0.0002	0.0438	FAM20C	family with sequence similarity 20, member C	Multiple_Complex
TC0X00007719.hg.1	5.51	4.31	2.3	0.0004	0.063			NonCoding
TC0400010321.hg.1	4.23	3.03	2.3	0.001	0.0889			NonCoding
TC0600014110.hg.1	5.15	3.95	2.3	0.0002	0.0488	PSMB9	proteasome subunit beta 9	Multiple_Complex
TC0800010106.hg.1	5.66	4.46	2.29	0.0007	0.0787	RP11-89M20.2	novel transcript	NonCoding
TC0600012738.hg.1	5.58	4.39	2.29	0.0009	0.0882			NonCoding
TC1000008401.hg.1	5.48	4.29	2.29	0.0005	0.0717	IFIT5	interferon-induced protein with tetratricopeptide repeats 5	Coding
TC0800006890.hg.1	7.42	6.23	2.28	0.001	0.0917	tawly	Transcript Identified by AceView	Unassigned
TC0100011454.hg.1	4.68	3.49	2.28	2.98E-06	0.0058	HSD11B1	hydroxysteroid (11-beta) dehydrogenase 1	Multiple_Complex
TC1100011127.hg.1	5.78	4.59	2.28	6.34E-05	0.0284	skykoby	Transcript Identified by AceView	Unassigned
TC1400006540.hg.1	4.3	3.11	2.28	0.0003	0.0586	RNASE2	ribonuclease, RNase A family, 2 (liver, eosinophil-derived neurotoxin)	Coding
TC1700010630.hg.1	5.05	3.86	2.28	9.98E-05	0.0353	CCR7	chemokine (C-C motif) receptor 7	Coding
TC0400010548.hg.1	4.76	3.57	2.27	0.0003	0.0585			NonCoding
TC0100008977.hg.1	8.17	6.99	2.27	0.0005	0.0717	GBP1P1	guanylate binding protein 1, interferon-inducible pseudogene 1	Multiple_Complex
TC1600010544.hg.1	4.76	3.58	2.27	0.0012	0.0976			NonCoding
TC1200010703.hg.1	5.15	3.97	2.26	0.0001	0.0359	BIN2	bridging integrator 2	Multiple_Complex
TC0300012936.hg.1	5.05	3.88	2.26	0.0003	0.0564	pusmeyby	Transcript Identified by AceView	Coding
TC1000006803.hg.1	5.55	4.38	2.26	0.0012	0.0977	CAMK1D	Memczak2013 ALT_ACCEPTOR, ALT_DONOR, coding, INTERNAL, intronic best transcript NM_020397	NonCoding
TC0100007677.hg.1	4.12	2.95	2.26	4.84E-05	0.0248			NonCoding
TC0500009227.hg.1	5.87	4.7	2.25	0.0001	0.0418	RNU6-260P	RNA, U6 small nuclear 260, pseudogene [Source:HGNC Symbol;Acc:HGNC:47223]	Small_RNA
TC1700011291.hg.1	4.95	3.78	2.25	0.001	0.0922	LINC01476	long intergenic non-protein coding RNA 1476	NonCoding
TC0100014487.hg.1	7.16	5.99	2.25	0.0001	0.0412	RNU4-88P	RNA, U4 small nuclear 88, pseudogene [Source:HGNC Symbol;Acc:HGNC:47024]	Small_RNA

TC0600008483.hg.1	6.71	5.54	2.25	0.0006	0.0755	RNU6-411P	RNA, U6 small nuclear 411, pseudogene [Source:HGNC Symbol;Acc:HGNC:47374]	Small_RNA
TC0X00010691.hg.1	5.56	4.4	2.25	0.0005	0.0712	CT47A1	cancer/testis antigen family 47, member A1	Coding
TC1900011251.hg.1	6.53	5.37	2.24	0.0004	0.0664	NKG7	natural killer cell granule protein 7	Coding
TC1200012137.hg.1	7.52	6.36	2.24	0.0012	0.0964			NonCoding
TC1700007298.hg.1	4.58	3.42	2.24	2.35E-05	0.0171	MTND2P13	mitochondrially encoded NADH:ubiquinone oxidoreductase core subunit 2 pseudogene 13 [Source:HGNC Symbol;Acc:HGNC:42114]	Multiple_Complex
TC1100007072.hg.1	4.52	3.36	2.23	0.001	0.0931			NonCoding
TC1600009199.hg.1	6.41	5.25	2.23	9.06E-06	0.0104	SLX4	SLX4 structure-specific endonuclease subunit	Multiple_Complex
TC0200014472.hg.1	5.43	4.28	2.22	0.0002	0.0436	RNU6-904P	RNA, U6 small nuclear 904, pseudogene [Source:HGNC Symbol;Acc:HGNC:47867]	Small_RNA
TC0600006442.hg.1	5.66	4.51	2.22	3.93E-07	0.002	IRF4	interferon regulatory factor 4	Multiple_Complex
TC0800009907.hg.1	4.03	2.88	2.22	3.21E-06	0.0059			NonCoding
TC1400008929.hg.1	6.05	4.9	2.22	0.0011	0.0957	PPP2R3C	protein phosphatase 2, regulatory subunit B, gamma	Multiple_Complex
TC0X00007723.hg.1	4.58	3.43	2.22	0.0006	0.0748	slotor	Transcript Identified by AceView	Unassigned
TC1000010027.hg.1	5.27	4.12	2.21	7.20E-05	0.0306	bumeyby	Transcript Identified by AceView	Unassigned
TC0600008538.hg.1	6.48	5.34	2.21	0.0002	0.0444			NonCoding
TC0700007057.hg.1	5.6	4.46	2.2	0.0002	0.051			NonCoding
TC0100014974.hg.1	5.75	4.61	2.2	1.76E-05	0.0153	RP5-837O21.2		Multiple_Complex
TC1000006436.hg.1	6.78	5.64	2.2	0.001	0.0888	narroby	Transcript Identified by AceView	Coding
TC0900011285.hg.1	5.8	4.66	2.2	7.57E-05	0.0309	RP11-9M16.2	novel transcript	NonCoding
TC1400007739.hg.1	5.81	4.67	2.19	9.23E-05	0.0339			NonCoding
TC0100010851.hg.1	5.29	4.15	2.19	0.0004	0.063			NonCoding
TC1400006642.hg.1	5.5	4.36	2.19	0.0007	0.0784	TRAJ13	T cell receptor alpha joining 13	Multiple_Complex
TC2000009722.hg.1	6.44	5.31	2.19	3.24E-05	0.0194			NonCoding
TC1600009185.hg.1	4.72	3.59	2.19	0.0003	0.0564	MEFV	Mediterranean fever	Multiple_Complex
TC0100006881.hg.1	6.06	4.93	2.19	2.23E-05	0.0167	TNFRSF1B; MIR4632; MIR7846	tumor necrosis factor receptor superfamily, member 1B; microRNA 4632; microRNA 7846	Multiple_Complex
TC0200008881.hg.1	7.92	6.8	2.18	0.0001	0.0405			NonCoding
TC0800008116.hg.1	7.5	6.38	2.18	0.0001	0.0396	cheeslybu	Transcript Identified by AceView	Coding
TC1200010683.hg.1	4.61	3.49	2.17	0.0003	0.0521	RP11-407N8.4		Multiple_Complex
TC1700012442.hg.1	8.62	7.5	2.17	0.0004	0.0615	TBC1D3P1-DHX40P1	TBC1D3P1-DHX40P1 readthrough transcribed pseudogene	Multiple_Complex

TC1900009815.hg.1	6.73	5.62	2.16	0.0005	0.0697			NonCoding
TC0X00010373.hg.1	6.18	5.07	2.15	2.33E-05	0.0171	RP1-232L22__B.1		Multiple_Complex
TC0500010349.hg.1	4.7	3.6	2.15	0.0002	0.0458			NonCoding
TC0800007260.hg.1	5.16	4.06	2.15	0.0012	0.0976			NonCoding
TC0700007246.hg.1	7.07	5.96	2.15	0.0004	0.0658	slerblar	Transcript Identified by AceView	Unassigned
TC0X00007176.hg.1	5.11	4.01	2.15	0.0003	0.0517	SSX1	synovial sarcoma, X breakpoint 1	Multiple_Complex
TC0600013467.hg.1	5.31	4.21	2.14	0.0004	0.0659	RP1-69B13.2		Multiple_Complex
TC0600009282.hg.1	8.22	7.12	2.14	0.0002	0.0436	Y_RNA	Y RNA [Source:RFAM;Acc:RF00019]	NonCoding
TC1800008854.hg.1	7.54	6.44	2.14	3.38E-05	0.0199	RP11-879F14.1	novel transcript	NonCoding
TC1100012762.hg.1	4.7	3.61	2.14	5.12E-05	0.0255	KIRREL3	Jeck2013 ALT_ACCEPTOR, ALT_DONOR, coding, INTERNAL, intronic best transcript NM_032531	NonCoding
TC1300008134.hg.1	4.77	3.68	2.13	0.0007	0.0814	smorfa	Transcript Identified by AceView	Coding
TC0X00010175.hg.1	4.58	3.49	2.13	0.001	0.0888			NonCoding
TC1300006987.hg.1	5.54	4.45	2.13	0.0004	0.0667	DGKH	diacylglycerol kinase, eta	Multiple_Complex
TC0500012523.hg.1	6.62	5.53	2.13	0.0009	0.0868	ATOX1	antioxidant 1 copper chaperone	Multiple_Complex
TC1200012844.hg.1	6.47	5.38	2.12	0.0002	0.0486	ARPC3	actin related protein 2/3 complex subunit 3	NonCoding
TC0400007444.hg.1	4.76	3.68	2.12	0.0003	0.0605			NonCoding
TC1600007385.hg.1	5.96	4.89	2.1	8.12E-05	0.0319	nuspar	Transcript Identified by AceView	Coding
TC0100013441.hg.1	6.34	5.27	2.1	0.001	0.0916	FGR	FGR proto-oncogene, Src family tyrosine kinase	Multiple_Complex
TC1600007404.hg.1	4.69	3.62	2.1	6.48E-05	0.0289	LOC606724	Homo sapiens coronin, actin binding protein, 1A pseudogene (LOC606724), non-coding RNA.	Multiple_Complex
TC0400011707.hg.1	4.67	3.6	2.1	0.0004	0.0648	RP11-548H18.2		Multiple_Complex
TC0700009357.hg.1	6.19	5.12	2.09	0.0007	0.0801			NonCoding
TC1000008161.hg.1	7.52	6.45	2.09	0.0008	0.0826			NonCoding
TC0700012399.hg.1	4.27	3.21	2.09	0.0009	0.0873			NonCoding
TC1200012748.hg.1	5.28	4.21	2.09	8.53E-06	0.0102	CD163L1	CD163 molecule-like 1	Multiple_Complex
TC0300012167.hg.1	5.03	3.97	2.09	0.0006	0.0741	doybeebu	Transcript Identified by AceView	Unassigned
TC0200013916.hg.1	4.29	3.23	2.09	0.0011	0.0949	IL1B	interleukin 1 beta	Multiple_Complex
TC0800010939.hg.1	5.68	4.62	2.09	0.0012	0.0972	FTH1P11	ferritin, heavy polypeptide 1 pseudogene 11 [Source:HGNC Symbol;Acc:HGNC:3981]	Pseudogene
TC0200016413.hg.1	5.66	4.6	2.09	0.0003	0.0585	FKBP1B	FK506 binding protein 1B	Multiple_Complex
TC1800009128.hg.1	5.37	4.31	2.09	8.37E-06	0.0102			NonCoding
TC1900011033.hg.1	7.79	6.73	2.08	2.21E-05	0.0167	NAPA	N-ethylmaleimide-sensitive factor	Multiple_Complex

							attachment protein, alpha	
TC1700007261.hg.1	4.87	3.82	2.08	0.0005	0.0696	rawverbu	Transcript Identified by AceView	Multiple_Complex
TC1700011317.hg.1	8.5	7.44	2.08	8.93E-05	0.0333	nymor	Transcript Identified by AceView	Coding
TC0700012449.hg.1	5.4	4.34	2.08	0.0001	0.0418	RNF148	ring finger protein 148	Coding
TC0400008469.hg.1	5.78	4.72	2.08	0.0003	0.0536			NonCoding
TC1900008201.hg.1	6.15	5.09	2.08	0.0005	0.0693	gleyvoy	Transcript Identified by AceView	Unassigned
TC0700013098.hg.1	7.54	6.48	2.07	0.0012	0.0981			NonCoding
TC0200015751.hg.1	5.81	4.76	2.07	0.0008	0.0845	RNU6-642P	RNA, U6 small nuclear 642, pseudogene [Source:HGNC Symbol;Acc:HGNC:47605]	Small_RNA
TC0500007408.hg.1	4.48	3.43	2.07	0.0007	0.0784	GZMK	granzyme K	Multiple_Complex
TC0800010461.hg.1	5.76	4.71	2.07	0.0002	0.0477	keeskoybu	Transcript Identified by AceView	Unassigned
TC0100011645.hg.1	6.02	4.97	2.07	0.0005	0.0679			NonCoding
TC0800007754.hg.1	5.78	4.73	2.07	0.0001	0.0377	SLC2A13P1	SLC2A13 pseudogene 1 [Source:HGNC Symbol;Acc:HGNC:48929]	Multiple_Complex
TC1300007471.hg.1	5.17	4.12	2.07	0.0002	0.0485			NonCoding
TC1900011633.hg.1	6.52	5.48	2.07	0.0004	0.0641	ZBTB45	Memczak2013 ALT_ACCEPTOR, ALT_DONOR, coding, INTERNAL, intronic best transcript NM_032792	NonCoding
TC0800012317.hg.1	4.23	3.19	2.07	0.0004	0.0625			NonCoding
TC1100011233.hg.1	5.83	4.78	2.07	0.0002	0.0429	SNRPGP19	small nuclear ribonucleoprotein polypeptide G pseudogene 19 [Source:HGNC Symbol;Acc:HGNC:49375]	Multiple_Complex
TC0300009218.hg.1	4.88	3.83	2.06	0.0006	0.0736			NonCoding
TC0600009691.hg.1	5.63	4.59	2.06	0.0012	0.0987			Coding
TC1500009584.hg.1	6.31	5.27	2.06	0.0011	0.0937			NonCoding
TC0600011130.hg.1	4.89	3.85	2.06	0.0006	0.0755	HIST1H4C	Jeck2013 ANTISENSE, CDS, coding, INTERNAL, OVCODE, OVEXON, UTR3 best transcript NM_003542	NonCoding
TC0400010791.hg.1	4.74	3.7	2.06	0.0006	0.0727	kekime	Transcript Identified by AceView	Coding
TC1600007417.hg.1	4.85	3.81	2.06	0.0008	0.0845			NonCoding
TC1200007225.hg.1	3.99	2.95	2.06	0.0009	0.0857			NonCoding
TSUnmapped00000757.hg.1	4.31	3.27	2.05	5.57E-05	0.0269	SLC25A26	solute carrier family 25 (S-adenosylmethionine carrier), member 26	Coding
TC1100012771.hg.1	4.39	3.36	2.05	0.0005	0.0679			NonCoding
TC0200014562.hg.1	5.56	4.53	2.05	0.0007	0.077			NonCoding
TC1700007792.hg.1	6.24	5.2	2.05	0.0004	0.0629	slorslu	Transcript Identified by AceView	Coding
TC1600010151.hg.1	5.25	4.22	2.05	8.15E-05	0.0319	RP11-80F22.14	novel transcript	NonCoding

TC0400012862.hg.1	4.93	3.9	2.04	0.0002	0.0482			NonCoding
TC0800006603.hg.1	4.1	3.07	2.04	0.0011	0.0947	DEFB105B; DEFB105A	defensin, beta 105B; defensin, beta 105A	Coding
TC2100007398.hg.1	5.24	4.22	2.03	0.0008	0.0825	LL21NC02-1C16.2	novel transcript	NonCoding
TC2000008281.hg.1	5.33	4.31	2.03	0.0009	0.0859	fato	Transcript Identified by AceView	Coding
TC1100008326.hg.1	4.31	3.29	2.03	0.0001	0.0418	AP000719.1		Precursor_microRNA
TSUnmapped00000476.hg.1	6.29	5.26	2.03	0.0009	0.0872	HMBS	hydroxymethylbilane synthase	NonCoding
TC1000007067.hg.1	4.89	3.87	2.03	1.61E-06	0.0043	MYO3A	myosin IIIA	Multiple_Complex
TC0500013051.hg.1	5.56	4.54	2.03	0.0012	0.0985	seyshuby	Transcript Identified by AceView	Coding
TC0600008851.hg.1	6.33	5.31	2.03	0.0009	0.0859			NonCoding
TC0X00011203.hg.1	6.96	5.94	2.03	0.0003	0.0551	voyvar	Transcript Identified by AceView	Coding
TC1700006950.hg.1	4.44	3.42	2.03	0.0002	0.0511	RP11-214O1.3	novel transcript	NonCoding
TC1100012055.hg.1	5.41	4.39	2.02	0.0012	0.0975			NonCoding
TC2100007105.hg.1	4.92	3.91	2.01	4.19E-05	0.0227			NonCoding
TC0300012166.hg.1	7.35	6.34	2.01	0.0008	0.083	HCLS1	hematopoietic cell-specific Lyn substrate 1	Multiple_Complex
TC1100007867.hg.1	5.17	4.16	2.01	0.0008	0.0828	zaflobu	Transcript Identified by AceView	Unassigned
TC1500008926.hg.1	3.94	2.94	2.01	0.0003	0.0602	RP13-395E19.4		Pseudogene
TC0400010368.hg.1	4.41	3.4	2.01	0.0003	0.0605	faswerbu	Transcript Identified by AceView	Coding
TC1900006997.hg.1	8.45	7.44	2.01	9.56E-05	0.0346	MIR4748	microRNA 4748	Precursor_microRNA
TC0400008099.hg.1	6.12	5.12	2.01	0.0005	0.0679	RNU6-1298P	RNA, U6 small nuclear 1298, pseudogene [Source:HGNC Symbol;Acc:HGNC:48261]	Small_RNA
TC1200012251.hg.1	5.94	4.94	2.01	0.0012	0.0972			NonCoding
TC1100009970.hg.1	5.64	4.63	2	2.91E-05	0.0186	HPX	hemopexin	Multiple_Complex
TC1900010789.hg.1	6.48	5.48	2	2.63E-06	0.0054	POU2F2	POU class 2 homeobox 2	Multiple_Complex
TC0300008625.hg.1	7.53	6.52	2	0.0006	0.0756	Y_RNA	Y RNA [Source:RFAM;Acc:RF00019]	NonCoding
TC1000009533.hg.1	5.35	4.35	2	4.18E-06	0.0069			NonCoding
TSUnmapped00000332.hg.1	5.55	4.55	2	0.0001	0.0419	HHAT	hedgehog acyltransferase	Coding
TC1900008697.hg.1	5.11	4.11	2	0.0011	0.0937	stusee	Transcript Identified by AceView	Coding
TC0X00006447.hg.1	4.04	3.05	1.99	0.0004	0.0634	BX649553.3		Precursor_microRNA
TC0Y00006445.hg.1	4.04	3.05	1.99	0.0004	0.0634	BX649553.3		Precursor_microRNA
TC0100013212.hg.1	3.91	2.92	1.99	0.0003	0.0585			NonCoding
TC0100010560.hg.1	4.98	3.99	1.99	0.0001	0.0357	MIR3119-2	microRNA 3119-2	Precursor_microRNA
TC2100006863.hg.1	4.99	4	1.99	0.0013	0.1	BACH1-IT3; AP000240.7	BACH1 intronic transcript 3 (non-protein coding) [Source:HGNC Symbol;Acc:HGNC:16455]; novel transcript, sense intronic to BACH1; BACH1 intronic transcript 3	NonCoding

							[Source:HGNC Symbol;Acc:HGNC:16455]	
TC0600014236.hg.1	5.53	4.55	1.98	0.0001	0.0412	TXNDC5	thioredoxin domain containing 5 (endoplasmic reticulum)	Multiple_Complex
TC0600007862.hg.1	4.23	3.24	1.98	0.001	0.0908	PIM1	Pim-1 proto-oncogene, serine/threonine kinase	Multiple_Complex
TC0200009786.hg.1	4.78	3.79	1.98	0.0002	0.0517	nudo	Transcript Identified by AceView	Unassigned
TC1400007529.hg.1	6.38	5.4	1.98	0.0004	0.066			NonCoding
TC0200015244.hg.1	7.59	6.6	1.98	0.0006	0.0755	AC067945.3		Pseudogene
TC1400008020.hg.1	6.24	5.25	1.98	5.18E-05	0.0257	RP11-862G15.1; plupar	novel transcript; Transcript Identified by AceView	NonCoding
TC1400009145.hg.1	5.28	4.3	1.98	0.0003	0.0559	fosley	Transcript Identified by AceView	Coding
TC2000009072.hg.1	7.73	6.75	1.98	0.001	0.0911	SNORA71C	small nucleolar RNA, H/ACA box 71C	Small_RNA
TC0600010297.hg.1	4.05	3.07	1.98	0.0004	0.0651			NonCoding
TC0300012168.hg.1	8.38	7.4	1.97	0.0013	0.1	RN7SL172P	RNA, 7SL, cytoplasmic 172, pseudogene [Source:HGNC Symbol;Acc:HGNC:46188]	NonCoding
TC1900009353.hg.1	6.14	5.16	1.97	0.0013	0.1	snarflo	Transcript Identified by AceView	Coding
TC0300007082.hg.1	6.78	5.8	1.97	0.0012	0.0964	RNU6-1227P	RNA, U6 small nuclear 1227, pseudogene [Source:HGNC Symbol;Acc:HGNC:48190]	Small_RNA
TC1700010255.hg.1	7.02	6.04	1.97	0.0012	0.0969	RNU6-920P	RNA, U6 small nuclear 920, pseudogene [Source:HGNC Symbol;Acc:HGNC:47883]	Small_RNA
TC0500008607.hg.1	3.92	2.94	1.97	0.0001	0.0359	korswawbu	Transcript Identified by AceView	Coding
TC0600013609.hg.1	4.24	3.26	1.97	0.0002	0.0452	RP11-475C16.2; snojee	novel transcript; Transcript Identified by AceView	NonCoding
TC0900008437.hg.1	5.64	4.66	1.97	0.001	0.0904			NonCoding
TC0200014987.hg.1	5.01	4.04	1.97	0.0008	0.0831			NonCoding
TC1800008516.hg.1	7.62	6.65	1.97	0.0001	0.0399	RNU6-1242P	RNA, U6 small nuclear 1242, pseudogene [Source:HGNC Symbol;Acc:HGNC:48205]	Small_RNA
TC1200012157.hg.1	6.88	5.9	1.97	7.44E-05	0.0307	RNU6-1088P	RNA, U6 small nuclear 1088, pseudogene [Source:HGNC Symbol;Acc:HGNC:48051]	Small_RNA
TC0400012980.hg.1	5.04	4.06	1.96	0.0007	0.0809	RP11-73G16.3; slykar	novel transcript; Transcript Identified by AceView	Multiple_Complex
TC0X00009102.hg.1	6.16	5.19	1.96	0.0012	0.0969	speetoy	Transcript Identified by AceView	Unassigned
TC0800009514.hg.1	6.76	5.78	1.96	3.66E-05	0.021	CTA-398F10.1; seybeeby	novel transcript; Transcript Identified by AceView	Multiple_Complex
TC1000009775.hg.1	4.38	3.41	1.96	0.0009	0.0857	LINC00710	long intergenic non-protein coding RNA 710	Multiple_Complex

TC0800010365.hg.1	4.61	3.64	1.96	0.0002	0.0476	CTD-2210A23.1		Multiple_Complex
TC1700008104.hg.1	4.77	3.8	1.96	0.0007	0.0785	NSF	N-ethylmaleimide-sensitive factor	Multiple_Complex
TC0800008327.hg.1	6.3	5.33	1.96	0.0003	0.0559	RNU6-703P	RNA, U6 small nuclear 703, pseudogene [Source:HGNC Symbol;Acc:HGNC:47666]	Small_RNA
TC1300007155.hg.1	5.73	4.76	1.96	0.0003	0.0585	stuspey	Transcript Identified by AceView	Coding
TC0100017691.hg.1	4.76	3.79	1.96	0.0002	0.0436	jeerobo	Transcript Identified by AceView	NonCoding
TC1100007049.hg.1	5.01	4.05	1.95	0.0004	0.0669	HTATIP2	HIV-1 Tat interactive protein 2	Multiple_Complex
TC0700009079.hg.1	4.68	3.72	1.95	0.0009	0.086	TSPAN33	tetraspanin 33	Multiple_Complex
TC1800008029.hg.1	4.82	3.86	1.95	0.001	0.0888			NonCoding
TC1300007131.hg.1	5.1	4.14	1.95	0.0006	0.0746			NonCoding
TC0300012588.hg.1	4.82	3.86	1.95	0.001	0.0917			NonCoding
TC1200006862.hg.1	6.06	5.1	1.95	1.21E-05	0.0124	BCL2L14	BCL2-like 14 (apoptosis facilitator)	Multiple_Complex
TC1000007473.hg.1	5.95	4.99	1.95	0.0002	0.0516	ALOX5	Transcript Identified by AceView, Entrez Gene ID(s) 240	Unassigned
TC1500008288.hg.1	7.12	6.16	1.95	0.0009	0.0861	jerder	Transcript Identified by AceView	Unassigned
TC0700010931.hg.1	4.45	3.49	1.95	0.0009	0.086			NonCoding
TC0200013891.hg.1	5.39	4.43	1.95	2.33E-05	0.0171	RP11-803D5.1	TEC	Unassigned
TC0X00006661.hg.1	4.65	3.69	1.94	0.0006	0.0738			NonCoding
TC0100013605.hg.1	5.78	4.82	1.94	0.0008	0.0829	Y_RNA	Y RNA [Source:RFAM;Acc:RF00019]	NonCoding
TC0300009934.hg.1	5	4.04	1.94	0.0007	0.0785	sworsweyby	Transcript Identified by AceView	Coding
TC0600008548.hg.1	4.94	3.99	1.94	0.0003	0.0598			NonCoding
TC0200015656.hg.1	8.66	7.71	1.94	0.0002	0.0514	sleyswyby	Transcript Identified by AceView	Coding
TC1100010785.hg.1	4.9	3.95	1.94	0.0004	0.0628	TRIM53CP	tripartite motif containing 53C, pseudogene [Source:HGNC Symbol;Acc:HGNC:43979]	Multiple_Complex
TC0600007012.hg.1	5.96	5.01	1.94	0.0011	0.0944	CD83	CD83 molecule	Multiple_Complex
TC0100014042.hg.1	5.73	4.77	1.94	0.0005	0.0699			NonCoding
TC1900007173.hg.1	4.94	3.98	1.93	0.0003	0.0585	ADGRE5	adhesion G protein-coupled receptor E5	Multiple_Complex
TC1700010429.hg.1	4.78	3.84	1.93	0.0006	0.0765	RP11-686D22.10		Multiple_Complex
TC0400008793.hg.1	5.02	4.08	1.92	0.0002	0.0489	RN7SL311P	RNA, 7SL, cytoplasmic 311, pseudogene [Source:HGNC Symbol;Acc:HGNC:46327]	NonCoding
TC1800008863.hg.1	5.34	4.4	1.92	0.0005	0.0694	swarvabu	Transcript Identified by AceView	Coding
TC0X00009017.hg.1	6.79	5.85	1.92	0.0001	0.0418	GS1-214D18.3		Pseudogene
TC0600007792.hg.1	6.1	5.16	1.92	4.76E-05	0.0245	PPARD	peroxisome proliferator-activated receptor delta	Multiple_Complex
TC0100012230.hg.1	5.61	4.67	1.92	7.63E-05	0.031			NonCoding

TC0200006931.hg.1	4.96	4.03	1.91	0.0006	0.0754			NonCoding
TC1400008850.hg.1	4.74	3.8	1.91	0.0009	0.0859			NonCoding
TC1100013113.hg.1	4.84	3.91	1.91	0.0004	0.0624			NonCoding
TC0200010800.hg.1	4.77	3.84	1.91	0.0003	0.0582	VIL1	villin 1	Multiple_Complex
TC0600013675.hg.1	3.93	3	1.91	0.0002	0.0467			NonCoding
TC1900010927.hg.1	4.85	3.92	1.91	0.0009	0.0882	starsho	Transcript Identified by AceView	Coding
TC0100016293.hg.1	9.59	8.65	1.91	0.0009	0.0864			NonCoding
TC0300012071.hg.1	4.54	3.61	1.9	0.0002	0.0448			NonCoding
TC1900008465.hg.1	6.57	5.64	1.9	0.0012	0.0967	CTC-453G23.8; CARD8; nekera	Memczak2013 ANTISENSE, CDS, coding, INTERNAL best transcript NM_014959; Transcript Identified by AceView; novel transcript, antisense to CARD8	NonCoding
TC0200011839.hg.1	4.54	3.61	1.9	0.0001	0.0392	swapybo	Transcript Identified by AceView	Coding
TC0100008093.hg.1	5.91	4.98	1.9	0.0001	0.0412	RNU6-369P	RNA, U6 small nuclear 369, pseudogene [Source:HGNC Symbol;Acc:HGNC:47332]	Small_RNA
TC0400010843.hg.1	4.47	3.54	1.9	0.001	0.0888			NonCoding
TC0200011818.hg.1	4.41	3.48	1.9	7.47E-05	0.0307			NonCoding
TC0100015256.hg.1	4.68	3.76	1.9	0.0002	0.0472			NonCoding
TC1200010537.hg.1	3.93	3.01	1.9	0.0002	0.0428			NonCoding
TC0500012529.hg.1	5.89	4.96	1.9	0.0005	0.0679			NonCoding
TC1200007838.hg.1	4.58	3.66	1.9	0.0009	0.0877	IL23A	interleukin 23, alpha subunit p19	Multiple_Complex
TC0800008650.hg.1	5.08	4.16	1.9	0.0011	0.0949	AC023590.1; stola	novel transcript, antisense to SAMD12; Transcript Identified by AceView	NonCoding
TC0100012543.hg.1	4.16	3.23	1.9	8.56E-05	0.0328	FAM213B	Memczak2013 ANTISENSE, coding, INTERNAL, UTR3 best transcript NM_001195737	NonCoding
TC0300009351.hg.1	6.72	5.8	1.89	0.0005	0.0709	SNORA72	Small nucleolar RNA SNORA72 [Source:RFAM;Acc:RF00139]	Small_RNA
TC1500007011.hg.1	5.03	4.11	1.89	0.0002	0.0476	zazaw	Transcript Identified by AceView	Unassigned
TC0100015874.hg.1	7.74	6.82	1.89	0.0003	0.0522			NonCoding
TC1100012851.hg.1	4.63	3.71	1.89	0.0003	0.0604			NonCoding
TC1500010121.hg.1	4.75	3.83	1.89	9.45E-05	0.0344	verjorby	Transcript Identified by AceView	Coding
TC0800008414.hg.1	6.4	5.48	1.89	0.0011	0.0947	NACAP1	nascent-polypeptide-associated complex alpha polypeptide pseudogene 1	Multiple_Complex
TC1600007448.hg.1	5.29	4.37	1.89	0.0007	0.0774	CORO1A	coronin, actin binding protein, 1A	Multiple_Complex
TC0700012614.hg.1	8.48	7.57	1.88	0.0004	0.0667	noykee	Transcript Identified by AceView	Coding
TSUnmapped00000622.hg.1	4.99	4.08	1.88	0.0006	0.0735	GREM1	gremlin 1, DAN family BMP antagonist [Source:HGNC	Coding

							Symbol;Acc:HGNC:2001]	
TC0100013359.hg.1	6.24	5.33	1.88	8.81E-05	0.0333			NonCoding
TC1100010511.hg.1	5.41	4.5	1.88	0.0008	0.0834	RP1-53C18.3		Multiple_Complex
TC0300013796.hg.1	4.69	3.78	1.88	0.0007	0.0809	OXNAD1	oxidoreductase NAD-binding domain containing 1	NonCoding
TC2000007313.hg.1	3.99	3.08	1.88	0.001	0.0891	LBP	lipopolysaccharide binding protein	Coding
TC1900008626.hg.1	4.36	3.45	1.88	3.61E-05	0.0208	SIGLEC19P		Multiple_Complex
TC1400010417.hg.1	5.45	4.54	1.88	0.0008	0.0838			NonCoding
TC0400007830.hg.1	5.42	4.51	1.88	0.0011	0.0962	ALB	albumin	Multiple_Complex
TC0100008377.hg.1	4.66	3.75	1.88	0.0005	0.0717	RP4-758J24.4		Multiple_Complex
TC1600007023.hg.1	6.36	5.45	1.88	0.0002	0.0458	ABCC1	Jeck2013 ALT_ACCEPTOR, ALT_DONOR, coding, INTERNAL, intronic best transcript NM_004996	NonCoding
TC0100009739.hg.1	5.58	4.67	1.88	0.0002	0.0495	stakobo	Transcript Identified by AceView	Coding
TC0600006950.hg.1	5.24	4.33	1.87	0.0006	0.073	TMEM170B	transmembrane protein 170B	Coding
TC1100011190.hg.1	6.79	5.88	1.87	0.0006	0.0746	EHD1	EH domain containing 1	Multiple_Complex
TC0300010478.hg.1	4.82	3.91	1.87	0.0007	0.0779	RNU6-822P	RNA, U6 small nuclear 822, pseudogene [Source:HGNC Symbol;Acc:HGNC:47785]	Small_RNA
TC2100007377.hg.1	5.33	4.43	1.87	0.0003	0.0591	KRTAP10-10	keratin associated protein 10-10	Coding
TC0600007141.hg.1	6.4	5.49	1.87	0.0002	0.0455	E2F3-IT1; flajar; RP1-177P22.1	E2F3 intronic transcript 1 (non-protein coding) [Source:HGNC Symbol;Acc:HGNC:41329]; Transcript Identified by AceView; putative novel transcript; E2F3 intronic transcript 1 [Source:HGNC Symbol;Acc:HGNC:41329]	NonCoding
TC1700007262.hg.1	7.45	6.55	1.87	0.0005	0.0679	MAP2K3	mitogen-activated protein kinase kinase 3	Multiple_Complex
TC1700011106.hg.1	5.46	4.56	1.87	0.0005	0.0697	RP11-94C24.8	novel transcript, antisense to EPN3	NonCoding
TC0X00010504.hg.1	4.01	3.12	1.86	0.0005	0.0703			NonCoding
TC1200011135.hg.1	5.04	4.15	1.86	0.001	0.0917	RBMS1P1	RNA binding motif, single stranded interacting protein 1 pseudogene 1 [Source:HGNC Symbol;Acc:HGNC:9908]	Multiple_Complex
TC0600013204.hg.1	5.25	4.35	1.86	8.74E-05	0.0333	VNN3	vanin 3	Multiple_Complex
TC0900009517.hg.1	4.02	3.12	1.86	0.0008	0.0829	RP11-29B9.2	putative novel transcript	NonCoding
TC1000007665.hg.1	6.47	5.57	1.86	0.0011	0.0942	sershar	Transcript Identified by AceView	Unassigned
TC0400009252.hg.1	5.15	4.25	1.86	0.0011	0.0945			NonCoding

TC0400008648.hg.1	4.21	3.31	1.86	0.0009	0.0857	chergla	Transcript Identified by AceView	Unassigned
TC0400008589.hg.1	5.15	4.25	1.86	0.0002	0.0428	RNU6-550P	RNA, U6 small nuclear 550, pseudogene [Source:HGNC Symbol;Acc:HGNC:47513]	Small_RNA
TC0200009294.hg.1	3.97	3.08	1.86	4.38E-05	0.0232	MTND4P27	mitochondrially encoded NADH:ubiquinone oxidoreductase core subunit 4 pseudogene 27 [Source:HGNC Symbol;Acc:HGNC:42214]	Multiple_Complex
TC0200012038.hg.1	4.73	3.84	1.86	7.71E-05	0.0311	OTOF	otoferlin	Multiple_Complex
TC1100008350.hg.1	5.51	4.62	1.86	0.0001	0.0412			NonCoding
TC1000009794.hg.1	4.14	3.25	1.85	0.0002	0.0455	cherlor	Transcript Identified by AceView	NonCoding
TC1700012375.hg.1	4.59	3.7	1.85	0.0003	0.0585	RP11-19P22.7		Multiple_Complex
TC1000009650.hg.1	4.99	4.1	1.85	0.0007	0.0779			NonCoding
TC1900012038.hg.1	3.97	3.08	1.85	1.34E-05	0.0128	LILRA6	leukocyte immunoglobulin-like receptor, subfamily A (with TM domain), member 6	Multiple_Complex
TC1200006616.hg.1	6.06	5.18	1.85	9.05E-06	0.0104	LTBR	lymphotoxin beta receptor (TNFR superfamily, member 3)	Multiple_Complex
TC0900006999.hg.1	4.96	4.07	1.85	0.0012	0.0988	PTENP1-AS	PTENP1 antisense RNA	Multiple_Complex
TC1200007590.hg.1	4.5	3.61	1.85	0.0002	0.0468	AQP2	aquaporin 2 (collecting duct)	Coding
TC1800006493.hg.1	4.54	3.65	1.85	0.0001	0.038	EMILIN2	elastin microfibril interfacer 2	Multiple_Complex
TC0800009993.hg.1	4.77	3.89	1.85	0.001	0.0888	blawker	Transcript Identified by AceView	Unassigned
TC1500006694.hg.1	6.2	5.31	1.85	0.0005	0.0697			NonCoding
TC0700010171.hg.1	4.55	3.66	1.85	0.0012	0.0964	guchoby	Transcript Identified by AceView	Coding
TC0100012552.hg.1	5.11	4.23	1.85	0.0004	0.0636	byby; RP11-740P5.2	Transcript Identified by AceView; putative novel transcript	NonCoding
TC0100016599.hg.1	4.61	3.72	1.85	4.06E-05	0.0224	smolobu	Transcript Identified by AceView	Coding
TC1200007873.hg.1	6.72	5.84	1.85	0.0008	0.0831	RNU6-879P	RNA, U6 small nuclear 879, pseudogene [Source:HGNC Symbol;Acc:HGNC:47842]	Small_RNA
TC0400009540.hg.1	3.86	2.97	1.84	0.0006	0.0746			NonCoding
TC1900008394.hg.1	5.87	4.99	1.84	0.0004	0.0658	voshaw	Transcript Identified by AceView	Coding
TC1700007334.hg.1	4.35	3.46	1.84	5.77E-05	0.0276	LGALS9DP	lectin, galactoside-binding, soluble, 9D, pseudogene [Source:HGNC Symbol;Acc:HGNC:49896]	Multiple_Complex
TC0800007689.hg.1	4.56	3.67	1.84	5.63E-05	0.0271	RP11-318K15.2	putative novel transcript	NonCoding
TC2000008537.hg.1	3.88	3	1.84	0.0009	0.0882			NonCoding
TC1500009376.hg.1	4.07	3.19	1.84	0.001	0.0888			NonCoding

TC0200009098.hg.1	6.79	5.91	1.84	0.0012	0.0996			NonCoding
TC1100009576.hg.1	12.28	11.4	1.84	0.0012	0.099	zawglee	Transcript Identified by AceView	Coding
TC1900009649.hg.1	5.32	4.44	1.84	0.0004	0.0669			NonCoding
TC0700012042.hg.1	5.93	5.05	1.84	0.0002	0.049	zawshoyby	Transcript Identified by AceView	Coding
TC1800008026.hg.1	5.46	4.58	1.83	9.70E-05	0.035	deyshey	Transcript Identified by AceView	Coding
TC0800010027.hg.1	4.13	3.26	1.83	8.10E-05	0.0319	CTD-3107M8.1		Multiple_Complex
TC1100008595.hg.1	3.99	3.11	1.83	0.0004	0.0667			NonCoding
TC0300008245.hg.1	4.17	3.3	1.83	0.0011	0.0945	ig.217	Transcript Identified by AceView	Coding
TC0100017389.hg.1	3.97	3.1	1.83	0.0004	0.0625	RP11-322F10.2		NonCoding
TC0900010004.hg.1	4.48	3.61	1.83	0.0006	0.0736			NonCoding
TC2100007349.hg.1	5.24	4.37	1.83	0.0006	0.0724	barty	Transcript Identified by AceView	Coding
TC0600009031.hg.1	5.24	4.37	1.83	0.0005	0.0679			NonCoding
TC0900008082.hg.1	6.66	5.79	1.83	6.58E-05	0.0289	RP11-180I4.1		Multiple_Complex
TC0900010073.hg.1	4.87	4	1.82	0.0012	0.0964	RBPJP5	RBPJ pseudogene 5 [Source:HGNC Symbol;Acc:HGNC:37485]	Pseudogene
TC1100010347.hg.1	5.13	4.27	1.82	0.0003	0.0591			NonCoding
TC1600008000.hg.1	5.51	4.64	1.82	0.0002	0.0506	CCL17	chemokine (C-C motif) ligand 17	Coding
TC0200012163.hg.1	5.28	4.42	1.82	0.001	0.0888	XDH	xanthine dehydrogenase	Multiple_Complex
TC1400007945.hg.1	8.16	7.29	1.82	0.0001	0.0415	RP11-750I4.2		Multiple_Complex
TC1600010734.hg.1	7.01	6.14	1.82	0.0012	0.0996	NOB1	NIN1/RPN12 binding protein 1 homolog	Multiple_Complex
TC1100010746.hg.1	4.5	3.64	1.82	0.0008	0.0829	CELF1	Memczak2013 ALT_ACCEPTOR, ALT_DONOR, coding, INTERNAL, intronic best transcript NM_001172639	NonCoding
TC0200013696.hg.1	4.66	3.8	1.82	0.0005	0.0697			NonCoding
TC0100015222.hg.1	4.61	3.75	1.81	0.0003	0.0536			NonCoding
TC1200010203.hg.1	4.6	3.74	1.81	0.0005	0.0679			NonCoding
TC1500006507.hg.1	5.83	4.97	1.81	0.0007	0.0787	GOLGA6L22; GOLGA6L1	golgin A6 family-like 22; golgin A6 family-like 1	Coding
TC1700009795.hg.1	5.09	4.23	1.81	0.0004	0.063	flawwaw	Transcript Identified by AceView	Coding
TC0800012193.hg.1	4.28	3.42	1.81	0.0005	0.0715	feyzarbu; smoyzerbu	Transcript Identified by AceView	Coding
TC1700008749.hg.1	3.96	3.11	1.81	0.0007	0.0776			NonCoding
TC1200011218.hg.1	4.39	3.54	1.81	0.0011	0.0944	RP11-1143G9.4	novel transcript antisense to LYZ	NonCoding
TC1900009712.hg.1	4.15	3.3	1.81	0.0006	0.0727			NonCoding
TC1500006825.hg.1	7.6	6.75	1.81	0.0008	0.0829	morspey	Transcript Identified by AceView	Coding
TC1500006830.hg.1	7.6	6.75	1.81	0.0008	0.0829	beygley	Transcript Identified by AceView	Coding
TC0X00008820.hg.1	5.02	4.17	1.81	0.0006	0.0746	OPN1LW	opsin 1 (cone pigments), long-wave-sensitive	Multiple_Complex
TC0500009565.hg.1	4.85	4	1.8	7.16E-06	0.0093	CTC-251I16.1	TEC	Unassigned

TC0400008757.hg.1	3.77	2.92	1.8	9.91E-05	0.0353			NonCoding
TC2000007480.hg.1	4.38	3.53	1.8	0.001	0.0912			NonCoding
TC0800011404.hg.1	3.93	3.08	1.8	0.0013	0.1	RP11-273P3.1		Multiple_Complex
TC1200007213.hg.1	4.35	3.5	1.8	0.0002	0.0452			NonCoding
TC2100008510.hg.1	4.52	3.68	1.8	0.0012	0.0963	KCNJ15	potassium channel, inwardly rectifying subfamily J, member 15	Multiple_Complex
TC1600010829.hg.1	4.3	3.45	1.8	0.0007	0.0787	plarru	Transcript Identified by AceView	Coding
TC1400009052.hg.1	5.8	4.95	1.8	0.0002	0.045	FSCB	fibrous sheath CABYR binding protein	Coding
TC0800009967.hg.1	4.72	3.88	1.8	0.0001	0.0373	NUGGC	nuclear GTPase, germinal center associated	Coding
TC0200014461.hg.1	4.56	3.71	1.8	0.0007	0.0784			NonCoding
TC1100010164.hg.1	4.4	3.56	1.79	0.0002	0.0472	RP11-23B7.4		Pseudogene
TC2100008423.hg.1	7.2	6.35	1.79	0.0004	0.0628	glyver	Transcript Identified by AceView	Coding
TC0X00006464.hg.1	6.6	5.76	1.79	0.0003	0.0564			NonCoding
TC1300008507.hg.1	4.78	3.94	1.79	0.0008	0.0831			NonCoding
TC0400011862.hg.1	5.16	4.31	1.79	2.89E-05	0.0186	RP11-789C2.1; RP11-1E22.1	novel transcript	NonCoding
TC0400012340.hg.1	5.11	4.27	1.79	0.0009	0.0853			NonCoding
TC0500010302.hg.1	4.27	3.44	1.79	5.98E-05	0.0279	byzaby	Transcript Identified by AceView	Unassigned
TC0400010604.hg.1	5.37	4.53	1.79	0.0006	0.0757	weygy	Transcript Identified by AceView	NonCoding
TC0800011459.hg.1	6.32	5.48	1.79	0.0008	0.0843	RPS17P14	ribosomal protein S17 pseudogene 14	Multiple_Complex
TC1500009894.hg.1	4.93	4.1	1.79	0.0002	0.0453			NonCoding
TC2200008285.hg.1	4.25	3.41	1.78	0.0004	0.0635	CTA-125H2.3		Multiple_Complex
TC0500007608.hg.1	4.71	3.88	1.78	0.0003	0.054	CTD-2353N24.1		Multiple_Complex
TC0X00007407.hg.1	4.92	4.09	1.78	0.0002	0.0508	RP11-445O16.3; stoyber	putative novel transcript; Transcript Identified by AceView	NonCoding
TC1700008206.hg.1	4.69	3.86	1.78	0.0006	0.0735	AC091180.1		Coding
TC0500008555.hg.1	4.47	3.64	1.78	0.001	0.0891	CTD-2195M15.1		Multiple_Complex
TC1000007258.hg.1	5.04	4.21	1.78	0.0002	0.0467	nernoyby	Transcript Identified by AceView	Coding
TC1200011223.hg.1	5.12	4.29	1.78	0.0005	0.0679			NonCoding
TC2100008337.hg.1	6.34	5.51	1.78	0.0011	0.0962			NonCoding
TC1600009735.hg.1	6.62	5.8	1.78	6.09E-05	0.028	sheyzu	Transcript Identified by AceView	Unassigned
TC0800007940.hg.1	6.69	5.86	1.77	0.0002	0.0447	RP11-142A23.1	novel transcript	NonCoding
TC1500006615.hg.1	4.59	3.77	1.77	0.0011	0.0947	flawter	Transcript Identified by AceView	Coding
TC0400012513.hg.1	4.41	3.59	1.77	0.0008	0.0828			NonCoding
TC1100007699.hg.1	5.68	4.85	1.77	0.001	0.0929	CYCSP26	cytochrome c, somatic pseudogene 26	Multiple_Complex
TC1200007147.hg.1	5.52	4.7	1.77	0.0001	0.0359	ARNTL2	aryl hydrocarbon receptor nuclear translocator-like 2	Multiple_Complex
TC0300008971.hg.1	4.43	3.6	1.77	0.0008	0.0826	LOC100507291;	uncharacterized LOC100507291;	NonCoding

						ACTG1P1; RP11-319G6.1	Transcript Identified by AceView, Entrez Gene ID(s) 73; novel transcript, antisense to RBP2, RBP1 & NMNAT3	
TC2200009270.hg.1	3.93	3.11	1.77	0.0001	0.0388	APOBEC3B	apolipoprotein B mRNA editing enzyme, catalytic polypeptide-like 3B	Coding
TC1900009876.hg.1	5.53	4.71	1.77	0.0003	0.0585			NonCoding
TC1400007051.hg.1	4.16	3.34	1.77	0.0008	0.0823			NonCoding
TC0200007035.hg.1	4.09	3.27	1.77	2.87E-05	0.0186	EPT1	ethanolaminephosphotransferase 1	Multiple_Complex
TC0100016006.hg.1	4.66	3.84	1.77	0.0006	0.073	GPATCH4	G-patch domain containing 4	Multiple_Complex
TC1700007620.hg.1	3.76	2.94	1.76	0.0011	0.0962	CCL4L2; CCL4; CCL4L1	chemokine (C-C motif) ligand 4-like 2; chemokine (C-C motif) ligand 4; chemokine (C-C motif) ligand 4-like 1	Multiple_Complex
TC0100009426.hg.1	8.3	7.49	1.76	0.0008	0.0843			NonCoding
TC2100008392.hg.1	3.91	3.1	1.76	0.001	0.091	ITGB2	integrin, beta 2 (complement component 3 receptor 3 and 4 subunit)	Multiple_Complex
TC0800007105.hg.1	7.45	6.64	1.76	0.0004	0.0659	titimu	Transcript Identified by AceView	Coding
TC1100007673.hg.1	5.16	4.34	1.76	0.0002	0.0486	RNA5SP341	5S ribosomal pseudogene 341 [Source:HGNC Symbol;Acc:HGNC:43241]	Ribosomal
TC1000012033.hg.1	4.62	3.81	1.76	4.64E-05	0.0243			NonCoding
TC0900010743.hg.1	4.85	4.04	1.76	0.0011	0.0962	zatoby	Transcript Identified by AceView	Coding
TC1200010044.hg.1	4.28	3.46	1.76	0.0007	0.0769			NonCoding
TC0300009556.hg.1	4.34	3.53	1.76	0.0012	0.0988	RP11-809F4.4		Multiple_Complex
TC2000009363.hg.1	5.03	4.22	1.76	0.0004	0.0655	slorsabu	Transcript Identified by AceView	Coding
TC1100012134.hg.1	3.72	2.91	1.76	0.0005	0.0709	MMP12	matrix metallopeptidase 12	Multiple_Complex
TC0900008219.hg.1	4.79	3.98	1.75	0.0001	0.0381	NR4A3	nuclear receptor subfamily 4, group A, member 3	Multiple_Complex
TC0500009539.hg.1	4.03	3.22	1.75	0.0007	0.0805			NonCoding
TC1400008363.hg.1	6.09	5.28	1.75	0.0009	0.0867	lodoy	Transcript Identified by AceView	Coding
TC1400006554.hg.1	3.89	3.08	1.75	4.99E-05	0.0253	LOC554207; RP11-998D10.4	uncharacterized LOC554207; novel transcript	Multiple_Complex
TC0400010152.hg.1	6.07	5.26	1.75	0.001	0.0891	RP11-141E13.1; hatime	Transcript Identified by AceView; putative novel transcript	Multiple_Complex
TC1200006723.hg.1	4.69	3.88	1.75	0.0006	0.0738			NonCoding
TC0300011343.hg.1	5.1	4.29	1.75	0.0008	0.0842			NonCoding
TC0200012123.hg.1	4.34	3.53	1.75	0.0011	0.0945	AC074011.2	putative novel transcript	NonCoding
TC1300008841.hg.1	5.26	4.46	1.75	0.001	0.0922	storcha	Transcript Identified by AceView	Unassigned
TC0X00006536.hg.1	4.27	3.47	1.75	0.0009	0.0852			NonCoding
TC0300011073.hg.1	4.57	3.77	1.75	0.0002	0.0517			NonCoding

TC0800010520.hg.1	4.25	3.44	1.75	0.0007	0.0792			NonCoding
TC0200013123.hg.1	5.58	4.77	1.74	0.0011	0.094			NonCoding
TC1300009644.hg.1	4.37	3.57	1.74	0.0004	0.0627	LINC00411	long intergenic non-protein coding RNA 411	NonCoding
TC1100012166.hg.1	4.94	4.14	1.74	1.65E-06	0.0043	CASP5	caspase 5	Multiple_Complex
TC1900008613.hg.1	5.01	4.21	1.74	0.0001	0.0409	CTB-147C22.9	novel transcript, antisense to KLK6, KLK7, KLK8 and KLK9	NonCoding
TC0600007908.hg.1	6.67	5.87	1.74	0.0001	0.0412			NonCoding
TC1900010916.hg.1	4.95	4.16	1.74	0.0006	0.0727			NonCoding
TC0100007090.hg.1	6.13	5.33	1.74	0.0004	0.0658	poykeybu	Transcript Identified by AceView	Coding
TC0200011355.hg.1	3.73	2.93	1.74	0.0006	0.0741			NonCoding
TC0200007292.hg.1	4.05	3.25	1.74	0.0009	0.0886			NonCoding
TC2200007204.hg.1	4.11	3.31	1.73	0.0009	0.085	HMOX1	heme oxygenase 1	Multiple_Complex
TC1600006503.hg.1	5.49	4.69	1.73	0.0001	0.0395			NonCoding
TC0300009413.hg.1	6.16	5.37	1.73	0.0004	0.0669	RP11-298O21.6	novel transcript	NonCoding
TC0100008551.hg.1	4.23	3.44	1.73	0.0008	0.0826			NonCoding
TC0800011740.hg.1	5.01	4.22	1.73	0.0004	0.0634			NonCoding
TC0800008504.hg.1	4.8	4.01	1.73	0.0002	0.0488			NonCoding
TC0800007466.hg.1	3.82	3.03	1.73	0.0003	0.0598	POTEA	POTE ankyrin domain family, member A	Multiple_Complex
TC2100007829.hg.1	4.79	4	1.73	0.001	0.0932	EIF4A1P1	eukaryotic translation initiation factor 4A1 pseudogene 1 [Source:HGNC Symbol;Acc:HGNC:3283]	Pseudogene
TC0900012214.hg.1	4.68	3.89	1.73	0.0008	0.0842	IFNA10	interferon, alpha 10	Coding
TC1900011714.hg.1	6.08	5.3	1.73	0.0006	0.0741	LIN37	lin-37 DREAM MuvB core complex component	Multiple_Complex
TC0700013538.hg.1	4.22	3.44	1.72	0.0011	0.0944	TRGJ1; TRGC2; TRGJ2; TRGV9; TRGC1; TRGJP; TRGJP2	T cell receptor gamma joining 1; T cell receptor gamma constant 2; T cell receptor gamma joining 2; T cell receptor gamma variable 9; T cell receptor gamma constant 1; T cell receptor gamma joining P; T cell receptor gamma joining P2	Multiple_Complex
TC0600014176.hg.1	5.57	4.79	1.72	0.0003	0.0573	RP3-486I3.7	novel transcript	NonCoding
TC1700012216.hg.1	4.99	4.2	1.72	0.0011	0.0948	LGALS9	lectin, galactoside-binding, soluble, 9	Multiple_Complex
TC0900011866.hg.1	4.59	3.81	1.72	0.0004	0.0629	gortarby	Transcript Identified by AceView	Coding
TC1000011796.hg.1	5.13	4.35	1.72	0.0011	0.0935			NonCoding
TC0600014234.hg.1	4.48	3.7	1.72	0.0003	0.0604	CAGE1	cancer antigen 1	Multiple_Complex
TC0500009143.hg.1	5.04	4.26	1.72	0.0007	0.0784	nasneebu	Transcript Identified by AceView	Unassigned
TC1900011983.hg.1	4.13	3.35	1.72	0.0003	0.0551	GPR4	G protein-coupled receptor 4	Multiple_Complex
TC0600011843.hg.1	5.32	4.54	1.72	0.0002	0.0488	TBCC	tubulin folding cofactor C	Coding

TC1100008403.hg.1	4.7	3.92	1.71	0.0002	0.0436			NonCoding
TC0300006490.hg.1	4.66	3.88	1.71	0.0005	0.0685	himora	Transcript Identified by AceView	Coding
TC2200007804.hg.1	7.98	7.21	1.71	0.0003	0.0572	RN7SL500P	RNA, 7SL, cytoplasmic 500, pseudogene [Source:HGNC Symbol;Acc:HGNC:46516]	NonCoding
TC0400009278.hg.1	3.51	2.74	1.71	0.0005	0.0679			NonCoding
TC0200011711.hg.1	4.66	3.88	1.71	0.0003	0.0598			NonCoding
TC1200009793.hg.1	4.42	3.65	1.71	0.0012	0.0981	CLEC4C	C-type lectin domain family 4, member C	Coding
TC1500009438.hg.1	4.72	3.95	1.71	0.0011	0.0942	LYSMD2	LysM, putative peptidoglycan-binding, domain containing 2	Coding
TC0700008533.hg.1	9.41	8.63	1.71	0.0006	0.0746			NonCoding
TC0X00010465.hg.1	5.8	5.02	1.71	0.0013	0.1	NUP62CL	nucleoporin 62kDa C-terminal like	Multiple_Complex
TC0500010447.hg.1	6.13	5.36	1.71	0.0007	0.0769	chawjor	Transcript Identified by AceView	Coding
TC0200009801.hg.1	5.48	4.71	1.71	0.0006	0.0727	kawsterby	Transcript Identified by AceView	Coding
TC0600013973.hg.1	4.84	4.07	1.71	0.0003	0.0552			NonCoding
TC0600010089.hg.1	4.89	4.11	1.71	0.0008	0.0832			NonCoding
TC1100008041.hg.1	3.94	3.17	1.71	0.0005	0.0692	CTSW	cathepsin W	Multiple_Complex
TC0X00008140.hg.1	3.93	3.16	1.71	0.0003	0.0526	snudu	Transcript Identified by AceView	Coding
TC1900011490.hg.1	7.81	7.04	1.71	0.0002	0.0476	CTD-2537I9.12	novel transcript, antisense to U2AF2A	NonCoding
TC0900008701.hg.1	3.9	3.13	1.7	0.0001	0.0404			NonCoding
TC0400009211.hg.1	6.02	5.25	1.7	0.0011	0.0935	RP11-366M4.17		Multiple_Complex
TC0500007419.hg.1	4.72	3.96	1.7	0.0005	0.0718			NonCoding
TC0100013635.hg.1	4.79	4.02	1.7	0.001	0.0928			NonCoding
TC1900011860.hg.1	5	4.24	1.7	0.0005	0.0685	DNMT1	DNA (cytosine-5-)-methyltransferase 1	Multiple_Complex
TC1500006819.hg.1	4.42	3.66	1.7	0.0011	0.0935	RP11-1084A12.2; swawpey	novel transcript antisense to SLC12A6; Transcript Identified by AceView	NonCoding
TC0200009592.hg.1	5.38	4.62	1.7	0.0011	0.0947	AC062032.1	novel transcript	NonCoding
TC2000009345.hg.1	4.1	3.34	1.7	0.0011	0.096	RP1-66N13.1	putative novel transcript	NonCoding
TC0900007197.hg.1	6.02	5.25	1.7	0.0003	0.0604			NonCoding
TC0700009544.hg.1	4.2	3.44	1.69	0.0008	0.0832	AC073310.4		Pseudogene
TC0500012740.hg.1	6.68	5.92	1.69	0.0005	0.0679	vuvubo	Transcript Identified by AceView	Coding
TC2100006751.hg.1	5.13	4.37	1.69	0.0003	0.0564			NonCoding
TC1500007928.hg.1	6.21	5.45	1.69	0.0003	0.0585			NonCoding
TC0X00008433.hg.1	4.46	3.7	1.69	7.53E-05	0.0309			NonCoding
TC1500009747.hg.1	4.64	3.88	1.69	0.0012	0.0981			NonCoding
TC0500010137.hg.1	5.15	4.4	1.69	0.0012	0.0976			NonCoding
TC1900008595.hg.1	5.62	4.86	1.69	0.0009	0.0855			NonCoding
TC1900008378.hg.1	3.93	3.18	1.69	0.0006	0.0742			NonCoding
TC0300007340.hg.1	4.71	3.95	1.69	0.0012	0.0985			NonCoding

TC1100012665.hg.1	4.25	3.49	1.69	0.0007	0.0801	OR8C1P	olfactory receptor, family 8, subfamily C, member 1 pseudogene	Pseudogene
TC0900011634.hg.1	4.53	3.78	1.69	0.0005	0.0679	TRUB2	TruB pseudouridine (psi) synthase family member 2	Multiple_Complex
TC2000007006.hg.1	4.59	3.84	1.69	0.0008	0.0843	RNU6-1257P	RNA, U6 small nuclear 1257, pseudogene [Source:HGNC Symbol;Acc:HGNC:48220]	Small_RNA
TC1600006548.hg.1	3.96	3.21	1.69	0.0006	0.0727	TELO2	telomere maintenance 2	Multiple_Complex
TC0600013495.hg.1	4.47	3.71	1.69	0.0009	0.0852	RP11-631F7.1	novel transcript	NonCoding
TC0200007021.hg.1	6.05	5.29	1.68	0.0009	0.086	feyparbo	Transcript Identified by AceView	Coding
TSUnmapped00000493.hg.1	5.91	5.16	1.68	0.0005	0.0679	TRAPPC4	trafficking protein particle complex 4	Coding
TC0100017434.hg.1	6	5.25	1.68	0.0007	0.0801	RP11-378J18.10		Multiple_Complex
TC1200012248.hg.1	4.32	3.57	1.68	0.0011	0.0939	HCAR3	hydroxycarboxylic acid receptor 3	Coding
TC0500010102.hg.1	4.18	3.43	1.68	0.0011	0.0949	CTD-2154B17.3	tec	NonCoding
TC0100010701.hg.1	3.91	3.16	1.68	0.0002	0.046			NonCoding
TC1700011662.hg.1	7.02	6.27	1.68	0.0002	0.0425			NonCoding
TC1200009802.hg.1	4.66	3.91	1.68	0.0008	0.0831	ganee	Transcript Identified by AceView	NonCoding
TC1900009074.hg.1	8.01	7.26	1.68	0.0009	0.0859	sytybu	Transcript Identified by AceView	Coding
TC0500008616.hg.1	4.8	4.05	1.68	0.001	0.0891	poygor	Transcript Identified by AceView	Coding
TC0400010059.hg.1	5.25	4.5	1.68	0.0006	0.0736	RNA5SP156	5S ribosomal pseudogene 156 [Source:HGNC Symbol;Acc:HGNC:43056]	Ribosomal
TC0100011417.hg.1	3.97	3.22	1.68	0.0006	0.0724	CR1L	complement component (3b/4b) receptor 1-like	Multiple_Complex
TC2100007770.hg.1	4.42	3.68	1.68	0.0004	0.0629	LOC101927869; AP000469.2	uncharacterized LOC101927869; novel transcript	NonCoding
TC0300009031.hg.1	4.26	3.51	1.67	0.0008	0.0845	spodobu	Transcript Identified by AceView	Unassigned
TC0100015565.hg.1	4.66	3.91	1.67	0.0006	0.0736	RP11-666A1.4		Multiple_Complex
TC0800008558.hg.1	4.65	3.91	1.67	0.0005	0.0706			NonCoding
TC0900011284.hg.1	4.62	3.88	1.67	0.0005	0.0711	AKNA	AT-hook transcription factor	Multiple_Complex
TC1000007411.hg.1	4.35	3.61	1.67	0.0002	0.0509	RNU6ATAC11P	RNA, U6atac small nuclear 11, pseudogene [Source:HGNC Symbol;Acc:HGNC:46910]	Small_RNA
TC1700010271.hg.1	5.13	4.39	1.67	0.0011	0.0942	RP11-354P11.8; glerror	Transcript Identified by AceView; novel transcript, antisense to NSRP1	Multiple_Complex
TC1600009745.hg.1	6.67	5.93	1.67	0.0009	0.0867	kladu	Transcript Identified by AceView	Unassigned
TC1500010249.hg.1	4.14	3.4	1.67	0.0006	0.0769	FSD2	fibronectin type III and SPRY domain containing 2	Coding

TC0X00007541.hg.1	4.4	3.66	1.67	0.0013	0.1	RNU4-81P	RNA, U4 small nuclear 81, pseudogene [Source:HGNC Symbol;Acc:HGNC:47017]	Small_RNA
TC1700010325.hg.1	5.73	4.99	1.67	0.0007	0.0769			NonCoding
TC0300010419.hg.1	5.39	4.65	1.67	0.0008	0.0826			NonCoding
TC1000011292.hg.1	5.94	5.2	1.67	0.0007	0.0775	byklarbu	Transcript Identified by AceView	Coding
TC1700009690.hg.1	5.44	4.7	1.67	0.0002	0.0429			NonCoding
TC0800007102.hg.1	4.62	3.88	1.67	0.0001	0.0359	PTK2B	protein tyrosine kinase 2 beta	Multiple_Complex
TC1700011874.hg.1	3.93	3.2	1.66	0.0005	0.0709			NonCoding
TC1000011293.hg.1	3.65	2.92	1.66	0.0011	0.0935	byklarbu	Transcript Identified by AceView	Unassigned
TC0200011829.hg.1	5.42	4.69	1.65	0.0008	0.082			NonCoding
TC0200016210.hg.1	3.7	2.98	1.65	0.0007	0.0779	snastarby	Transcript Identified by AceView	Unassigned
TC0500008883.hg.1	6.22	5.49	1.65	0.0001	0.0395	PCDHB12	protocadherin beta 12	Coding
TC1500008827.hg.1	4.87	4.15	1.65	0.0006	0.0754	RP13-126C7.1	novel transcript	NonCoding
TC1000007963.hg.1	4.7	3.98	1.65	0.0008	0.0841	meytarbo	Transcript Identified by AceView	Unassigned
TC0200009196.hg.1	5.52	4.79	1.65	3.06E-05	0.0187			NonCoding
TC1200010169.hg.1	5.29	4.57	1.65	0.0007	0.0785			NonCoding
TC1600011041.hg.1	4.43	3.71	1.65	0.0007	0.0809			NonCoding
TC0300013614.hg.1	4.45	3.73	1.64	0.0012	0.0976	AC090505.4; jeysworby	Transcript Identified by AceView; novel transcript	NonCoding
TC0900010603.hg.1	4.09	3.38	1.64	1.59E-05	0.0143			NonCoding
TC0600013483.hg.1	4.28	3.57	1.64	0.0003	0.0604	jafly	Transcript Identified by AceView	Coding
TC2200007528.hg.1	4.6	3.88	1.64	0.0005	0.0717			NonCoding
TC0500008584.hg.1	5.33	4.62	1.64	0.0009	0.0885			NonCoding
TC0300007783.hg.1	4.9	4.19	1.64	0.0008	0.0828	flaglor	Transcript Identified by AceView	NonCoding
TC0800007959.hg.1	4.07	3.36	1.64	0.0009	0.0859	STAU2-AS1	STAU2 antisense RNA 1	NonCoding
TC1700008566.hg.1	4.34	3.63	1.64	4.50E-05	0.0237	MAP3K3	mitogen-activated protein kinase kinase kinase 3	Multiple_Complex
TC1900011470.hg.1	4.14	3.43	1.63	0.0003	0.0546	UBE2S	ubiquitin-conjugating enzyme E2S	Multiple_Complex
TC1700007459.hg.1	8.02	7.31	1.63	0.0012	0.0985	hutuma	Transcript Identified by AceView	Coding
TC1700006578.hg.1	3.99	3.28	1.63	0.0003	0.0571	OR1A1	olfactory receptor, family 1, subfamily A, member 1	Coding
TC1900011511.hg.1	5.32	4.61	1.63	0.0007	0.0777	ZSCAN5A	zinc finger and SCAN domain containing 5A	NonCoding
TC1000011530.hg.1	4.21	3.5	1.63	0.0004	0.0669			NonCoding
TC0200013660.hg.1	6.07	5.37	1.63	0.0007	0.0801			NonCoding
TC0900011022.hg.1	3.6	2.9	1.63	0.0009	0.0857	pawsarbu	Transcript Identified by AceView	Coding
TC0300011678.hg.1	3.87	3.17	1.63	0.0003	0.0605	slardybo	Transcript Identified by AceView	Coding
TC0900007986.hg.1	4.13	3.42	1.63	0.0009	0.0883			NonCoding
TC1300009613.hg.1	4.53	3.83	1.63	0.0011	0.0949	CLYBL-AS1	CLYBL antisense RNA 1	NonCoding
TC0300013497.hg.1	6.09	5.39	1.63	0.0007	0.0774	RP11-297K7.1		Pseudogene

TC2200007635.hg.1	4.18	3.48	1.62	0.0009	0.0873	watara	Transcript Identified by AceView	Coding
TC0200009799.hg.1	5.83	5.13	1.62	0.0008	0.0834			NonCoding
TC0800012429.hg.1	4.15	3.45	1.62	0.0009	0.086			NonCoding
TC0700006875.hg.1	7.75	7.05	1.62	0.0008	0.0829			NonCoding
TC2000008418.hg.1	3.89	3.2	1.62	0.0003	0.0555	LOC101929486; RP5-1069C8.2	uncharacterized LOC101929486; novel transcript	NonCoding
TC1700007317.hg.1	3.96	3.26	1.62	0.0012	0.0964	RPS16P8	ribosomal protein S16 pseudogene 8	Pseudogene
TC0100017883.hg.1	6.35	5.66	1.62	2.94E-05	0.0186	YWHAQP9	YWHAQ pseudogene 9 [Source:HGNC Symbol;Acc:HGNC:37688]	Multiple_Complex
TC1800007437.hg.1	6.4	5.7	1.62	0.0002	0.0486			NonCoding
TC0100009056.hg.1	4.98	4.29	1.62	0.0011	0.0947	CCDC18	coiled-coil domain containing 18	Multiple_Complex
TC0500012017.hg.1	4.47	3.78	1.62	0.0008	0.0841	IRF1	interferon regulatory factor 1	Multiple_Complex
TC1600010892.hg.1	6.11	5.42	1.62	0.0009	0.0867			NonCoding
TC0300010308.hg.1	5.57	4.88	1.62	0.0007	0.0801			NonCoding
TC1800009167.hg.1	6.06	5.37	1.62	3.54E-05	0.0205	narsaw	Transcript Identified by AceView	NonCoding
TC1000008667.hg.1	7	6.31	1.62	7.72E-05	0.0311			NonCoding
TC0200012941.hg.1	3.75	3.05	1.62	0.0001	0.0396			NonCoding
TC0600007534.hg.1	5.31	4.62	1.62	0.0007	0.0769	PRR3	proline rich 3	Multiple_Complex
TC1900007392.hg.1	5.22	4.53	1.62	0.0002	0.0424	vachey	Transcript Identified by AceView	Coding
TC0900010012.hg.1	4.82	4.13	1.61	0.001	0.0928			NonCoding
TC1000009819.hg.1	4.7	4.01	1.61	0.0006	0.0742	juslu	Transcript Identified by AceView	Coding
TC2100006827.hg.1	3.61	2.92	1.61	0.0005	0.0679			NonCoding
TC1100007914.hg.1	4.58	3.89	1.61	0.0003	0.0585	zumuby	Transcript Identified by AceView	Coding
TC0600010131.hg.1	4.26	3.58	1.61	0.0004	0.0626	derfleeby	Transcript Identified by AceView	Coding
TC1800008775.hg.1	3.84	3.15	1.61	0.001	0.0891			NonCoding
TC0700012957.hg.1	7.44	6.75	1.61	0.0011	0.0935			NonCoding
TC0400007100.hg.1	4.06	3.37	1.61	0.0011	0.0945			NonCoding
TC0100008166.hg.1	5.27	4.58	1.61	0.0003	0.0585	MKNK1-AS1	MKNK1 antisense RNA 1	NonCoding
TC0800011531.hg.1	5.73	5.04	1.61	0.0008	0.0845			NonCoding
TC0600007688.hg.1	3.64	2.95	1.61	0.0013	0.1			NonCoding
TC0600009744.hg.1	5.74	5.05	1.61	0.0007	0.0784	RAB32	RAB32, member RAS oncogene family	Multiple_Complex
TC0900007978.hg.1	4.02	3.33	1.61	0.001	0.0891	latoby	Transcript Identified by AceView	Coding
TC0X00011165.hg.1	3.97	3.29	1.6	0.0008	0.0839	KRT18P48	keratin 18 pseudogene 48 [Source:HGNC Symbol;Acc:HGNC:33418]	Pseudogene
TC1100009314.hg.1	5.95	5.27	1.6	0.0011	0.0957	RNU6-256P	RNA, U6 small nuclear 256, pseudogene [Source:HGNC Symbol;Acc:HGNC:47219]	Small_RNA
TC1700009325.hg.1	6.43	5.75	1.6	0.0002	0.0472			NonCoding
TC1800009172.hg.1	4.04	3.37	1.6	0.0008	0.0843	flawsawbo	Transcript Identified by AceView	Coding

TC1500006617.hg.1	4.03	3.35	1.6	5.14E-05	0.0256			NonCoding
TC0100006696.hg.1	4.83	4.15	1.6	0.0011	0.096	ZBTB48	zinc finger and BTB domain containing 48	Multiple_Complex
TC1900009796.hg.1	3.36	2.69	1.6	0.001	0.0891	skocho	Transcript Identified by AceView	Coding
TC0700007133.hg.1	5.69	5.02	1.6	0.0002	0.045			NonCoding
TC0500007968.hg.1	8.13	7.45	1.6	0.001	0.0908	klusterbu	Transcript Identified by AceView	Coding
TC1700008138.hg.1	4.67	4	1.6	0.0004	0.0624	LRRC46	leucine rich repeat containing 46	Multiple_Complex
TC2000009335.hg.1	3.57	2.89	1.6	0.0004	0.0616			NonCoding
TC0600009710.hg.1	3.63	2.96	1.59	0.0002	0.0457	STX11	syntaxin 11	Coding
TC0300012123.hg.1	5.18	4.51	1.59	0.0006	0.0736	CD80	CD80 molecule	Multiple_Complex
TC1100010742.hg.1	5.17	4.49	1.59	0.0003	0.0564	wofloybu	Transcript Identified by AceView	Unassigned
TC1400009881.hg.1	5.69	5.01	1.59	0.0008	0.0826			NonCoding
TC1900011255.hg.1	4.6	3.93	1.59	0.0008	0.0826	SIGLEC10	sialic acid binding Ig-like lectin 10	Multiple_Complex
TC0500009292.hg.1	4.96	4.29	1.59	7.38E-05	0.0307	CTC-348L5.1	novel transcript	NonCoding
TC1900011731.hg.1	4.97	4.3	1.59	0.0002	0.0452	LGALS17A	Charcot-Leyden crystal protein pseudogene	Multiple_Complex
TC0100008290.hg.1	4.12	3.45	1.59	0.0008	0.0826			NonCoding
TC0900012028.hg.1	4.08	3.41	1.59	0.0002	0.0428			NonCoding
TC1800009095.hg.1	4.53	3.86	1.59	0.0007	0.0769			NonCoding
TC0400012673.hg.1	3.71	3.04	1.59	7.79E-05	0.0312	jergee	Transcript Identified by AceView	NonCoding
TC0900008910.hg.1	5.81	5.14	1.59	0.0002	0.051	C9orf106	chromosome 9 open reading frame 106	Multiple_Complex
TC1600008115.hg.1	3.46	2.8	1.59	0.0008	0.0842			NonCoding
TC1900009872.hg.1	5.44	4.77	1.59	0.0003	0.0533	RASAL3	RAS protein activator like 3	Multiple_Complex
TC0800011872.hg.1	4.87	4.2	1.59	1.85E-05	0.0156	SLA	Src-like-adaptor	Multiple_Complex
TC1500007471.hg.1	5.62	4.96	1.58	0.0006	0.0751			NonCoding
TC0300006915.hg.1	4.21	3.55	1.58	0.0004	0.0667			NonCoding
TC0600012062.hg.1	4.9	4.24	1.58	0.0009	0.0857	heyora	Transcript Identified by AceView	Coding
TC1200009470.hg.1	3.75	3.09	1.58	0.0008	0.0829	RP13-977J11.9	novel transcript	NonCoding
TC1000007980.hg.1	4.4	3.74	1.58	0.0005	0.0679			NonCoding
TC0900007928.hg.1	3.6	2.94	1.58	0.0002	0.0448			NonCoding
TC0X00009723.hg.1	4.11	3.45	1.58	0.0007	0.078	RP11-552J9.11		Multiple_Complex
TC0100014405.hg.1	5.41	4.75	1.58	0.0005	0.0706	RNU6-371P	RNA, U6 small nuclear 371, pseudogene [Source:HGNC Symbol;Acc:HGNC:47334]	Small_RNA
TC1100007763.hg.1	4.61	3.95	1.58	0.0012	0.0996	MS4A3	membrane-spanning 4-domains, subfamily A, member 3 (hematopoietic cell-specific)	Multiple_Complex
TC0300012388.hg.1	5.27	4.61	1.58	0.0004	0.0627			NonCoding
TC2000006690.hg.1	5.89	5.23	1.58	0.001	0.0911			NonCoding
TC0200006871.hg.1	5.29	4.63	1.58	0.0012	0.0988			NonCoding
TC1300007546.hg.1	4.05	3.4	1.57	0.0005	0.0699	IRG1	immunoresponsive 1 homolog (mouse)	Coding

TC0700010028.hg.1	5.91	5.26	1.57	0.0004	0.0667	LOC401296 ; AC074389. 6	uncharacterized LOC401296; novel transcript	Multiple_Complex
TC0800006446.hg.1	4.33	3.68	1.57	0.0006	0.0756			NonCoding
TC1600009274.hg.1	4.26	3.6	1.57	0.0002	0.0504			NonCoding
TC0100011441.hg.1	4.76	4.11	1.57	0.0012	0.0966	RP11-385M4.2		Multiple_Complex
TC0100016770.hg.1	5.99	5.33	1.57	9.06E-06	0.0104			NonCoding
TC1900006833.hg.1	4.54	3.89	1.57	0.0006	0.0762	MBD3L5	methyl-CpG binding domain protein 3-like 5	Coding
TC0X00008397.hg.1	6.46	5.81	1.57	0.0008	0.082	shana	Transcript Identified by AceView	Coding
TC0600009259.hg.1	3.86	3.21	1.56	0.0008	0.0845			NonCoding
TC0200012918.hg.1	5.48	4.83	1.56	0.0005	0.0709	pleejorbo	Transcript Identified by AceView	Coding
TC0300008569.hg.1	3.84	3.2	1.56	0.0002	0.0506			NonCoding
TC0700012222.hg.1	4.65	4	1.56	0.0007	0.0787			NonCoding
TC0X00006451.hg.1	4.09	3.44	1.56	0.0006	0.0741	BX649553. 1		Precursor_microRNA
TC0Y00006449.hg.1	4.09	3.44	1.56	0.0006	0.0741	BX649553. 1		Precursor_microRNA
TC0200016415.hg.1	4.52	3.87	1.56	0.0009	0.0882	FAM228A	family with sequence similarity 228, member A	Coding
TSUnmapped00000481.hg.1	6.72	6.07	1.56	0.0009	0.0856	DUSP16	dual specificity phosphatase 16	NonCoding
TC1900007785.hg.1	3.39	2.75	1.56	0.0006	0.0727	flartor	Transcript Identified by AceView	Coding
TC1900009594.hg.1	6.43	5.79	1.56	0.0006	0.076	ZNF812P	zinc finger protein 812, pseudogene	Multiple_Complex
TC0500007306.hg.1	3.83	3.19	1.56	0.0007	0.0794	RP11-8L21.1	putative novel transcript	NonCoding
TC0500007237.hg.1	5.86	5.22	1.56	0.0008	0.0825	CARD6	caspase recruitment domain family, member 6	Multiple_Complex
TC1300008513.hg.1	4.28	3.64	1.56	0.0004	0.0624	LINC00426	long intergenic non-protein coding RNA 426	NonCoding
TC0900010601.hg.1	4.26	3.63	1.56	0.0002	0.0476			NonCoding
TC0100013984.hg.1	3.58	2.94	1.56	0.0003	0.0585			NonCoding
TC1900008169.hg.1	6.48	5.84	1.56	0.0004	0.0671			NonCoding
TC0500012054.hg.1	4.07	3.43	1.56	0.0003	0.0538			NonCoding
TC0200010293.hg.1	4.65	4.01	1.55	0.0012	0.0963			NonCoding
TC1700011907.hg.1	3.32	2.69	1.55	0.0007	0.0801	LOC101928710; CTD-2357A8.3; steetee	uncharacterized LOC101928710; novel transcript; Transcript Identified by AceView	NonCoding
TC0200010980.hg.1	4.27	3.64	1.55	0.0008	0.0829	CCL20	chemokine (C-C motif) ligand 20	Multiple_Complex
TC0600014038.hg.1	6.58	5.95	1.55	0.0012	0.0971			NonCoding
TC1800006992.hg.1	3.76	3.13	1.55	0.0005	0.0705	MIR302F	microRNA 302f	Multiple_Complex
TC1000008011.hg.1	4.27	3.64	1.55	0.0003	0.055	OIT3	oncoprotein induced transcript 3	Multiple_Complex
TC1900008609.hg.1	4.29	3.66	1.55	0.0001	0.0377			NonCoding
TC0900008717.hg.1	5.95	5.32	1.55	0.0011	0.0949	blersyby; RP11-121A14.3	Transcript Identified by AceView; putative novel transcript	NonCoding
TC0200010822.hg.1	5.63	5	1.55	0.0005	0.0685	storgarbu	Transcript Identified by AceView	Coding

TC0100013190.hg.1	6.27	5.64	1.54	0.0007	0.0774	RP5-930J4.5		Multiple_Complex
TC0200008115.hg.1	5.07	4.45	1.54	0.0004	0.0667	skerjoybo	Transcript Identified by AceView	Coding
TC1200009675.hg.1	4.44	3.81	1.54	0.0007	0.0775	RP11-234B24.5		Pseudogene
TC0900011170.hg.1	5.56	4.94	1.54	9.56E-05	0.0346	blosawbu	Transcript Identified by AceView	Coding
TC1200007436.hg.1	4.21	3.58	1.54	0.0005	0.0709			NonCoding
TC1300007256.hg.1	4	3.37	1.54	0.0002	0.0436	OLFM4	olfactomedin 4	Coding
TC0600009986.hg.1	4.2	3.58	1.54	0.0005	0.0679	MIR3692	microRNA 3692	Precursor_microRNA
TC0100018407.hg.1	4.54	3.92	1.54	0.0005	0.0679	PRAMEF6; PRAMEF5	PRAME family member 6; PRAME family member 5	Coding
TC0200007747.hg.1	9.89	9.27	1.54	0.0005	0.0717	MIR5192	microRNA 5192	Precursor_microRNA
TC0600013156.hg.1	3.73	3.11	1.53	4.21E-05	0.0227			NonCoding
TC1500009192.hg.1	4.68	4.06	1.53	0.0009	0.0859	LRRC57	leucine rich repeat containing 57	Multiple_Complex
TC1100008381.hg.1	4.94	4.32	1.53	0.0006	0.0727	P2RY6	pyrimidinergic receptor P2Y, G-protein coupled, 6	Coding
TC2200008657.hg.1	7.77	7.16	1.53	0.0012	0.0964			NonCoding
TC1900012004.hg.1	5.76	5.15	1.53	0.0009	0.0852	PTOV1-AS2	PTOV1 antisense RNA 2	NonCoding
TC0600013638.hg.1	4.71	4.1	1.53	6.84E-05	0.0296			NonCoding
TC0X00008957.hg.1	5.14	4.53	1.53	0.0008	0.0826	dorfo	Transcript Identified by AceView	Coding
TC0X00008962.hg.1	5.14	4.53	1.53	0.0008	0.0826	jarjo	Transcript Identified by AceView	Coding
TC0200015469.hg.1	7.96	7.35	1.53	0.0008	0.0838			NonCoding
TC1900007268.hg.1	8.53	7.92	1.52	0.001	0.0929	AP1M1	Memczak2013 ALT_ACCEPTOR, ALT_DONOR, coding, INTERNAL, intronic best transcript NM_032493	NonCoding
TC0600012756.hg.1	3.69	3.09	1.52	0.0009	0.0862			NonCoding
TC1400008167.hg.1	5.45	4.84	1.52	0.0001	0.0383	AL109767.1	putative novel transcript	NonCoding
TC2000007533.hg.1	5.19	4.58	1.52	0.0004	0.063			NonCoding
TC0900008997.hg.1	6.39	5.79	1.52	0.0005	0.0679			NonCoding
TC1900007538.hg.1	3.87	3.27	1.52	0.0001	0.0372			NonCoding
TC1300008962.hg.1	5.09	4.5	1.51	0.001	0.0891			NonCoding
TC0200006469.hg.1	11.06	10.46	1.51	0.0012	0.0981			NonCoding
TC0100012609.hg.1	3.88	3.28	1.51	0.0003	0.054	RP13-614K11.2	putative novel transcript	NonCoding
TC2200007264.hg.1	3.76	3.16	1.51	0.0012	0.0972			NonCoding
TC0200009103.hg.1	5.55	4.96	1.51	0.0007	0.0778			NonCoding
TC2000007125.hg.1	4.61	4.02	1.51	0.0005	0.071	LOC149950; RP11-410N8.4	uncharacterized LOC149950; novel transcript	Multiple_Complex
TC0X00009328.hg.1	5.92	5.33	1.51	6.84E-05	0.0296	CXorf21	chromosome X open reading frame 21	Coding
TC0200013689.hg.1	4.99	4.4	1.51	0.0009	0.0863			NonCoding
TC0700012612.hg.1	5.11	4.52	1.5	0.0011	0.0947	AC016831.6		Multiple_Complex
TC0100012568.hg.1	4.05	3.47	1.5	0.0009	0.086			NonCoding
TC0100017438.hg.1	4.51	3.92	1.5	0.0012	0.0981	RP11-378J18.6		Pseudogene

TC2200006683.hg.1	4.83	4.24	1.5	0.0006	0.0769	LOC101928891; XXbac-B135H6.15	uncharacterized LOC101928891; putative novel transcript	NonCoding
TC1600009395.hg.1	3.72	3.13	1.5	7.44E-05	0.0307	SOCS1	suppressor of cytokine signaling 1	Coding
TC0600010982.hg.1	2.81	3.4	-1.5	0.0009	0.0871	RP3-471C18.2; RP3-471C18.1; shawjar	putative novel transcript; novel transcript; Transcript Identified by AceView	NonCoding
TC0900008297.hg.1	3.02	3.62	-1.52	0.0012	0.0977	NIPSNAP3A; NIPSNAP3B	nipsnap homolog 3A (C. elegans); nipsnap homolog 3B (C. elegans)	Multiple_Complex
TC1900011943.hg.1	6.17	6.8	-1.55	0.0007	0.0805	CTC-360G5.9	novel transcript	NonCoding
TC0900010155.hg.1	6	6.63	-1.55	0.0007	0.0779	LOC103908605; RP11-475I24.3	uncharacterized LOC103908605; novel transcript	NonCoding
TC0400007134.hg.1	2.84	3.48	-1.55	0.0012	0.0985			NonCoding
TC1500007004.hg.1	5.05	5.69	-1.56	0.0009	0.0859	RTF1	RTF1 homolog, Paf1/RNA polymerase II complex component	Multiple_Complex
TC0Y00006692.hg.1	3.98	4.63	-1.57	0.0008	0.0845	FAM224B	family with sequence similarity 224, member B (non-protein coding)	NonCoding
TC0100013285.hg.1	4.35	5	-1.57	0.0001	0.0409	plerkler	Transcript Identified by AceView	Multiple_Complex
TC1600008531.hg.1	5.18	5.84	-1.58	0.0008	0.0834	RP11-319G9.1	TEC	Unassigned
TC1000011807.hg.1	6	6.67	-1.59	0.0006	0.0727	SMNDC1	survival motor neuron domain containing 1	Multiple_Complex
TC0900008949.hg.1	4.61	5.28	-1.59	0.0013	0.1	NCS1	neuronal calcium sensor 1	Multiple_Complex
TC0900011035.hg.1	4.79	5.47	-1.61	0.0002	0.0425	TMEM246	transmembrane protein 246	Multiple_Complex
TC1500007903.hg.1	4.08	4.77	-1.61	0.0011	0.0947	CTD-2026K11.6	novel transcript, antisense to CSPG4	NonCoding
TC1200011715.hg.1	4.21	4.9	-1.61	0.0004	0.0628	HSPE1P4	heat shock protein family E (Hsp10) member 1 pseudogene 4 [Source:HGNC Symbol;Acc:HGNC:49323]	Multiple_Complex
TC0600009971.hg.1	6.06	6.75	-1.62	0.0007	0.0789	fustyby	Transcript Identified by AceView	Coding
TC1500008297.hg.1	3.36	4.05	-1.62	0.0008	0.0828			NonCoding
TC0200010387.hg.1	3.6	4.31	-1.64	0.0002	0.0458	stykly	Transcript Identified by AceView	Unassigned
TC1400007206.hg.1	3.01	3.72	-1.64	0.0008	0.0826			NonCoding
TC22_KI270733v1_random00006435.hg.1	13.37	14.1	-1.65	0.001	0.0902	RNA5-8S5	RNA, 5.8S ribosomal 5	Multiple_Complex
TC1100010240.hg.1	4.13	4.86	-1.67	0.0001	0.0377	SDHCP4	succinate dehydrogenase complex subunit C pseudogene 4 [Source:HGNC Symbol;Acc:HGNC:45179]	Multiple_Complex
TC1700010332.hg.1	3.86	4.6	-1.67	0.0002	0.0429	RP11-640N20.5		Multiple_Complex
TC1000007767.hg.1	7.26	8	-1.67	0.0004	0.0617	AL671972.1		Precursor_microRNA
TC0900008865.hg.1	4.37	5.12	-1.69	0.0006	0.0769	CERCAM	cerebral endothelial cell adhesion molecule	Multiple_Complex

TC1500007626.hg.1	4.71	5.47	-1.69	0.001	0.0896	RP11-622C24.2	novel transcript	NonCoding
TC0400012898.hg.1	4.95	5.71	-1.69	0.0007	0.0775			NonCoding
TC1800006597.hg.1	5.87	6.64	-1.7	0.0011	0.0935	shakler	Transcript Identified by AceView	Coding
TC1300008926.hg.1	4.13	4.9	-1.7	0.0013	0.1	KPNA3	karyopherin alpha 3 (importin alpha 4)	Multiple_Complex
TC0500007132.hg.1	3.8	4.58	-1.71	0.0009	0.0871	AC027347.1		Precursor_microRNA
TC1700008029.hg.1	9.16	9.95	-1.73	0.0005	0.0679			NonCoding
TC0300009673.hg.1	6.84	7.63	-1.73	0.0012	0.0996	KLHL24	kelch-like family member 24	Multiple_Complex
TC1800008230.hg.1	3.77	4.56	-1.73	0.0013	0.1			NonCoding
TC0100017550.hg.1	2.86	3.65	-1.73	0.0006	0.0738	verroybu	Transcript Identified by AceView	Coding
TC0400012793.hg.1	5.23	6.03	-1.74	0.0001	0.0384	jorsmarby; snoflor	Transcript Identified by AceView	NonCoding
TC0800007200.hg.1	5.97	6.77	-1.75	0.0002	0.0429			NonCoding
TC1400006840.hg.1	3.19	4	-1.75	0.0012	0.0964			NonCoding
TC0100010583.hg.1	3.97	4.79	-1.76	0.0002	0.0508	FMO2	flavin containing monooxygenase 2	Multiple_Complex
TC0700012479.hg.1	4.4	5.22	-1.77	0.0011	0.0942	POT1	protection of telomeres 1	Multiple_Complex
TC0200009397.hg.1	6.93	7.75	-1.77	0.0004	0.0641	RN7SKP93	RNA, 7SK small nuclear pseudogene 93 [Source:HGNC Symbol;Acc:HGNC:45817]	NonCoding
TC1200011299.hg.1	3.21	4.04	-1.78	0.0012	0.0985	RP11-585P4.6	novel trancsript, sense intronic to CAPS2	NonCoding
TC1300006727.hg.1	4.09	4.93	-1.79	0.0007	0.0808	Y_RNA	Y RNA [Source:RFAM;Acc:RF00019]	NonCoding
TC0900010082.hg.1	4.67	5.52	-1.8	0.0003	0.0598	AL772307.1		Multiple_Complex
TC1200007156.hg.1	5.11	5.97	-1.81	0.0009	0.0882	starguby	Transcript Identified by AceView	Unassigned
TC1400009040.hg.1	3.99	4.85	-1.81	0.0003	0.0551	snarcheebu	Transcript Identified by AceView	Unassigned
TC1100008904.hg.1	5.29	6.15	-1.81	0.0002	0.0436	YAP1	Yes-associated protein 1	Multiple_Complex
TC0700013567.hg.1	4.54	5.4	-1.82	0.0006	0.0738	LINC00174	long intergenic non-protein coding RNA 174	NonCoding
TC0300010221.hg.1	3.48	4.34	-1.82	0.0007	0.0808	spuswoybu	Transcript Identified by AceView	NonCoding
TC1600009091.hg.1	4.26	5.12	-1.82	0.001	0.0888	AC009065.3		Precursor_microRNA
TC0100015894.hg.1	6.99	7.86	-1.82	0.0004	0.0669	JTB	jumping translocation breakpoint	Multiple_Complex
TC0X00011317.hg.1	7.03	7.9	-1.82	0.0003	0.0571	TCEAL1	transcription elongation factor A (SII)-like 1	Multiple_Complex
TC1200009522.hg.1	3.24	4.1	-1.82	0.0003	0.0585	lubloybu	Transcript Identified by AceView	Coding
TC0900010353.hg.1	6.92	7.8	-1.84	0.0006	0.0765	poytawby	Transcript Identified by AceView	Unassigned
TC0900010142.hg.1	4.9	5.78	-1.84	0.0009	0.0877	Y_RNA	Y RNA [Source:RFAM;Acc:RF00019]	NonCoding
TC0X00007524.hg.1	6.31	7.19	-1.84	0.0009	0.086	RNA5SP506	5S ribosomal pseudogene 506 [Source:HGNC Symbol;Acc:HGNC:43406]	Ribosomal
TC0100014737.hg.1	3.68	4.57	-1.85	0.0006	0.076	furoybo	Transcript Identified by AceView	Unassigned

TC2200008586.hg.1	8.64	9.53	-1.85	0.0009	0.0877			NonCoding
TC2200008389.hg.1	4.19	5.09	-1.86	5.05E-05	0.0254			NonCoding
TC0100013119.hg.1	7.78	8.68	-1.86	0.0013	0.1	MIR1290	microRNA 1290	Precursor_ microRNA
TC0200009009.hg.1	4.74	5.63	-1.86	0.0004	0.0617	skajybo	Transcript Identified by AceView	Coding
TC0200008138.hg.1	9.56	10.46	-1.86	0.0002	0.0425			NonCoding
TC1200011591.hg.1	4.5	5.4	-1.86	0.0009	0.0868	NTN4	netrin 4	Multiple_Co mplex
TC1900007697.hg.1	3.8	4.7	-1.87	0.0004	0.0641	TAF9P3	TAF9 RNA polymerase II, TATA box binding protein (TBP)-associated factor, 32kDa pseudogene 3	Multiple_Co mplex
TC1500007751.hg.1	3.94	4.85	-1.88	0.0004	0.0651	worrey	Transcript Identified by AceView	NonCoding
TC0100006975.hg.1	3.33	4.24	-1.88	0.0011	0.0949	beymorbu	Transcript Identified by AceView	Coding
TC0300006847.hg.1	3.09	4.01	-1.89	0.0012	0.0998	NR1D2	nuclear receptor subfamily 1, group D, member 2	Multiple_Co mplex
TC0700007814.hg.1	4.55	5.47	-1.89	4.70E-05	0.0245	SNORA22	small nucleolar RNA, H/ACA box 22	Small_RNA
TC1000010177.hg.1	4.75	5.67	-1.89	0.0007	0.0785			NonCoding
TC0400010593.hg.1	8.09	9.02	-1.9	0.001	0.0891			NonCoding
TC0900008173.hg.1	5.1	6.03	-1.9	0.0002	0.0444	ANP32B	acidic nuclear phosphoprotein 32 family member B	Multiple_Co mplex
TC0900012011.hg.1	5.87	6.8	-1.91	0.0005	0.0718	EDF1	endothelial differentiation-related factor 1	Multiple_Co mplex
TC1000007493.hg.1	3.8	4.73	-1.91	0.0008	0.0826	SNORA74	Small nucleolar RNA SNORA74 [Source:RFAM;Acc:RF 00090]	Small_RNA
TC0200009957.hg.1	4.7	5.63	-1.91	0.0008	0.0828			NonCoding
TC0900010121.hg.1	3.87	4.81	-1.91	0.0004	0.0631	kerskubu; leeworby; neyskubu; plaworby	Transcript Identified by AceView	Multiple_Co mplex
TC0900008272.hg.1	4.86	5.8	-1.92	0.0003	0.0533	AL391867.1		Precursor_ microRNA
TC0100008851.hg.1	3.32	4.27	-1.92	0.0008	0.0828			NonCoding
TC0300010174.hg.1	3.67	4.62	-1.92	0.0003	0.0538	nospoby	Transcript Identified by AceView	Coding
TC0500012293.hg.1	4.76	5.7	-1.93	0.0003	0.0536			NonCoding
TC1700010540.hg.1	4.46	5.41	-1.94	0.0005	0.0706	PCGF2	polycomb group ring finger 2	Coding
TC0700008623.hg.1	5.62	6.58	-1.94	0.0005	0.0696	CTA-313A17.3		Pseudogen e
TC1500006677.hg.1	5.34	6.3	-1.94	0.0009	0.0869	pluchubu	Transcript Identified by AceView	Coding
TC0200010085.hg.1	4.2	5.16	-1.95	0.0008	0.0843			NonCoding
TC0800011683.hg.1	4.23	5.2	-1.96	0.0008	0.082	FBXO32	F-box protein 32	Multiple_Co mplex
TC0100011360.hg.1	4.27	5.24	-1.97	0.0008	0.082	BX571818.1		Precursor_ microRNA
TC2100008394.hg.1	4.35	5.33	-1.97	0.0011	0.0949	LL21NC02-1C16.1	novel transcript, sense intronic to ITGB2	NonCoding
TC0800011675.hg.1	4.06	5.04	-1.97	0.0001	0.0396	MIR4663	microRNA 4663	Precursor_ microRNA
TC0200014740.hg.1	5.4	6.38	-1.98	0.0012	0.098			NonCoding
TC0500007238.hg.1	2.75	3.74	-1.99	0.0004	0.0621	C7	complement component 7	Multiple_Co mplex

TC0800010289.hg.1	4.81	5.81	-2	2.44E-05	0.0171			NonCoding
TC1800007505.hg.1	3.85	4.85	-2	0.001	0.0902	RP11-173A16.2	novel transcript, sense intronic to KIAA1468	NonCoding
TC1100012542.hg.1	4.51	5.51	-2.01	0.0008	0.0829	RP11-831A10.2; shumey	novel transcript; Transcript Identified by AceView	NonCoding
TC0800009503.hg.1	5.65	6.66	-2.01	0.0007	0.0798	glowor	Transcript Identified by AceView	Unassigned
TC0500007689.hg.1	3.34	4.35	-2.02	0.0001	0.0418			NonCoding
TC0300010057.hg.1	3.66	4.68	-2.03	0.0012	0.0964	plunawbo	Transcript Identified by AceView	Coding
TC0500010135.hg.1	4.41	5.43	-2.03	0.0002	0.0429	gerskeybu	Transcript Identified by AceView	Coding
TC0700011984.hg.1	7.44	8.46	-2.03	0.0004	0.0669	AC005071.3		Precursor_microRNA
TC0200007596.hg.1	5.76	6.78	-2.03	0.0007	0.0779	keejeybo	Transcript Identified by AceView	Coding
TC1000006798.hg.1	5.36	6.38	-2.03	0.0003	0.0585	CDC123	cell division cycle 123	Multiple_Complex
TC0700007796.hg.1	4.58	5.6	-2.04	0.0005	0.0679	SNORA22	Small nucleolar RNA SNORA22 [Source:RFAM;Acc:RF00414]	Small_RNA
TC0200010636.hg.1	4.85	5.88	-2.04	0.0005	0.0709	MAP2	microtubule associated protein 2	Multiple_Complex
TC1400009256.hg.1	6.75	7.78	-2.05	0.0007	0.0801	ATG14	autophagy related 14	Multiple_Complex
TC1900011985.hg.1	3.78	4.82	-2.05	2.05E-05	0.0163	DMWD	dystrophia myotonica, WD repeat containing	Multiple_Complex
TC1300008498.hg.1	3.86	4.9	-2.06	1.65E-05	0.0145			NonCoding
TSUnmapped00000211.hg.1	3.93	4.97	-2.06	0.0001	0.0418	SERTAD4	SERTA domain containing 4	NonCoding
TC1900008699.hg.1	3.63	4.68	-2.08	0.0007	0.0798	ZNF528	zinc finger protein 528	Multiple_Complex
TC2100006628.hg.1	3.78	4.84	-2.08	0.0011	0.0949	starfor	Transcript Identified by AceView	Coding
TC0200009037.hg.1	4.2	5.26	-2.08	0.0011	0.0935			NonCoding
TC1700009691.hg.1	2.85	3.91	-2.08	0.0006	0.0727	LOC100128288	uncharacterized LOC100128288	NonCoding
TC0100017844.hg.1	4.61	5.67	-2.08	7.44E-05	0.0307	NID1	nidogen 1	Multiple_Complex
TC0700008323.hg.1	5.46	6.52	-2.08	0.0011	0.0935	lokar	Transcript Identified by AceView	Unassigned
TC1800008165.hg.1	8.24	9.3	-2.09	0.0004	0.0631	AP001525.1		Precursor_microRNA
TC0800007193.hg.1	4.39	5.46	-2.09	0.0004	0.0616	CTD-2373N4.3	TEC	NonCoding
TC1100010207.hg.1	4.63	5.7	-2.1	0.0007	0.0789	SOX6; MIR6073	SRY box 6; microRNA 6073	Multiple_Complex
TC1700010980.hg.1	3.87	4.94	-2.1	0.0005	0.0717	RNU6-1201P	RNA, U6 small nuclear 1201, pseudogene [Source:HGNC Symbol;Acc:HGNC:48164]	Small_RNA
TC1300008104.hg.1	5.63	6.7	-2.11	0.0003	0.0561			NonCoding
TC1500006724.hg.1	4.31	5.39	-2.11	0.0004	0.0669	bludeeby	Transcript Identified by AceView, Entrez Gene ID(s) 100288516	Unassigned
TC1200010265.hg.1	4.98	6.06	-2.11	0.0003	0.0585	CAPRIN2	caprin family member 2	Multiple_Complex
TC0100009947.hg.1	3.3	4.38	-2.11	0.001	0.0917	nakoybu	Transcript Identified by AceView	Unassigned
TC1100010871.hg.1	3.83	4.91	-2.11	0.0003	0.0523	RP11-100N3.2		Pseudogene
TC0X00007543.hg.1	5.84	6.92	-2.12	6.30E-05	0.0283	zawbaw	Transcript Identified by AceView	Multiple_Complex

TC0700013298.hg.1	4.27	5.36	-2.12	0.0012	0.0964	muzeyby	Transcript Identified by AceView	Unassigned
TC0500011728.hg.1	4.2	5.29	-2.12	0.0013	0.1	roplaby	Transcript Identified by AceView	Unassigned
TC0X00010073.hg.1	6.33	7.41	-2.12	0.0008	0.0826	snady	Transcript Identified by AceView	Coding
TC1000010507.hg.1	8.73	9.82	-2.13	0.0004	0.0658	buskee; FAM25D; FAM25E; zawskaw	Transcript Identified by AceView; family with sequence similarity 25, member D [Source:HGNC Symbol;Acc:23588]; family with sequence similarity 25, member E [Source:HGNC Symbol;Acc:HGNC:23587]	Multiple_Complex
TC0500006923.hg.1	4.98	6.07	-2.13	0.0006	0.0769	klawstabu	Transcript Identified by AceView	Coding
TC0900007347.hg.1	5.44	6.54	-2.14	0.0006	0.0744	plervobu	Transcript Identified by AceView	Coding
TC0300006791.hg.1	5.94	7.04	-2.15	0.0001	0.0372	KAT2B	K(lysine) acetyltransferase 2B	Multiple_Complex
TC1500010305.hg.1	6.05	7.16	-2.15	0.0009	0.0877			NonCoding
TC1200006656.hg.1	4.08	5.18	-2.15	0.0007	0.0809	C12orf57; RNU7-1	chromosome 12 open reading frame 57; RNA, U7 small nuclear 1	Multiple_Complex
TC0700007968.hg.1	6.05	7.15	-2.15	0.0011	0.0935	GTF2IP1; GTF2IP4	general transcription factor IIi pseudogene 1; general transcription factor IIi, pseudogene 4	Multiple_Complex
TC0300012050.hg.1	6.55	7.66	-2.15	0.0001	0.0412	nekeme	Transcript Identified by AceView	Coding
TC1100006494.hg.1	4.34	5.46	-2.16	0.0008	0.0828	CD151	CD151 molecule (Raph blood group)	Multiple_Complex
TSUnmapped00000012.hg.1	5.23	6.34	-2.16	0.0005	0.0709	LRP6	LDL receptor related protein 6	Coding
TC0700011911.hg.1	6.87	7.98	-2.17	0.0002	0.0485	RPS3AP26	ribosomal protein S3a pseudogene 26	Pseudogene
TC1500007628.hg.1	4.5	5.62	-2.17	0.0011	0.0935			NonCoding
TC0X00010763.hg.1	5.59	6.71	-2.17	0.0004	0.0651	snudo	Transcript Identified by AceView	Coding
TC0100010438.hg.1	4.13	5.24	-2.17	0.0002	0.0428	RP11-506O24.2	novel transcript	NonCoding
TC1300008373.hg.1	4.6	5.72	-2.17	0.0002	0.0509			NonCoding
TC1600008462.hg.1	3.9	5.02	-2.17	0.001	0.0891			NonCoding
TC1100006731.hg.1	6.41	7.54	-2.18	6.85E-05	0.0296	ILK	integrin linked kinase	Multiple_Complex
TC2100006632.hg.1	3.43	4.56	-2.18	0.0003	0.0586	flarvee	Transcript Identified by AceView	Unassigned
TC0600008615.hg.1	5.73	6.86	-2.19	0.0005	0.0679			NonCoding
TC1200009930.hg.1	4.78	5.91	-2.2	9.12E-05	0.0338	TAS2R63P	taste receptor, type 2, member 63, pseudogene	Multiple_Complex
TC1700011578.hg.1	4.96	6.09	-2.2	0.0004	0.0625	ABCA6	ATP binding cassette subfamily A member 6	Multiple_Complex
TC0700010857.hg.1	5.5	6.64	-2.2	0.0002	0.0428	GLI3	GLI family zinc finger 3	Multiple_Complex
TC0300009703.hg.1	4.33	5.47	-2.21	0.0002	0.0436	SNORD66	small nucleolar RNA, C/D box 66	Small_RNA
TC0700008517.hg.1	4.91	6.05	-2.21	0.0009	0.0862	ZKSCAN1	zinc finger with KRAB and SCAN domains 1	Multiple_Complex
TC1800008116.hg.1	4.35	5.49	-2.21	0.0006	0.0734	AFG3L2	AFG3-like AAA ATPase 2	Multiple_Complex
TC0500008923.hg.1	6.26	7.4	-2.21	0.0002	0.047	verskoyby	Transcript Identified by AceView	Unassigned

TC0X00008009.hg.1	6.26	7.4	-2.21	0.0002	0.045	TCEAL4	transcription elongation factor A (SII)-like 4	Multiple_Complex
TC1000007379.hg.1	2.83	3.98	-2.21	1.78E-05	0.0154			NonCoding
TC2000008995.hg.1	3.78	4.92	-2.22	0.0002	0.0452	LINC00657	long intergenic non-protein coding RNA 657	NonCoding
TC0400012741.hg.1	6.58	7.73	-2.22	0.0006	0.0746			NonCoding
TC1100011971.hg.1	10.97	12.13	-2.22	0.0008	0.0832	AP004242.1		Precursor_microRNA
TC1800006590.hg.1	5.4	6.55	-2.23	0.0007	0.0801	weeso	Transcript Identified by AceView	Unassigned
TC0X00007396.hg.1	6.16	7.32	-2.23	0.0008	0.0817			NonCoding
TC0300011257.hg.1	6.17	7.33	-2.23	0.001	0.0891	fykobo	Transcript Identified by AceView	Unassigned
TC2100007987.hg.1	5.23	6.41	-2.26	0.0012	0.0988			NonCoding
TC0600011174.hg.1	5.86	7.03	-2.26	0.0005	0.0709	kluklybu	Transcript Identified by AceView	Coding
TC0200016253.hg.1	5.24	6.42	-2.26	0.0011	0.0945	MIR4440	microRNA 4440	Precursor_microRNA
TC1200012037.hg.1	5.87	7.05	-2.27	0.0003	0.0585			NonCoding
TC0X00010605.hg.1	5.27	6.46	-2.28	0.0008	0.0826			NonCoding
TC0100012143.hg.1	4.15	5.34	-2.28	0.0011	0.0945			NonCoding
TC1700012207.hg.1	4.94	6.13	-2.28	0.0008	0.0842	EPN2; EPN2-IT1	epsin 2; EPN2 intronic transcript 1	Multiple_Complex
TC1300008916.hg.1	6.75	7.93	-2.28	0.0011	0.0947	CAB39L	calcium binding protein 39-like	Multiple_Complex
TC0600012148.hg.1	5.03	6.23	-2.29	5.04E-05	0.0254	meyome	Transcript Identified by AceView	Coding
TC0300007930.hg.1	3.18	4.37	-2.29	1.14E-05	0.0119			NonCoding
TC0400010785.hg.1	4.6	5.8	-2.3	0.0003	0.055	IGFBP7	insulin like growth factor binding protein 7	Multiple_Complex
TC0300009356.hg.1	3.93	5.13	-2.3	0.0008	0.0826	jargeybu	Transcript Identified by AceView	Coding
TC0200016709.hg.1	4.46	5.67	-2.31	4.77E-05	0.0245	LINC00342	long intergenic non-protein coding RNA 342	NonCoding
TC0100009896.hg.1	3.35	4.56	-2.31	0.0004	0.0632	RNU2-17P	RNA, U2 small nuclear 17, pseudogene [Source:HGNC Symbol;Acc:HGNC:48510]	Small_RNA
TC0100006956.hg.1	3.94	5.15	-2.31	0.0001	0.0373	sneyleebo	Transcript Identified by AceView	Coding
TC1500008522.hg.1	7.09	8.3	-2.32	0.0006	0.0753	glyswar	Transcript Identified by AceView	Unassigned
TC2100006639.hg.1	6.38	7.59	-2.32	9.78E-05	0.035	marsu	Transcript Identified by AceView	Unassigned
TC0400008557.hg.1	5.76	6.97	-2.32	0.0005	0.0679	USP53	ubiquitin specific peptidase 53	Multiple_Complex
TC1600008505.hg.1	11.61	12.83	-2.33	0.0007	0.0779	WWOX	WW domain containing oxidoreductase	Multiple_Complex
TC1000006434.hg.1	5.55	6.77	-2.33	0.0006	0.0762	ZMYND11	zinc finger, MYND-type containing 11	Multiple_Complex
TC1000009737.hg.1	5.65	6.87	-2.33	0.0002	0.0499	ITIH5	inter-alpha-trypsin inhibitor heavy chain family, member 5	Multiple_Complex
TC0Y00006593.hg.1	6.06	7.29	-2.33	0.0001	0.038	PCMTD1P1	protein-L-isoaspartate (D-aspartate) O-methyltransferase domain containing 1 pseudogene 1 [Source:HGNC Symbol;Acc:HGNC:38804]	Multiple_Complex
TC2000008120.hg.1	3.84	5.06	-2.34	0.0009	0.0862			NonCoding

TC0100008067.hg.1	4.41	5.64	-2.34	0.0005	0.0712	marpla	Transcript Identified by AceView	Unassigned
TC0100008138.hg.1	4.01	5.24	-2.34	0.0008	0.0829	RP11-630I5.1		Pseudogene
TC0800008510.hg.1	4.75	5.98	-2.35	0.0008	0.0841	OXR1	oxidation resistance 1	Multiple_Complex
TC0300012895.hg.1	5.41	6.65	-2.35	0.001	0.092	RP11-305K5.1	novel transcript, antisense to KCNAB1	NonCoding
TC2100007073.hg.1	6.24	7.49	-2.36	0.0003	0.0604	AP001429.1	novel transcript, sense intronic to TTC3	NonCoding
TC0700008265.hg.1	5.17	6.41	-2.37	0.0005	0.0687	ADAM22	ADAM metallopeptidase domain 22	Multiple_Complex
TC0500009589.hg.1	4.73	5.98	-2.37	0.0008	0.0826			NonCoding
TC1500006790.hg.1	4.58	5.83	-2.38	0.0003	0.0585	sawroy	Transcript Identified by AceView	Coding
TC1100009741.hg.1	4.26	5.52	-2.38	0.0005	0.0716	lergler	Transcript Identified by AceView	Coding
TC0400010786.hg.1	5.78	7.04	-2.39	0.0004	0.0615	peegaw	Transcript Identified by AceView	Unassigned
TC1000011493.hg.1	4.83	6.09	-2.39	0.0006	0.0735	tutiro	Transcript Identified by AceView	Coding
TC1700007164.hg.1	5.19	6.45	-2.39	0.0003	0.0547	minema	Transcript Identified by AceView	Coding
TC0200016254.hg.1	6.26	7.52	-2.39	0.0009	0.0859	AC017028.11; AC017028.5		Precursor_microRNA
TC1000010292.hg.1	4.64	5.9	-2.4	0.0011	0.0945	PARD3	par-3 family cell polarity regulator	Multiple_Complex
TC0900006474.hg.1	5.59	6.85	-2.4	0.0004	0.0625			NonCoding
TC0100010735.hg.1	4.1	5.36	-2.4	0.0008	0.0849	RASAL2	RAS protein activator like 2	Multiple_Complex
TC2200007869.hg.1	5.47	6.73	-2.4	0.001	0.091			NonCoding
TC0100014127.hg.1	4.18	5.44	-2.4	0.0008	0.0834	SPATA6	spermatogenesis associated 6	Multiple_Complex
TC0500012291.hg.1	3.77	5.04	-2.41	8.04E-05	0.0318			NonCoding
TC1100006888.hg.1	4	5.28	-2.42	4.77E-05	0.0245	TEAD1	TEA domain family member 1 (SV40 transcriptional enhancer factor)	Multiple_Complex
TC1900008530.hg.1	4.22	5.5	-2.44	0.0004	0.0631	smeetee	Transcript Identified by AceView	Unassigned
TC0100012435.hg.1	4.08	5.36	-2.44	0.0012	0.0995	RP11-54O7.10		Multiple_Complex
TC1300007778.hg.1	4.79	6.08	-2.44	6.53E-05	0.0289	hemumi	Transcript Identified by AceView	Unassigned
TC0X00007906.hg.1	3.43	4.72	-2.44	0.0004	0.0643	EEF1A1P15	eukaryotic translation elongation factor 1 alpha 1 pseudogene 15 [Source:HGNC Symbol;Acc:HGNC:3198]	Multiple_Complex
TC1900010361.hg.1	5.75	7.04	-2.44	0.0004	0.0644	SNORA68	Small nucleolar RNA SNORA68 [Source:RFAM;Acc:RF00263]	Small_RNA
TC0600011735.hg.1	4.99	6.28	-2.45	0.0007	0.0785	blarvabo	Transcript Identified by AceView	Unassigned
TC1500008523.hg.1	4.12	5.42	-2.45	0.0005	0.0709	darzee	Transcript Identified by AceView	Unassigned
TC0800012435.hg.1	5.43	6.72	-2.46	0.0002	0.0444	ZFAND1	zinc finger, AN1-type domain 1	Multiple_Complex
TC0200012442.hg.1	8.34	9.63	-2.46	0.0004	0.0663	AC067957.1		Precursor_microRNA
TC2100006510.hg.1	3.82	5.12	-2.46	0.0003	0.0601			NonCoding

TC0100011226.hg.1	3.68	4.98	-2.46	0.0011	0.0942	nuplar	Transcript Identified by AceView	Unassigned
TC0200014719.hg.1	6.42	7.73	-2.47	0.0009	0.0881	BAZ2B	bromodomain adjacent to zinc finger domain 2B	Multiple_Complex
TC0100006568.hg.1	4.55	5.86	-2.47	0.0001	0.0419	tira	Transcript Identified by AceView	Unassigned
TC0300012600.hg.1	6.16	7.47	-2.48	3.99E-05	0.0222	RN7SKP124	RNA, 7SK small nuclear pseudogene 124 [Source:HGNC Symbol;Acc:HGNC:45848]	NonCoding
TC0900007113.hg.1	5.3	6.61	-2.49	0.0001	0.0402	RECK	reversion-inducing-cysteine-rich protein with kazal motifs	Multiple_Complex
TC0800011074.hg.1	4.75	6.07	-2.49	0.0005	0.0693	C8orf88	chromosome 8 open reading frame 88	Multiple_Complex
TC0500011067.hg.1	5.12	6.43	-2.49	0.0007	0.0801			NonCoding
TC0100013229.hg.1	3.69	5.01	-2.49	0.0008	0.0842	HSPG2	heparan sulfate proteoglycan 2	Multiple_Complex
TC0300006910.hg.1	6.75	8.07	-2.5	0.0005	0.0696	hikome	Transcript Identified by AceView	Coding
TC0400011218.hg.1	4.25	5.57	-2.5	0.0002	0.0435			NonCoding
TC0800010431.hg.1	5.66	6.98	-2.5	0.0005	0.0696	AC087348.1		Precursor_microRNA
TC0400008551.hg.1	4.15	5.47	-2.5	0.0012	0.0976	SYNPO2	synaptopodin 2	Multiple_Complex
TC0X00009356.hg.1	6.05	7.38	-2.51	0.0007	0.0791	TMEM47	transmembrane protein 47	Coding
TC0200008829.hg.1	6.28	7.61	-2.51	0.0005	0.0686	peeswarby	Transcript Identified by AceView	Coding
TC0200008839.hg.1	6.28	7.61	-2.51	0.0005	0.0686	syswarby	Transcript Identified by AceView	Coding
TC1100012568.hg.1	6.38	7.72	-2.52	0.0006	0.0769			NonCoding
TC1800007440.hg.1	6.1	7.43	-2.52	2.71E-05	0.0183	ZNF532	zinc finger protein 532	Multiple_Complex
TC0100009533.hg.1	6.2	7.53	-2.52	0.0004	0.0624			NonCoding
TC0300009545.hg.1	4.06	5.39	-2.52	2.68E-05	0.0183	gleeskeeby; mikayu	Transcript Identified by AceView	Multiple_Complex
TCUn_GL000220v100006432.hg.1	8.48	9.82	-2.54	2.69E-05	0.0183	RNA5-8S5	RNA, 5.8S ribosomal 5	Multiple_Complex
TC0400006579.hg.1	7.56	8.9	-2.54	0.001	0.092	ADD1	adducin 1 (alpha)	Multiple_Complex
TC1200007844.hg.1	5.21	6.56	-2.54	0.0002	0.0467	RBMS2	RNA binding motif, single stranded interacting protein 2	Multiple_Complex
TC1600007568.hg.1	6.53	7.89	-2.56	0.001	0.0891			NonCoding
TC1900006802.hg.1	4.25	5.6	-2.56	0.0002	0.0436	CTD-2396E7.11	novel transcript, antisense to DENND1C	NonCoding
TC0100017100.hg.1	4.14	5.49	-2.56	0.0002	0.045	EIF2D	eukaryotic translation initiation factor 2D	Multiple_Complex
TC1700009713.hg.1	5.59	6.95	-2.56	0.0008	0.0842	STX8	syntaxin 8	Multiple_Complex
TC0200006962.hg.1	7.72	9.09	-2.57	0.0002	0.0448	C2orf84	Transcript Identified by AceView, Entrez Gene ID(s) 653140; 375190	Unassigned
TC0700008130.hg.1	4.94	6.3	-2.57	0.0012	0.0964			NonCoding
TC0300013843.hg.1	3.75	5.11	-2.58	8.25E-05	0.0319	ADAMTS9-AS2	ADAMTS9 antisense RNA 2	NonCoding
TC0900009136.hg.1	4.77	6.15	-2.59	0.0012	0.0988	yutura	Transcript Identified by AceView	Unassigned
TC1500008140.hg.1	4.53	5.91	-2.59	6.20E-05	0.0282	UBE2Q2P8	ubiquitin conjugating enzyme E2Q family member 2 pseudogene 8 [Source:HGNC	Multiple_Complex

							Symbol;Acc:HGNC:49521]	
TC2000009073.hg.1	8.53	9.91	-2.6	0.001	0.0906	SNORA71D	small nucleolar RNA, H/ACA box 71D	Small_RNA
TC1300009511.hg.1	5.19	6.57	-2.61	0.0002	0.0436	swoynor	Transcript Identified by AceView	Unassigned
TC0200007261.hg.1	5.72	7.1	-2.61	0.0012	0.0963	QPCT	glutaminyl-peptide cyclotransferase	Multiple_Complex
TC0600009669.hg.1	5.39	6.78	-2.62	0.0007	0.0791	ADGRG6	adhesion G protein-coupled receptor G6	Multiple_Complex
TC1800007611.hg.1	4.68	6.07	-2.62	0.0002	0.0461	shawsor	Transcript Identified by AceView	Coding
TC0500006924.hg.1	6.44	7.83	-2.62	0.0005	0.0692	RP11-823P9.4	TEC	NonCoding
TC0100015568.hg.1	17.9	19.29	-2.62	1.80E-05	0.0154	U1	U1 spliceosomal RNA [Source:RFAM;Acc:RF00003]	Small_RNA
TC0900007436.hg.1	4.53	5.92	-2.63	0.0005	0.0697	mehumu	Transcript Identified by AceView	Unassigned
TC0800010163.hg.1	5.44	6.84	-2.64	0.0008	0.0845	FGFR1	fibroblast growth factor receptor 1	Multiple_Complex
TC1000007634.hg.1	3.5	4.9	-2.64	0.0011	0.0952			NonCoding
TC0900012173.hg.1	6.86	8.26	-2.64	0.0009	0.0851	GARNL3	GTPase activating Rap/RanGAP domain-like 3	Multiple_Complex
TC0200015034.hg.1	5.23	6.63	-2.65	0.0005	0.0697			NonCoding
TC0500010463.hg.1	6.68	8.08	-2.65	0.0013	0.1	glorplarby	Transcript Identified by AceView	Unassigned
TC1000008044.hg.1	9.8	11.21	-2.66	0.0002	0.0467	GLUD1P3	glutamate dehydrogenase 1 pseudogene 3	Multiple_Complex
TC0300009547.hg.1	6.1	7.5	-2.66	0.0006	0.0741	mykoybu	Transcript Identified by AceView	Unassigned
TC0600012215.hg.1	7.17	8.59	-2.66	0.0008	0.0838	AL590874.1		Precursor_microRNA
TC0500011705.hg.1	7.39	8.81	-2.67	0.0011	0.0942	NREP	neuronal regeneration related protein	Multiple_Complex
TC0400009147.hg.1	6.6	8.02	-2.69	0.0003	0.0529	jeespyby	Transcript Identified by AceView	Unassigned
TC0900007352.hg.1	4.08	5.51	-2.69	2.82E-05	0.0186	LOC103908605; GLIDR; RP11-211N8.2	Homo sapiens uncharacterized LOC103908605 (LOC103908605), long non-coding RNA.; glioblastoma down-regulated RNA [Source:EntrezGene;Acc:389741]; novel transcript	NonCoding
TC1700011320.hg.1	7.23	8.66	-2.69	0.0009	0.0874	nomor	Transcript Identified by AceView	Coding
TC1000010008.hg.1	6.13	7.56	-2.69	0.0002	0.0452	slorklabu	Transcript Identified by AceView	Coding
TC0100015629.hg.1	2.86	4.29	-2.69	0.0009	0.0871	FMO5	flavin containing monooxygenase 5	Multiple_Complex
TC1900010791.hg.1	5.74	7.17	-2.7	0.0003	0.0605			NonCoding
TC1800006676.hg.1	3.23	4.66	-2.71	9.21E-05	0.0339	peykloy	Transcript Identified by AceView	Coding
TC0400012644.hg.1	3.24	4.68	-2.71	0.0006	0.0751	SORBS2	sorbin and SH3 domain containing 2	Multiple_Complex
TC1700006820.hg.1	4.5	5.94	-2.72	0.0007	0.0787	RNU7-43P	RNA, U7 small nuclear 43 pseudogene [Source:HGNC Symbol;Acc:HGNC:34139]	Small_RNA
TC1600009732.hg.1	4.99	6.43	-2.72	0.0011	0.0935	shorzu	Transcript Identified by AceView	Unassigned

TC2000006755.hg.1	4.04	5.48	-2.72	0.0003	0.0528	NDUFAF5	NADH dehydrogenase (ubiquinone) complex I, assembly factor 5	Multiple_Complex
TC1100012318.hg.1	4.1	5.55	-2.72	0.0011	0.0935	IL18	interleukin 18	Multiple_Complex
TC0100015400.hg.1	8.57	10.01	-2.73	0.0002	0.0425	MIR548AC	microRNA 548ac	Precursor_microRNA
TC0100010434.hg.1	6.25	7.7	-2.73	0.0012	0.0964	kerbar	Transcript Identified by AceView	Coding
TC0600014374.hg.1	7.03	8.47	-2.73	0.0003	0.0555	PHF10	PHD finger protein 10	Multiple_Complex
TC0400008383.hg.1	5.04	6.49	-2.73	0.0011	0.0935			NonCoding
TC0600010545.hg.1	5.44	6.9	-2.74	5.48E-06	0.0081			NonCoding
TC2200006437.hg.1	8.01	9.46	-2.74	0.0005	0.0693			NonCoding
TC1000007356.hg.1	4.62	6.07	-2.74	0.0008	0.0828	shawsmu	Transcript Identified by AceView	Coding
TC1000006437.hg.1	5.29	6.75	-2.75	0.0009	0.0857	sneyskey	Transcript Identified by AceView	Coding
TC2200008745.hg.1	5.72	7.18	-2.76	0.0003	0.0609	SNORD83A	small nucleolar RNA, C/D box 83A	Small_RNA
TC0500010540.hg.1	5.13	6.59	-2.76	0.0007	0.0775	LIFR	leukemia inhibitory factor receptor alpha	Multiple_Complex
TC0100018059.hg.1	4.38	5.85	-2.76	0.0002	0.0467			NonCoding
TC0400011574.hg.1	7.67	9.14	-2.77	0.0003	0.0556			NonCoding
TC1500007087.hg.1	4.6	6.07	-2.77	0.0007	0.0779	snyzobu	Transcript Identified by AceView	Unassigned
TC1500006569.hg.1	3.55	5.03	-2.78	3.27E-05	0.0195	SNORD108; SNORD64; PWAR5	small nucleolar RNA, C/D box 108; small nucleolar RNA, C/D box 64; Prader Willi/Angelman region RNA 5	Multiple_Complex
TC0800009662.hg.1	4.97	6.45	-2.79	0.0004	0.0667	warslobu	Transcript Identified by AceView	Unassigned
TC1400008229.hg.1	5.84	7.33	-2.79	0.0006	0.0751	MEG3; MIR770	maternally expressed 3 (non-protein coding); microRNA 770	Multiple_Complex
TC0900010273.hg.1	4.56	6.04	-2.8	0.0003	0.0529	skorskubu	Transcript Identified by AceView	Unassigned
TC1900007954.hg.1	3.42	4.9	-2.8	0.001	0.0932	chysey	Transcript Identified by AceView	Coding
TC1700011321.hg.1	3.64	5.13	-2.8	0.0003	0.0604	pawmor	Transcript Identified by AceView	Coding
TC0700012838.hg.1	6.22	7.71	-2.81	0.001	0.0891	bloyshyby	Transcript Identified by AceView	Unassigned
TC1000011792.hg.1	5.76	7.26	-2.81	0.0002	0.0486	soremu	Transcript Identified by AceView	Unassigned
TC2200008167.hg.1	6.52	8.01	-2.81	0.0005	0.0717			NonCoding
TC1900011013.hg.1	3.92	5.41	-2.82	0.0004	0.0672	swywa	Transcript Identified by AceView	Multiple_Complex
TC0100013600.hg.1	4.93	6.42	-2.82	0.0012	0.0964			NonCoding
TC2000008473.hg.1	3.62	5.12	-2.84	0.0001	0.0419	KIF16B	kinesin family member 16B	Multiple_Complex
TC0500011648.hg.1	3.97	5.48	-2.84	0.0011	0.0949	EFNA5	ephrin-A5	Multiple_Complex
TC0400007574.hg.1	4.59	6.1	-2.85	0.0007	0.0774	tyboybo	Transcript Identified by AceView	Unassigned
TC0200010536.hg.1	4.58	6.09	-2.85	0.0001	0.0373	PARD3B	par-3 family cell polarity regulator beta	Multiple_Complex
TC0700007178.hg.1	10.35	11.87	-2.87	0.0006	0.0769	7-Sep	septin 7	Multiple_Complex
TC1800009305.hg.1	3.2	4.72	-2.88	0.0007	0.0785			NonCoding
TC0200014796.hg.1	3.84	5.37	-2.89	9.02E-06	0.0104	kynorbo	Transcript Identified by AceView	Coding

TC1000007862.hg.1	9.4	10.93	-2.89	0.0003	0.0585	COX20P1	COX20 cytochrome c oxidase assembly factor pseudogene 1	Multiple_Complex
TC1200007959.hg.1	6.37	7.91	-2.9	0.0001	0.0396	MON2	MON2 homolog, regulator of endosome-to-Golgi trafficking	Multiple_Complex
TC0200013047.hg.1	7.92	9.46	-2.91	0.0008	0.0849	verdawbu	Transcript Identified by AceView	Unassigned
TC0X00006540.hg.1	7.99	9.53	-2.91	0.0001	0.0359			NonCoding
TC1700008709.hg.1	5.21	6.75	-2.91	0.0012	0.0981	BPTF	bromodomain PHD finger transcription factor	Multiple_Complex
TC1100008788.hg.1	6.64	8.18	-2.91	0.001	0.0888	SCARNA9	small Cajal body-specific RNA 9	Multiple_Complex
TC1800007863.hg.1	5.15	6.69	-2.91	0.0008	0.0826	ENOSF1	enolase superfamily member 1	Multiple_Complex
TC1500008988.hg.1	4.24	5.79	-2.91	0.0002	0.0476	GOLGA8A; GOLGA8B	golgin A8 family, member A; golgin A8 family, member B	Multiple_Complex
TC1800007186.hg.1	5.02	6.56	-2.92	0.0011	0.0947	SETBP1	SET binding protein 1	Multiple_Complex
TC2100006636.hg.1	5.85	7.4	-2.92	0.0008	0.0829			NonCoding
TC0500010061.hg.1	5.62	7.17	-2.92	0.0005	0.0679	CTD-2201E9.3	TEC	Unassigned
TC0600012404.hg.1	4.47	6.02	-2.93	8.21E-05	0.0319	RP5-991C6.4; neyjoy	Transcript Identified by AceView; novel transcript	Multiple_Complex
TC0900007506.hg.1	4.05	5.6	-2.93	0.0005	0.0703	mertawby	Transcript Identified by AceView	Coding
TC0900012233.hg.1	5.03	6.58	-2.94	0.0012	0.0981	slotoyby	Transcript Identified by AceView	Multiple_Complex
TC1000011669.hg.1	3.96	5.52	-2.95	0.0002	0.0476	LDB1	LIM domain binding 1	Multiple_Complex
TC1400009389.hg.1	4.97	6.53	-2.95	0.0005	0.0696	bychorbu	Transcript Identified by AceView	Coding
TC0500007535.hg.1	4	5.56	-2.96	0.0006	0.073			NonCoding
TC0X00008435.hg.1	5.46	7.03	-2.98	0.0006	0.0741	KF459411.1		Precursor_microRNA
TC0500012639.hg.1	8.07	9.65	-2.98	1.18E-06	0.0037	rorflee	Transcript Identified by AceView	Unassigned
TC0100011729.hg.1	5.59	7.18	-3	0.0002	0.0458	RP11-504P24.4; RP11-504P24.6; TCONS_l2_00002322	Salzman2013 ANNOTATED, ncRNA, OVERLAPTX, OVEXON best transcript TCONS_l2_00002322	Multiple_Complex
TC0400010242.hg.1	5.01	6.6	-3	0.0002	0.0455	PPARGC1A	peroxisome proliferator-activated receptor gamma, coactivator 1 alpha	Multiple_Complex
TC0100009590.hg.1	8.21	9.8	-3.01	0.0006	0.0741	dawpoybu	Transcript Identified by AceView	Coding
TC0100010028.hg.1	3.35	4.94	-3.01	0.0011	0.0942	LOR	loricrin	Coding
TC0100017636.hg.1	7.35	8.94	-3.02	0.0011	0.0949	RNA5S11	RNA, 5S ribosomal 11	Ribosomal
TC0100017637.hg.1	7.35	8.94	-3.02	0.0011	0.0949	RNA5S12	RNA, 5S ribosomal 12	Ribosomal
TC0100017638.hg.1	7.35	8.94	-3.02	0.0011	0.0949	RNA5S13	RNA, 5S ribosomal 13	Ribosomal
TC0100017639.hg.1	7.35	8.94	-3.02	0.0011	0.0949	RNA5S14	RNA, 5S ribosomal 14	Ribosomal
TC0100017640.hg.1	7.35	8.94	-3.02	0.0011	0.0949	RNA5S15	RNA, 5S ribosomal 15	Ribosomal
TC0100017641.hg.1	7.35	8.94	-3.02	0.0011	0.0949	RNA5S16	RNA, 5S ribosomal 16	Ribosomal
TC1300006481.hg.1	7.74	9.33	-3.02	0.0003	0.0591	ZMYM2	zinc finger, MYM-type 2	Multiple_Complex
TC1700008594.hg.1	4.93	6.53	-3.03	1.29E-05	0.0126	SNORD104	small nucleolar RNA, C/D box 104	Small_RNA
TC0200007961.hg.1	5.91	7.51	-3.04	0.0003	0.0559			NonCoding

TCUn_GL000219v1 00006438.hg.1	4.83	6.43	-3.04	0.0002	0.0429	LOC283788 ; AL592183.1	FSHD region gene 1 pseudogene	Multiple_Complex
TC1500006574.hg.1	3.12	4.72	-3.05	0.0002	0.049	SNORD116 -2	small nucleolar RNA, C/D box 116-2	Multiple_Complex
TC1200008727.hg.1	5.68	7.29	-3.05	0.0006	0.0735	kawney	Transcript Identified by AceView	Unassigned
TC0700007137.hg.1	4.02	5.64	-3.06	0.0003	0.0551	BBS9	Bardet-Biedl syndrome 9	Multiple_Complex
TC1100007692.hg.1	9.16	10.77	-3.07	0.0003	0.0559	glyny	Transcript Identified by AceView	Unassigned
TC2200008734.hg.1	7.92	9.54	-3.07	4.06E-05	0.0224	CBX7	chromobox homolog 7	Multiple_Complex
TC0200010748.hg.1	3.89	5.51	-3.08	0.0005	0.0686	AC007563. 5; RP11-574O16.1	novel transcript, antisense to IGFBP5 and TNP1; novel transcript	NonCoding
TC1500010281.hg.1	4.78	6.41	-3.08	0.0003	0.0565	UBE2Q2P1 2	ubiquitin conjugating enzyme E2Q family member 2 pseudogene 12 [Source:HGNC Symbol;Acc:HGNC:49523]	Multiple_Complex
TC0100006886.hg.1	6.23	7.85	-3.09	0.0006	0.0727			NonCoding
TC1400008963.hg.1	4.94	6.56	-3.09	0.0002	0.0476	MBIP	MAP3K12 binding inhibitory protein 1	Multiple_Complex
TC1200006643.hg.1	8.78	10.41	-3.1	0.0002	0.0477	PTMS	parathymosin	Multiple_Complex
TC0700007601.hg.1	6.67	8.3	-3.1	0.0003	0.054	rehora	Transcript Identified by AceView	Unassigned
TC1900008435.hg.1	9.81	11.45	-3.12	0.0002	0.0429	SEPW1	selenoprotein W, 1	Multiple_Complex
TC0X00011321.hg.1	6.49	8.13	-3.12	0.0009	0.0867	RP11-111F16.2		Multiple_Complex
TC0600012086.hg.1	5.59	7.23	-3.12	3.14E-06	0.0059			NonCoding
TC1600010518.hg.1	6.62	8.27	-3.13	0.0003	0.0559	AC009081. 1		Precursor_microRNA
TC1200006604.hg.1	4.04	5.68	-3.13	0.0002	0.0511	CD9	CD9 molecule	Multiple_Complex
TC0300008040.hg.1	5.89	7.54	-3.13	0.0006	0.0727			NonCoding
TC0100011446.hg.1	7.29	8.94	-3.14	0.0008	0.0841	MIR205HG; MIR205	MIR205 host gene; microRNA 205	Multiple_Complex
TC1500009711.hg.1	3.47	5.12	-3.15	0.0012	0.0976	tikiru	Transcript Identified by AceView	Unassigned
TC1000010065.hg.1	6.38	8.04	-3.15	0.0007	0.0791	ARHGAP21	Rho GTPase activating protein 21	Multiple_Complex
TC1700010019.hg.1	3.54	5.19	-3.15	0.0006	0.0735	MFAP4	microfibrillar associated protein 4	Multiple_Complex
TC0500011033.hg.1	6.88	8.54	-3.16	0.0005	0.0719	norstawbu	Transcript Identified by AceView	Unassigned
TC1200009961.hg.1	2.98	4.64	-3.16	0.0008	0.0845			NonCoding
TC0600009457.hg.1	6.46	8.12	-3.16	0.0004	0.063	slotoybo	Transcript Identified by AceView	Unassigned
TC0300012965.hg.1	6.62	8.28	-3.16	0.0013	0.1	AC021654. 1		Precursor_microRNA
TC0400008450.hg.1	5.81	7.47	-3.16	0.0002	0.0455	ANK2	ankyrin 2, neuronal	Multiple_Complex
TC0100018239.hg.1	6.65	8.31	-3.16	2.73E-05	0.0183	GIPC2	GIPC PDZ domain containing family, member 2	Coding
TC1300008510.hg.1	4.92	6.58	-3.17	9.95E-05	0.0353	RP11-490N5.3	TEC	Unassigned
TC1200012295.hg.1	5.95	7.61	-3.17	0.0001	0.0369	RP11-214K3.22	novel transcript, sense intronic to CCDC92	NonCoding
TC0400012452.hg.1	5.45	7.11	-3.17	0.0006	0.0763	FBXO8	F-box protein 8	Coding
TC1600007893.hg.1	4.06	5.73	-3.17	3.70E-05	0.021	FTO	fat mass and obesity associated	Multiple_Complex

TC1500009838.hg.1	3.7	5.37	-3.17	0.0002	0.0481			NonCoding
TC0300011403.hg.1	4.49	6.16	-3.18	0.0001	0.0415	MAGI1	membrane associated guanylate kinase, WW and PDZ domain containing 1	Multiple_Complex
TC0400012648.hg.1	3.49	5.17	-3.2	0.0002	0.0506	Y_RNA	Y RNA [Source:RFAM;Acc:RF00019]	NonCoding
TC0200013871.hg.1	5.87	7.55	-3.21	0.001	0.0912	worswarby	Transcript Identified by AceView	Coding
TC2100006495.hg.1	6.57	8.26	-3.23	0.0005	0.0681	CR381670.1		Precursor_microRNA
TC2200006439.hg.1	6.57	8.26	-3.23	0.0005	0.0681	CU459202.1		Precursor_microRNA
TC0700007132.hg.1	4.49	6.19	-3.24	0.0003	0.0591	FKBP9	FK506 binding protein 9	Multiple_Complex
TC1100009808.hg.1	5.33	7.04	-3.26	5.83E-05	0.0277	KCNQ1OT1	KCNQ1 opposite strand/antisense transcript 1 (non-protein coding)	NonCoding
TC0300012002.hg.1	3.48	5.18	-3.27	0.0005	0.0714	zobubu	Transcript Identified by AceView	Unassigned
TC0Y00007277.hg.1	7.29	9	-3.27	0.001	0.091	REREP2Y	arginine-glutamic acid dipeptide (RE) repeats pseudogene 2, Y-linked [Source:HGNC Symbol;Acc:HGNC:38796]	Multiple_Complex
TC0700009295.hg.1	7.03	8.75	-3.28	0.0005	0.0717	AC009245.3		Pseudogene
TC1700012401.hg.1	12.45	14.17	-3.29	0.0004	0.0658	SMARCE1	SWI/SNF related, matrix associated, actin dependent regulator of chromatin, subfamily e, member 1	Multiple_Complex
TC0200016259.hg.1	5.15	6.87	-3.29	0.0002	0.0489	AC017028.8		Precursor_microRNA
TC1700012463.hg.1	6.42	8.15	-3.3	0.0001	0.0412	LOC102723505; LOC102723517; SOX9-AS1; AC005152.3; karwee	uncharacterized LOC102723505; uncharacterized LOC102723517; SOX9 antisense RNA 1 [Source:HGNC Symbol;Acc:HGNC:49321]; Transcript Identified by AceView; novel transcript; SOX9 antisense RNA 1	NonCoding
TC0400010243.hg.1	3.77	5.49	-3.31	0.0006	0.0727	jarsmerby	Transcript Identified by AceView	Coding
TC1600011354.hg.1	8.09	9.82	-3.31	0.0007	0.0791	NPIPA2	nuclear pore complex interacting protein family, member A2	Coding
TC0300011338.hg.1	4.65	6.38	-3.32	0.0001	0.0396	lerverbo	Transcript Identified by AceView	Coding
TC2100006706.hg.1	5.84	7.57	-3.32	0.0002	0.0487			NonCoding
TC0500007697.hg.1	5.78	7.51	-3.33	0.0004	0.0667	RP11-589F5.4		Multiple_Complex
TC1400009270.hg.1	7.11	8.85	-3.33	3.49E-05	0.0204			NonCoding
TC1000006617.hg.1	7.89	9.63	-3.34	0.0002	0.047	NET1	neuroepithelial cell transforming 1	Multiple_Complex
TC0200016769.hg.1	6.77	8.51	-3.34	0.0007	0.0775			NonCoding
TC0200010765.hg.1	7.33	9.07	-3.35	0.0002	0.0429	AC010887.1		Precursor_microRNA
TC0500011027.hg.1	8.01	9.76	-3.37	0.0012	0.0969	sweyzorby	Transcript Identified by AceView	Coding
TC1100010111.hg.1	3.51	5.27	-3.38	0.0002	0.0482	teygoby	Transcript Identified by AceView	Unassigned

TC0600014143.hg.1	8.2	9.96	-3.38	0.0001	0.0424	PHF3	PHD finger protein 3	Multiple_Complex
TC1000007552.hg.1	9.38	11.14	-3.38	6.92E-05	0.0298	TCONS_l2_00003977	Jeck2013 ANNOTATED, INTERNAL, ncRNA, OVEXON best transcript TCONS_l2_00003977	Multiple_Complex
TC0200014947.hg.1	4.35	6.12	-3.41	1.88E-05	0.0156			NonCoding
TC0X00008002.hg.1	6.51	8.29	-3.41	7.11E-05	0.0303	NGFRAP1	nerve growth factor receptor (TNFRSF16) associated protein 1	Multiple_Complex
TC0400008633.hg.1	6.18	7.96	-3.42	0.0002	0.0427			NonCoding
TCUn_KI270742v100006435.hg.1	5.44	7.22	-3.42	0.0009	0.0872			NonCoding
TC1000008904.hg.1	3.49	5.27	-3.43	0.0003	0.0585	PDCD4; MIR4680	programmed cell death 4 (neoplastic transformation inhibitor); microRNA 4680	Multiple_Complex
TC1400007328.hg.1	4.94	6.72	-3.44	0.0012	0.0962	DAAM1	dishevelled associated activator of morphogenesis 1	Multiple_Complex
TC0200009902.hg.1	5.01	6.79	-3.44	0.0006	0.0727	NOSTRIN	nitric oxide synthase trafficking	Multiple_Complex
TC0500011407.hg.1	5.67	7.46	-3.44	0.0002	0.051	plocheeby	Transcript Identified by AceView	Coding
TSUnmapped00000023.hg.1	4.71	6.51	-3.48	0.0007	0.0769	SNORD36A	small nucleolar RNA, C/D box 36A [Source:HGNC Symbol;Acc:HGNC:10163]	Small_RNA
TC0900010119.hg.1	8.38	10.18	-3.49	0.0004	0.0634	MIR1299	microRNA 1299	Precursor_microRNA
TC0400008452.hg.1	3.59	5.39	-3.49	0.0005	0.0679	sheysnawby	Transcript Identified by AceView	Unassigned
TC1900007115.hg.1	7.05	8.86	-3.49	0.0001	0.0415	NFIX	nuclear factor I/X (CCAAT-binding transcription factor)	Multiple_Complex
TC0100008608.hg.1	4.36	6.16	-3.5	0.0009	0.0871	swaplu	Transcript Identified by AceView	Unassigned
TC0500013184.hg.1	5.77	7.58	-3.5	0.0005	0.0692	SMA4	glucuronidase, beta pseudogene	Multiple_Complex
TC0400012899.hg.1	7.47	9.27	-3.5	0.0001	0.0412			NonCoding
TC2000007251.hg.1	7	8.81	-3.5	0.0009	0.0871	MYL9	myosin light chain 9	Multiple_Complex
TC0900008026.hg.1	4.21	6.03	-3.53	0.0006	0.073			NonCoding
TC0200009774.hg.1	5.76	7.58	-3.54	7.42E-06	0.0095	TANC1	tetratricopeptide repeat, ankyrin repeat and coiled-coil containing 1	Multiple_Complex
TC0900010395.hg.1	7.44	9.27	-3.55	0.0004	0.0667	HSPB1P1	heat shock protein family B (small) member 1 pseudogene 1 [Source:HGNC Symbol;Acc:HGNC:5251]	Multiple_Complex
TC0200007761.hg.1	5.27	7.1	-3.56	0.0011	0.0952	hihora	Transcript Identified by AceView	Unassigned
TC0400010167.hg.1	4.19	6.02	-3.56	0.0012	0.0964	DCAF16	DDB1 and CUL4 associated factor 16	Multiple_Complex
TC0100008525.hg.1	4.79	6.63	-3.57	2.10E-05	0.0164	snawperbu	Transcript Identified by AceView	Unassigned
TC0100009184.hg.1	4.76	6.59	-3.57	0.0006	0.0741	snarber	Transcript Identified by AceView	Coding
TC0200007613.hg.1	3.99	5.82	-3.57	6.30E-05	0.0283	smerkabu	Transcript Identified by AceView	Unassigned

TC1300009812.hg.1	5.72	7.56	-3.58	0.0002	0.0445	ANKRD10-IT1; RP11-365P13.4	ANKRD10 intronic transcript 1 (non-protein coding) [Source:HGNC Symbol;Acc:HGNC:39891]; novel transcript, sense intronic to ANKRD10; ANKRD10 intronic transcript 1 [Source:HGNC Symbol;Acc:HGNC:39891]	NonCoding
TC0200008535.hg.1	4.2	6.04	-3.58	0.0002	0.0488	smeekybo	Transcript Identified by AceView	Coding
TC0700010960.hg.1	6.55	8.4	-3.59	1.35E-05	0.0128	TCONS_l2_00026555	Memczak2013 ALT_ACCEPTOR, ALT_DONOR, INTERNAL, intronic, ncRNA best transcript TCONS_l2_00026555	NonCoding
TC0700007805.hg.1	5.86	7.71	-3.61	0.0003	0.0523	SNORA63	Small nucleolar RNA SNORA63 [Source:RFAM;Acc:RF00092]	Multiple_Complex
TC0100006578.hg.1	6.97	8.82	-3.61	0.0001	0.0396	surawbo	Transcript Identified by AceView	Coding
TC0500011004.hg.1	6.2	8.06	-3.62	0.0009	0.0882	GUSBP3	glucuronidase, beta pseudogene 3	Multiple_Complex
TC0500010847.hg.1	4.81	6.67	-3.63	8.92E-05	0.0333	nypluby	Transcript Identified by AceView	Unassigned
TC0500012636.hg.1	3.27	5.13	-3.64	0.0004	0.0641			NonCoding
TC1100008928.hg.1	5.03	6.9	-3.64	0.0009	0.0852	sawjuby	Transcript Identified by AceView	Coding
TC0200013531.hg.1	5.23	7.1	-3.65	6.14E-05	0.028	KANSL3	KAT8 regulatory NSL complex subunit 3	Multiple_Complex
TC1500010279.hg.1	4.25	6.12	-3.65	8.50E-07	0.0031	UBE2Q2P11	ubiquitin conjugating enzyme E2Q family member 2 pseudogene 11 [Source:HGNC Symbol;Acc:HGNC:49522]	Multiple_Complex
TC0600009056.hg.1	5.19	7.06	-3.65	0.0006	0.0759	wamame	Transcript Identified by AceView	Coding
TC0100010420.hg.1	7.35	9.22	-3.66	0.0006	0.0765	leygoybu	Transcript Identified by AceView	Unassigned
TC0400010521.hg.1	7.91	9.78	-3.66	0.0001	0.0377			NonCoding
TC0100011770.hg.1	5.03	6.9	-3.66	8.49E-05	0.0326	EPHX1	epoxide hydrolase 1, microsomal (xenobiotic)	Multiple_Complex
TC0300011815.hg.1	5.68	7.56	-3.67	0.0011	0.0935	DCBLD2	discoidin, CUB and LCCL domain containing 2	Multiple_Complex
TC0700008112.hg.1	3.87	5.75	-3.67	0.0002	0.0506			NonCoding
TC0700012266.hg.1	4.87	6.74	-3.67	0.0004	0.0628			NonCoding
TC1200010191.hg.1	3.74	5.62	-3.68	0.0004	0.0669	smarner	Transcript Identified by AceView	Unassigned
TC1000007045.hg.1	4.09	5.98	-3.69	0.0004	0.063	glarmeyby	Transcript Identified by AceView	Unassigned
TC0400011535.hg.1	4.58	6.47	-3.69	0.0007	0.0787	DKK2	dickkopf WNT signaling pathway inhibitor 2	Multiple_Complex
TC0300012258.hg.1	4.38	6.26	-3.7	0.0006	0.0757	MIR548I1	microRNA 548i-1	Precursor_microRNA
TC0200008803.hg.1	5.21	7.09	-3.7	0.0009	0.0851	GCC2	GRIP and coiled-coil domain containing 2	Multiple_Complex
TC1200007425.hg.1	4.73	6.62	-3.71	0.0006	0.0769	ARID2	AT rich interactive domain 2 (ARID, RFX-like)	Multiple_Complex

TC0100013193.hg.1	7.52	9.41	-3.72	0.0009	0.0875	HP1BP3	heterochromatin protein 1, binding protein 3	Multiple_Complex
TC0900010197.hg.1	4.84	6.74	-3.72	0.0007	0.0787	RP11-111F5.3	putative novel transcript	NonCoding
TC1100011282.hg.1	3.91	5.81	-3.73	1.38E-05	0.0128	CD248	CD248 molecule, endosialin	Coding
TC0500011013.hg.1	8.53	10.43	-3.73	0.0007	0.0773	vostarby	Transcript Identified by AceView	Coding
TC2100006534.hg.1	3.14	5.05	-3.76	0.0008	0.0829			NonCoding
TC1000012552.hg.1	7.58	9.49	-3.76	4.09E-05	0.0225	FAM25G; FAM25C; FAM25BP	family with sequence similarity 25, member G; family with sequence similarity 25, member C; protein FAM25	Multiple_Complex
TC1900006524.hg.1	7.17	9.08	-3.77	0.0007	0.0811	CIRBP	cold inducible RNA binding protein	Multiple_Complex
TC0500013324.hg.1	6.75	8.67	-3.79	0.0004	0.0667	GUSBP9; TCONS_l2_00023779	Homo sapiens glucuronidase, beta pseudogene 9 (GUSBP9), non-coding RNA.; Salzman2013 ANNOTATED, INTERNAL, ncRNA, OVEXON best transcript TCONS_l2_00023779	Multiple_Complex
TC0100013271.hg.1	5.39	7.31	-3.8	0.0004	0.0637	rawabo	Transcript Identified by AceView	Coding
TC2100008543.hg.1	4.15	6.07	-3.8	0.0004	0.0631	ANKRD20A11P	ankyrin repeat domain 20 family, member A11, pseudogene	Multiple_Complex
TC0200016703.hg.1	8.61	10.54	-3.81	2.02E-05	0.0161	CHMP3	charged multivesicular body protein 3	Multiple_Complex
TC1200010372.hg.1	6.48	8.4	-3.81	0.001	0.0917			NonCoding
TC0100006939.hg.1	6.98	8.91	-3.82	0.0003	0.0598	blarawbo	Transcript Identified by AceView	Unassigned
TC1000012522.hg.1	5.6	7.54	-3.83	0.0008	0.0831	BEND7	BEN domain containing 7	Multiple_Complex
TC1000012050.hg.1	5.7	7.65	-3.85	3.44E-06	0.006	FGFR2	fibroblast growth factor receptor 2	Multiple_Complex
TC0400008642.hg.1	6.98	8.92	-3.85	0.0005	0.0681	Y_RNA	Y RNA [Source:RFAM;Acc:RF00019]	NonCoding
TC1200007397.hg.1	3.76	5.72	-3.87	0.0004	0.0658			NonCoding
TC1900009716.hg.1	10.72	12.67	-3.87	0.0001	0.0356			NonCoding
TC1600006673.hg.1	9.84	11.79	-3.87	0.0006	0.0769			NonCoding
TC0100011064.hg.1	4.47	6.42	-3.89	0.0007	0.0778	CFH	complement factor H	Multiple_Complex
TC0300009666.hg.1	5.05	7.01	-3.9	0.0012	0.0972	SNORA63	Small nucleolar RNA SNORA63 [Source:RFAM;Acc:RF00092]	Small_RNA
TC0300010394.hg.1	4.22	6.18	-3.9	0.0005	0.0679	AC090954.1		Precursor_microRNA
TC0100008262.hg.1	6.73	8.7	-3.91	0.0001	0.0359	rarmeebu	Transcript Identified by AceView	Coding
TC0500010941.hg.1	11.89	13.86	-3.91	0.0011	0.0949	flostarbu	Transcript Identified by AceView	Coding
TC1300008793.hg.1	7.11	9.08	-3.91	0.0008	0.0825	TSC22D1	TSC22 domain family, member 1	Multiple_Complex
TC1000012453.hg.1	7.54	9.51	-3.91	0.0001	0.0388	FAM25BP	protein FAM25	Multiple_Complex
TC0600008539.hg.1	4.93	6.91	-3.93	2.39E-05	0.0171	CD109	CD109 molecule	Multiple_Complex

TC1_KI270708v1_random00006437.hg.1	5.76	7.74	-3.95	6.95E-06	0.0092	AL137861.6		Precursor_microRNA
TC0800009508.hg.1	5.6	7.58	-3.96	0.0002	0.0485	cheegorby	Transcript Identified by AceView	Coding
TC1600009516.hg.1	7.39	9.38	-3.96	0.0001	0.0383	PKD1P6; NPIPP1	polycystic kidney disease 1 (autosomal dominant) pseudogene 6; nuclear pore complex interacting protein pseudogene 1	Multiple_Complex
TC0100011224.hg.1	4.51	6.5	-3.97	0.0002	0.0461			NonCoding
TC1300008046.hg.1	7.4	9.39	-3.98	0.0011	0.0935			NonCoding
TC0300012048.hg.1	6.88	8.87	-3.98	0.0005	0.0712	ZBTB20; MIR568	zinc finger and BTB domain containing 20; microRNA 568	Multiple_Complex
TC0500007641.hg.1	5.4	7.4	-4	0.0009	0.0867	PIK3R1	phosphoinositide-3-kinase, regulatory subunit 1 (alpha)	Multiple_Complex
TC1200012638.hg.1	4.29	6.29	-4	0.0002	0.0487	HOXC9	homeobox C9	Multiple_Complex
TC0200014718.hg.1	5.48	7.48	-4	0.0009	0.0872	meydo	Transcript Identified by AceView	Unassigned
TC0200015350.hg.1	4.56	6.56	-4.01	2.32E-05	0.0171	RFTN2	raftlin family member 2	Multiple_Complex
TC0700008748.hg.1	3.98	5.99	-4.02	2.38E-05	0.0171	tawkaw	Transcript Identified by AceView	Unassigned
TC0100006466.hg.1	7.05	9.06	-4.03	8.82E-05	0.0333	RP11-206L10.9; tisamo	novel transcript; Transcript Identified by AceView	Multiple_Complex
TC1100012508.hg.1	11.05	13.06	-4.04	0.0009	0.0851	RPS25	ribosomal protein S25	Multiple_Complex
TC0200013567.hg.1	6.74	8.76	-4.05	0.0008	0.0843	ANKRD36B	ankyrin repeat domain 36B	Multiple_Complex
TC0700012754.hg.1	5.56	7.58	-4.06	0.0011	0.0935	ATP6V0A4	ATPase, H+ transporting, lysosomal V0 subunit a4	Multiple_Complex
TC2200006446.hg.1	6.69	8.72	-4.06	4.31E-05	0.023			NonCoding
TC2200006447.hg.1	6.69	8.72	-4.06	4.31E-05	0.023			NonCoding
TC1200007741.hg.1	4.56	6.58	-4.06	0.0009	0.0857	AC012531.25	novel transcript	NonCoding
TC0700008351.hg.1	4.73	6.75	-4.07	8.90E-05	0.0333	GNG11	guanine nucleotide binding protein (G protein), gamma 11	Multiple_Complex
TC1100009287.hg.1	6.68	8.7	-4.07	0.0006	0.0736	ARHGEF12	Rho guanine nucleotide exchange factor (GEF) 12	Multiple_Complex
TC1200007687.hg.1	6.97	9	-4.07	0.0006	0.0745	EIF4B	eukaryotic translation initiation factor 4B	Multiple_Complex
TC2000007062.hg.1	8.37	10.4	-4.09	0.0006	0.0746	AC104301.1		Precursor_microRNA
TC0100017634.hg.1	8.04	10.08	-4.1	5.89E-05	0.0278	RNA5S9	RNA, 5S ribosomal 9	Multiple_Complex
TC1800007010.hg.1	4.82	6.86	-4.1	0.0008	0.0828	DSG1	desmoglein 1	Coding
TC1400008705.hg.1	5.36	7.4	-4.12	0.0011	0.0932	SLC7A8	solute carrier family 7 (amino acid transporter light chain, L system), member 8	Multiple_Complex
TC0900010118.hg.1	10.21	12.26	-4.13	5.32E-05	0.0261	AL353763.1		Precursor_microRNA
TC0100009999.hg.1	2.92	4.97	-4.13	0.0007	0.0798	LCE2C	late cornified envelope 2C	Coding
TC1700010723.hg.1	3.48	5.54	-4.15	5.49E-07	0.0023	PTRF	polymerase I and transcript release factor	Multiple_Complex
TC1200009834.hg.1	4.04	6.1	-4.16	0.0003	0.0564	MFAP5	microfibrillar associated protein 5	Multiple_Complex
TC0800010234.hg.1	4.4	6.46	-4.16	0.0005	0.0679	SFRP1	secreted frizzled-related protein 1	Multiple_Complex

TC0400008180.hg.1	6.39	8.45	-4.17	0.0005	0.0718	snawfloy	Transcript Identified by AceView	Unassigned
TC0900007768.hg.1	5.31	7.37	-4.18	3.32E-06	0.0059	LOC389765	kinesin family member 27 pseudogene	Multiple_Complex
TC1900010556.hg.1	6.62	8.69	-4.2	0.0006	0.0727	rawsarbo	Transcript Identified by AceView	Coding
TC1900010560.hg.1	6.62	8.69	-4.2	0.0006	0.0727	geyjer	Transcript Identified by AceView	Coding
TC1900010562.hg.1	6.62	8.69	-4.2	0.0006	0.0727	kajer	Transcript Identified by AceView	Coding
TC1900010563.hg.1	6.62	8.69	-4.2	0.0006	0.0727	kawjer	Transcript Identified by AceView	Coding
TC0100006725.hg.1	4.37	6.44	-4.2	1.94E-05	0.0156	PER3	period circadian clock 3	Multiple_Complex
TC0400008148.hg.1	6.19	8.26	-4.22	6.40E-05	0.0286	spoyblorby	Transcript Identified by AceView	Coding
TC0700008077.hg.1	8.52	10.6	-4.23	0.0004	0.0615	SNORA14A	small nucleolar RNA, H/ACA box 14A	Small_RNA
TC2000007435.hg.1	7.39	9.48	-4.24	0.0012	0.0964	IFT52	intraflagellar transport 52	Multiple_Complex
TC0500008712.hg.1	6.97	9.06	-4.25	0.0003	0.0581	MIR4461	microRNA 4461	Precursor_microRNA
TC0200015678.hg.1	3.47	5.56	-4.25	0.0002	0.0501	IGFBP5	insulin like growth factor binding protein 5	Multiple_Complex
TC0700011151.hg.1	7.97	10.06	-4.25	2.13E-05	0.0164	FKBP9P1	FK506 binding protein 9 pseudogene 1	Multiple_Complex
TC0500007285.hg.1	5.61	7.7	-4.26	4.39E-07	0.002			NonCoding
TC0X00006848.hg.1	5.71	7.81	-4.26	5.52E-05	0.0268	RPL7P58	ribosomal protein L7 pseudogene 58 [Source:HGNC Symbol;Acc:HGNC:49214]	Multiple_Complex
TC0200016019.hg.1	3.76	5.85	-4.26	0.0009	0.0861	SNORA75	small nucleolar RNA, H/ACA box 75	Small_RNA
TC0200008536.hg.1	9.23	11.33	-4.27	0.0005	0.0706	ANKRD36	Transcript Identified by AceView, Entrez Gene ID(s) 375248	Unassigned
TC1100012230.hg.1	4.6	6.7	-4.29	4.90E-05	0.025	EXPH5	exophilin 5	Multiple_Complex
TC0800011251.hg.1	7.58	9.68	-4.29	0.0004	0.0636	savawby	Transcript Identified by AceView	Unassigned
TC1500009662.hg.1	6.48	8.58	-4.29	9.03E-06	0.0104	woyzo	Transcript Identified by AceView	Unassigned
TC1200010590.hg.1	3.88	5.99	-4.31	1.87E-05	0.0156	SNORA2A	small nucleolar RNA, H/ACA box 2A	Small_RNA
TC0800008265.hg.1	4.49	6.61	-4.33	0.0007	0.0791	RP11-22C11.1		Multiple_Complex
TC14_GL000194v1_random00006434.hg.1	11.92	14.05	-4.37	0.0009	0.0882	AC145212.1		Precursor_microRNA
TC1300007491.hg.1	4.7	6.83	-4.38	2.33E-05	0.0171	KLF5	Kruppel-like factor 5 (intestinal)	Multiple_Complex
TC1700012284.hg.1	6.52	8.66	-4.41	0.0006	0.0738			NonCoding
TC0600009466.hg.1	10.59	12.74	-4.42	0.0004	0.0667			NonCoding
TC1600011489.hg.1	7.24	9.38	-4.43	0.0006	0.0741	NPIPA5	nuclear pore complex interacting protein family, member A5	Multiple_Complex
TC0300009541.hg.1	4.06	6.21	-4.43	0.0003	0.0525	waromo	Transcript Identified by AceView	Coding
TC1000008905.hg.1	4.69	6.84	-4.44	0.0009	0.0873	spypawby	Transcript Identified by AceView	Unassigned
TC0600013434.hg.1	3.83	5.99	-4.45	0.0001	0.0412			NonCoding
TC2200008604.hg.1	4.72	6.88	-4.47	4.43E-05	0.0234	RPS15AP38	ribosomal protein S15a pseudogene 38 [Source:HGNC Symbol;Acc:HGNC:36523]	Pseudogene

TC1200008466.hg.1	6.66	8.82	-4.47	8.24E-05	0.0319	NUDT4	nudix hydrolase 4	Multiple_Complex
TC1500010549.hg.1	5.28	7.44	-4.47	8.46E-05	0.0326			NonCoding
TC0500013335.hg.1	8.51	10.68	-4.48	2.92E-06	0.0057			NonCoding
TC0200007609.hg.1	6.82	8.99	-4.49	0.0002	0.0447	SPTBN1	spectrin, beta, non-erythrocytic 1	Multiple_Complex
TC0900008052.hg.1	5.07	7.24	-4.5	0.0007	0.0774	slawtoby	Transcript Identified by AceView	Unassigned
TCUn_GL000224v100006432.hg.1	8.82	11	-4.53	0.0004	0.0649	AL591856.5		Precursor_microRNA
TC0100014466.hg.1	5.38	7.57	-4.56	8.26E-07	0.0031			NonCoding
TC2200006440.hg.1	10.32	12.51	-4.57	0.0005	0.0724			NonCoding
TC0100013278.hg.1	5.53	7.72	-4.57	0.0002	0.0428	TCEA3	transcription elongation factor A (SII), 3	Multiple_Complex
TC0500011034.hg.1	4.55	6.75	-4.62	0.0013	0.1	cheeju	Transcript Identified by AceView	Unassigned
TC1500007403.hg.1	8.62	10.82	-4.62	8.14E-06	0.01			NonCoding
TC0400009258.hg.1	6.25	8.46	-4.62	0.0007	0.0801	PALLD	palladin, cytoskeletal associated protein	Multiple_Complex
TC0800009738.hg.1	4.28	6.49	-4.63	0.0006	0.0735	MIR548V	microRNA 548v	Precursor_microRNA
TC0600009353.hg.1	3.91	6.13	-4.66	0.0009	0.0875	TPD52L1	tumor protein D52-like 1	Multiple_Complex
TC0500013318.hg.1	7.48	9.7	-4.67	0.0008	0.0838	GUSBP3; SMA4	Homo sapiens glucuronidase, beta pseudogene 3 (GUSBP3), non-coding RNA.; Salzman2013 ANNOTATED, INTERNAL, ncRNA, OVEXON best transcript NR_029426; Salzman2013 ANNOTATED, ncRNA, OVEXON best transcript NR_029426	Multiple_Complex
TC1000008942.hg.1	4.57	6.79	-4.68	9.18E-06	0.0105	TCF7L2	transcription factor 7-like 2 (T-cell specific, HMG-box)	Multiple_Complex
TC0100010423.hg.1	7.1	9.33	-4.71	8.14E-06	0.01	sworloybu	Transcript Identified by AceView	Unassigned
TC14_GL000009v2_random00006452.hg.1	3.94	6.19	-4.74	1.32E-05	0.0127	RP11-435B5.3		NonCoding
TC0500013319.hg.1	6.32	8.57	-4.75	0.0007	0.0787	GUSBP9	glucuronidase, beta pseudogene 9	Multiple_Complex
TC1900010561.hg.1	4.64	6.89	-4.78	7.70E-05	0.0311	machee	Transcript Identified by AceView	Coding
TC0400008873.hg.1	7.24	9.51	-4.82	0.0003	0.0564	skaplyby	Transcript Identified by AceView	Coding
TC1200010028.hg.1	3.86	6.13	-4.82	0.0002	0.0453			NonCoding
TC0X00010512.hg.1	5.46	7.73	-4.83	0.0012	0.0996	CHRDL1	chordin-like 1	Coding
TC1600009524.hg.1	9.64	11.92	-4.87	0.0006	0.0751	NPIPA5	nuclear pore complex interacting protein family, member A5	Coding
TC0100014910.hg.1	5.13	7.42	-4.88	1.89E-06	0.0047	TGFBR3	transforming growth factor beta receptor III	Multiple_Complex
TC0M00006444.hg.1	10.84	13.13	-4.91	0.0003	0.0527			NonCoding
TC1100008624.hg.1	6.65	8.95	-4.93	0.0002	0.0491	RAB30-AS1	RAB30 antisense RNA 1 (head to head)	NonCoding
TC0X00010756.hg.1	6.9	9.2	-4.93	0.0007	0.0805	AL359973.1		Precursor_microRNA
TC1100010180.hg.1	4.01	6.32	-4.97	0.0005	0.0696	watomi	Transcript Identified by AceView	Coding

TC1400008717.hg.1	2.79	5.12	-5.02	0.0008	0.0831	MIR208A	microRNA 208a	Multiple_Complex
TC2200007147.hg.1	5.79	8.12	-5.03	0.0002	0.051			NonCoding
TC2100006491.hg.1	13.91	16.24	-5.05	0.0009	0.0861			NonCoding
TC0300012720.hg.1	4.12	6.46	-5.05	7.29E-08	0.0006	PLSCR4	phospholipid scramblase 4	Multiple_Complex
TC2200007842.hg.1	11.45	13.8	-5.07	0.0012	0.0988	CU463998.2		Precursor_microRNA
TC0100011341.hg.1	4.45	6.8	-5.07	0.0004	0.0624	RNU6-418P	RNA, U6 small nuclear 418, pseudogene [Source:HGNC Symbol;Acc:HGNC:47381]	Small_RNA
TC1900010860.hg.1	6.16	8.5	-5.07	2.10E-05	0.0164			NonCoding
TC1500008867.hg.1	6.88	9.23	-5.08	0.0001	0.0377	plergler	Transcript Identified by AceView	Unassigned
TC1500009037.hg.1	5.99	8.33	-5.09	0.0003	0.0557	MIR8063	microRNA 8063	Precursor_microRNA
TC1200012296.hg.1	4.69	7.04	-5.1	2.85E-05	0.0186	RP11-214K3.21	novel transcript, sense intronic to CCDC92	NonCoding
TC0800012451.hg.1	4.95	7.3	-5.12	0.0012	0.0993	EIF3E	eukaryotic translation initiation factor 3, subunit E	Multiple_Complex
TC0600009426.hg.1	4.42	6.78	-5.13	3.08E-05	0.0187	LAMA2	laminin, alpha 2	Multiple_Complex
TC0500011031.hg.1	5.22	7.59	-5.17	0.0003	0.0585			NonCoding
TC1500007321.hg.1	3.62	6	-5.2	0.0012	0.0981	skeyklo	Transcript Identified by AceView	Coding
TC0X00008198.hg.1	7.65	10.04	-5.22	3.19E-05	0.0192	PLS3	plastin 3	Multiple_Complex
TC0200006730.hg.1	5.86	8.26	-5.28	1.16E-06	0.0037	RN7SL674P	RNA, 7SL, cytoplasmic 674, pseudogene [Source:HGNC Symbol;Acc:HGNC:46690]	NonCoding
TC1900007819.hg.1	4.58	6.99	-5.3	0.0006	0.0735	ZNF302	zinc finger protein 302	Multiple_Complex
TC0600006762.hg.1	8.2	10.61	-5.31	1.37E-05	0.0128	MIR7641-2; RNA5SP202	Homo sapiens microRNA 7641-2 (MIR7641-2), microRNA.; 5S ribosomal pseudogene 202 [Source:HGNC Symbol;Acc:HGNC:43102]	Multiple_Complex
TC1700012283.hg.1	9.22	11.63	-5.31	7.33E-05	0.0307	LUC7L3	LUC7-like 3 pre-mRNA splicing factor	Multiple_Complex
TC1000010844.hg.1	9.13	11.55	-5.34	0.0011	0.0935	RUFY2	RUN and FYVE domain containing 2	Multiple_Complex
TC0200012282.hg.1	7.45	9.87	-5.35	0.0009	0.0877	ATL2	atlastin GTPase 2	Multiple_Complex
TC4_GL000008v2_random00006436.hg.1	6.75	9.18	-5.39	0.0009	0.0867	AL583842.2		Precursor_microRNA
TC1700010232.hg.1	7.99	10.44	-5.45	5.98E-06	0.0085	slarsmy	Transcript Identified by AceView	Coding
TC0400010704.hg.1	4.45	6.9	-5.46	0.0001	0.0359	LNX1	ligand of numb-protein X 1, E3 ubiquitin protein ligase	Multiple_Complex
TC1400010390.hg.1	5.68	8.13	-5.46	5.32E-07	0.0023	AHNAK2	AHNAK nucleoprotein 2	Multiple_Complex
TC1700010680.hg.1	3.01	5.46	-5.46	0.0002	0.0452	KRT14	keratin 14, type I	Multiple_Complex
TC1400010680.hg.1	5.91	8.37	-5.52	0.0007	0.0792	SNORD114-21	small nucleolar RNA, C/D box 114-21	Multiple_Complex
TC0400009475.hg.1	13.83	16.3	-5.53	7.23E-05	0.0306			NonCoding

TC0X00010703.hg.1	4.76	7.23	-5.53	3.32E-05	0.0197	rodo	Transcript Identified by AceView	Coding
TC1700006556.hg.1	5.18	7.66	-5.56	9.37E-05	0.0343	PAFAH1B1	platelet-activating factor acetylhydrolase 1b, regulatory subunit 1 (45kDa)	Multiple_Complex
TC1200010182.hg.1	6.47	8.96	-5.6	0.0006	0.0736	BHLHE41	basic helix-loop-helix family, member e41	Multiple_Complex
TC0800006780.hg.1	5.2	7.69	-5.61	0.0002	0.0451	plawgoyby	Transcript Identified by AceView	Coding
TC1700007777.hg.1	6.13	8.62	-5.64	5.09E-06	0.0079	THRA	thyroid hormone receptor, alpha	Multiple_Complex
TC0100014531.hg.1	5.56	8.05	-5.65	1.64E-05	0.0145	WLS	wntless Wnt ligand secretion mediator	Multiple_Complex
TC0100017626.hg.1	8.19	10.7	-5.67	3.02E-05	0.0186	RNA5S2; RNA5S1	RNA, 5S ribosomal 2; RNA, 5S ribosomal 1	Multiple_Complex
TC0100017627.hg.1	8.19	10.7	-5.67	3.02E-05	0.0186	RNA5S3; RNA5S2	RNA, 5S ribosomal 3; RNA, 5S ribosomal 2	Multiple_Complex
TC0100017628.hg.1	8.19	10.7	-5.67	3.02E-05	0.0186	RNA5S4; RNA5S3	RNA, 5S ribosomal 4; RNA, 5S ribosomal 3	Multiple_Complex
TC0100017629.hg.1	8.19	10.7	-5.67	3.02E-05	0.0186	RNA5S1; RNA5S5; RNA5S4	RNA, 5S ribosomal 1; RNA, 5S ribosomal 5; RNA, 5S ribosomal 4	Multiple_Complex
TC0100017630.hg.1	8.19	10.7	-5.67	3.02E-05	0.0186	RNA5S6; RNA5S7; RNA5S8; RNA5S5	RNA, 5S ribosomal 6; RNA, 5S ribosomal 7; RNA, 5S ribosomal 8; RNA, 5S ribosomal 5	Multiple_Complex
TC0100017631.hg.1	8.19	10.7	-5.67	3.02E-05	0.0186	RNA5S11; RNA5S12; RNA5S13; RNA5S14; RNA5S15; RNA5S16; RNA5S17; RNA5S6	RNA, 5S ribosomal 11; RNA, 5S ribosomal 12; RNA, 5S ribosomal 13; RNA, 5S ribosomal 14; RNA, 5S ribosomal 15; RNA, 5S ribosomal 16; RNA, 5S ribosomal 17; RNA, 5S ribosomal 6	Multiple_Complex
TC0100017632.hg.1	8.19	10.7	-5.67	3.02E-05	0.0186	RNA5S7	RNA, 5S ribosomal 7	Multiple_Complex
TC0100017633.hg.1	8.19	10.7	-5.67	3.02E-05	0.0186	RNA5S10; RNA5S8	RNA, 5S ribosomal 10; RNA, 5S ribosomal 8	Multiple_Complex
TC0100017635.hg.1	8.19	10.7	-5.67	3.02E-05	0.0186	RNA5S10	RNA, 5S ribosomal 10	Multiple_Complex
TC0100017642.hg.1	8.19	10.7	-5.67	3.02E-05	0.0186	RNA5S17	RNA, 5S ribosomal 17	Multiple_Complex
TC1100011972.hg.1	5.27	7.77	-5.68	2.11E-05	0.0164	flatubo	Transcript Identified by AceView	Coding
TC1000009522.hg.1	5.21	7.71	-5.69	0.001	0.0926			NonCoding
TC0800011220.hg.1	5.63	8.15	-5.74	5.96E-05	0.0279	skodaby	Transcript Identified by AceView	Coding
TC0800011171.hg.1	6.43	8.96	-5.76	0.0002	0.0436	UQCRB	ubiquinol-cytochrome c reductase binding protein	Multiple_Complex
TC0100018454.hg.1	5.2	7.73	-5.77	1.13E-06	0.0037	ARHGAP29	Rho GTPase activating protein 29	Multiple_Complex
TC0300012051.hg.1	7.11	9.64	-5.77	5.55E-06	0.0081	nekeme	Transcript Identified by AceView	Unassigned
TC1100007841.hg.1	3.77	6.33	-5.91	0.0006	0.0759	SCGB2A2	secretoglobin, family 2A, member 2	Coding
TC0700008108.hg.1	5.64	8.2	-5.92	0.0005	0.0679	teymorby	Transcript Identified by AceView	Coding
TC0600011173.hg.1	10.94	13.52	-5.96	0.0012	0.0964	GUSBP2	glucuronidase, beta pseudogene 2	Multiple_Complex
TC0200010378.hg.1	4.96	7.55	-6.03	4.94E-06	0.0078	meyveybo	Transcript Identified by AceView	Coding
TC0500013317.hg.1	9.46	12.05	-6.05	0.0003	0.0603	SMA4; LOC100170939	glucuronidase, beta pseudogene; Homo sapiens glucuronidase, beta pseudogene (SMA4), transcript variant 1, non-coding	Multiple_Complex

							RNA.; Jeck2013, Salzman2013 ANNOTATED, INTERNAL, ncRNA, OVEXON best transcript NR_029426; Salzman2013 ALT_ACCEPTOR, INTERNAL, intronic, ncRNA, OVERLAPTX, OVEXON best transcript NR_024054; Salzman2013 ALT_ACCEPTOR, ALT_DONOR, INTERNAL, intronic, ncRNA, OVEXON best transcript NR_029426; Salzman2013 ALT_DONOR, INTERNAL, ncRNA, OVEXON best transcript NR_029426; Salzman2013 ALT_ACCEPTOR, INTERNAL, intronic, ncRNA, OVEXON best transcript NR_029426; Salzman2013 ANNOTATED, INTERNAL, ncRNA, OVEXON best transcript NR_029426; Salzman2013 ALT_ACCEPTOR, ALT_DONOR, INTERNAL, intronic, ncRNA best transcript NR_029426; Salzman2013 ALT_DONOR, INTERNAL, intronic, ncRNA, OVEXON best transcript NR_029426	
TC1000010589.hg.1	4.99	7.59	-6.05	0.0003	0.0564	VSTM4	V-set and transmembrane domain containing 4	Multiple_Complex
TC0300012068.hg.1	4.63	7.23	-6.06	0.001	0.0911	sweybobu	Transcript Identified by AceView	Coding
TC0100013231.hg.1	4.23	6.84	-6.1	0.0002	0.0486			NonCoding
TC0100013182.hg.1	7.41	10.03	-6.12	0.0004	0.0624	CAMK2N1	calcium/calmodulin-dependent protein kinase II inhibitor 1	Multiple_Complex
TC1300009585.hg.1	4.03	6.65	-6.13	2.21E-05	0.0167	hikoru	Transcript Identified by AceView	Unassigned
TC0200011109.hg.1	3.79	6.41	-6.17	0.0005	0.0679			NonCoding
TC1900010688.hg.1	6.4	9.03	-6.19	6.05E-05	0.028	kloyjer	Transcript Identified by AceView	Coding
TC0600011370.hg.1	5.36	7.99	-6.2	5.75E-06	0.0083			NonCoding
TC0900010089.hg.1	7.82	10.47	-6.29	0.0008	0.0834	butoybu; slavarbu	Transcript Identified by AceView	Coding
TC0700011628.hg.1	4.63	7.28	-6.3	1.32E-05	0.0127	spako	Transcript Identified by AceView	Coding
TC1200010436.hg.1	11.98	14.64	-6.31	7.33E-05	0.0307	shojerby	Transcript Identified by AceView	Unassigned
TC0900008055.hg.1	6.1	8.76	-6.32	0.001	0.0888	chershee	Transcript Identified by AceView	Unassigned

TC1700010973.hg.1	5.03	7.69	-6.32	0.0002	0.0452	COPZ2	coatomer protein complex subunit zeta 2	Multiple_Complex
TC0900008053.hg.1	7.98	10.65	-6.36	0.0008	0.0843	gawlar	Transcript Identified by AceView	Coding
TC2100006497.hg.1	3.51	6.18	-6.37	0.0007	0.0769	RP11-782C8.5		Multiple_Complex
TC1700010676.hg.1	5.3	7.98	-6.4	0.0005	0.0697	KRT15; MIR6510	keratin 15, type I; microRNA 6510	Multiple_Complex
TC0Y00007330.hg.1	3.93	6.61	-6.41	6.59E-05	0.0289	AC006328.4	Uncharacterized protein [Source:UniProtKB/TrEMBL;Acc:A0A096LPA0]	Multiple_Complex
TC0200009761.hg.1	6.29	8.99	-6.49	0.0003	0.0559	loydo	Transcript Identified by AceView	Unassigned
TC1300008774.hg.1	4.89	7.58	-6.5	4.17E-05	0.0226	RP11-5G9.5	novel transcript, sense intronic to CCDC122	NonCoding
TC1500010251.hg.1	5.55	8.25	-6.5	6.26E-07	0.0026	HOMER2	homer scaffolding protein 2	Multiple_Complex
TC0700008358.hg.1	6.1	8.81	-6.53	0.0001	0.0369	COL1A2	collagen, type I, alpha 2	Multiple_Complex
TC0800009460.hg.1	4.85	7.56	-6.54	1.16E-06	0.0037	LOC100289400	Transcript Identified by AceView, Entrez Gene ID(s) 100289400	Unassigned
TC1600009958.hg.1	5.56	8.28	-6.57	0.0002	0.0498	NPIPB4	nuclear pore complex interacting protein family, member B4	Multiple_Complex
TC1500006675.hg.1	8.3	11.02	-6.58	4.06E-06	0.0068	HERC2P9	hect domain and RLD 2 pseudogene 9	Multiple_Complex
TC1500009658.hg.1	5.07	7.79	-6.59	2.42E-05	0.0171	gukley; zerstar	Transcript Identified by AceView	NonCoding
TC1200007592.hg.1	4.27	6.99	-6.62	0.0007	0.0801	AQP5	aquaporin 5	Multiple_Complex
TC17_GL000205v2_random00006436.hg.1	10.68	13.41	-6.65	0.0005	0.0699	AC011841.2		Precursor_microRNA
TC0300011409.hg.1	3.82	6.56	-6.67	2.54E-07	0.0016			NonCoding
TC0200007908.hg.1	7.77	10.51	-6.7	5.40E-06	0.0081	ANTXR1	anthrax toxin receptor 1	Multiple_Complex
TC0500008570.hg.1	8.37	11.12	-6.73	0.0003	0.0533	sweeswarbu	Transcript Identified by AceView	Coding
TCUn_GL000219v100006434.hg.1	8	10.76	-6.75	0.0002	0.0487	AL592183.5		Precursor_microRNA
TC0900007752.hg.1	5.8	8.56	-6.8	0.0003	0.0598	NTRK2	neurotrophic tyrosine kinase, receptor, type 2	Multiple_Complex
TC1600008235.hg.1	6.78	9.56	-6.85	0.0002	0.0438	RP11-140H17.2	novel transcript sense intronic to NFAT5	NonCoding
TC1100007840.hg.1	4.68	7.45	-6.86	7.64E-07	0.003	SCGB1D2	secretoglobin, family 1D, member 2	Coding
TC1600006671.hg.1	9.77	12.56	-6.93	0.001	0.0932			NonCoding
TSUnmapped00000842.hg.1	9.77	12.56	-6.93	0.001	0.0932			NonCoding
TC0400009223.hg.1	3.63	6.43	-7.01	7.21E-05	0.0306	CPE	carboxypeptidase E	Coding
TC14_KI270722v1_random00006432.hg.1	9.63	12.44	-7.01	0.0007	0.0785	AC138774.3		Precursor_microRNA
TC0100010000.hg.1	4.55	7.37	-7.04	0.0005	0.0679	LCE2B	late cornified envelope 2B	Coding
TC1500007510.hg.1	6.38	9.22	-7.13	1.22E-06	0.0038	TPM1	tropomyosin 1 (alpha)	Multiple_Complex
TC1800008209.hg.1	10.07	12.91	-7.14	0.0004	0.0667	karbla	Transcript Identified by AceView	Coding
TC0700008181.hg.1	9.07	11.91	-7.16	0.0005	0.0683	GNAI1	guanine nucleotide binding protein (G protein), alpha inhibiting activity polypeptide 1	Multiple_Complex
TC1600009179.hg.1	9.09	11.96	-7.34	0.0003	0.0564			NonCoding

TC1400008945.hg.1	8.05	10.93	-7.36	3.84E-05	0.0216	RALGAPA1	Ral GTPase activating protein, alpha subunit 1 (catalytic)	Multiple_Complex
TC2200007623.hg.1	6.35	9.24	-7.41	0.0002	0.0504	blashee	Transcript Identified by AceView	Coding
TC1000007363.hg.1	3.44	6.33	-7.41	0.0011	0.0935			NonCoding
TC0X00011276.hg.1	8.67	11.57	-7.44	4.42E-07	0.002	CA5BP1	carbonic anhydrase VB pseudogene 1	Multiple_Complex
TC0100017142.hg.1	4.89	7.8	-7.47	8.52E-07	0.0031	CD34	CD34 molecule	Multiple_Complex
TC0400006628.hg.1	6.67	9.6	-7.61	4.13E-05	0.0226			NonCoding
TC1700007216.hg.1	4.11	7.04	-7.61	0.0008	0.0838	CCDC144CP	coiled-coil domain containing 144C, pseudogene	Multiple_Complex
TC1400010684.hg.1	5.17	8.1	-7.62	0.0009	0.0864	SNORD114-25	small nucleolar RNA, C/D box 114-25	Multiple_Complex
TC1000009025.hg.1	4.91	7.89	-7.88	2.49E-06	0.0054	EMX2	empty spiracles homeobox 2	Multiple_Complex
TC1000007199.hg.1	7.36	10.34	-7.88	6.83E-06	0.0092	ZEB1	zinc finger E-box binding homeobox 1	Multiple_Complex
TC1900010097.hg.1	4.88	7.86	-7.92	0.0007	0.0774			NonCoding
TC0800010807.hg.1	8.18	11.17	-7.96	0.0001	0.0409	RPL7	ribosomal protein L7	Multiple_Complex
TC1400008585.hg.1	7.8	10.81	-8.02	2.17E-08	0.0003	SNORA79; AL355075.1	Small nucleolar RNA SNORA79 [Source:RFAM;Acc:RF00600]	Multiple_Complex
TC1000011740.hg.1	5.86	8.87	-8.05	2.44E-05	0.0171	COL17A1; MIR936	collagen, type XVII, alpha 1; microRNA 936	Multiple_Complex
TC0200014092.hg.1	5.77	8.78	-8.07	6.13E-06	0.0085	vawswawby	Transcript Identified by AceView	Coding
TC0100011631.hg.1	5.05	8.06	-8.07	1.71E-06	0.0044	lyber	Transcript Identified by AceView	Unassigned
TC1100009296.hg.1	4.38	7.41	-8.14	0.0006	0.0727	rorkarby	Transcript Identified by AceView	Unassigned
TC1500008840.hg.1	5.48	8.51	-8.16	8.95E-05	0.0333	TJP1	tight junction protein 1	Multiple_Complex
TC1200010768.hg.1	4.34	7.37	-8.16	0.0001	0.0359	KRT1	keratin 1, type II	Multiple_Complex
TC0300007878.hg.1	5.28	8.31	-8.18	6.53E-05	0.0289	AC133041.1		Precursor_microRNA
TC1200009954.hg.1	10.57	13.62	-8.27	0.0012	0.0987			NonCoding
TC0500012760.hg.1	6.71	9.77	-8.33	0.0007	0.0785	SLIT3	slit guidance ligand 3	Multiple_Complex
TC0600012240.hg.1	4.79	7.87	-8.46	1.61E-05	0.0144	LMBRD1	LMBR1 domain containing 1	Multiple_Complex
TC1800008387.hg.1	6.07	9.17	-8.54	0.0002	0.0497	DSC1	desmocollin 1	Coding
TC0200016553.hg.1	3.64	6.74	-8.56	0.0012	0.0975	DAPL1; OR7E89P	death associated protein like 1; olfactory receptor, family 7, subfamily E, member 89 pseudogene	Multiple_Complex
TC0200010630.hg.1	10.23	13.34	-8.63	0.001	0.0926	gyswyby	Transcript Identified by AceView	Coding
TC0300011417.hg.1	10.06	13.19	-8.75	0.0003	0.0555	Y_RNA	Y RNA [Source:RFAM;Acc:RF00019]	NonCoding
TC0400009110.hg.1	4.63	7.76	-8.76	0.0002	0.0477	GRIA2	glutamate receptor, ionotropic, AMPA 2	Multiple_Complex
TC2200007152.hg.1	3.16	6.29	-8.76	3.21E-05	0.0193	plyvaw	Transcript Identified by AceView	Unassigned
TC0600011202.hg.1	14.82	17.99	-9.02	0.0011	0.0933			NonCoding
TC0900009570.hg.1	4.93	8.11	-9.09	4.38E-06	0.0072	rike	Transcript Identified by AceView	Unassigned
TC0900007392.hg.1	13.84	17.05	-9.24	0.0006	0.0736			NonCoding

TC0700006847.hg.1	3.84	7.05	-9.25	0.0005	0.0679	SNORD56	Small nucleolar RNA SNORD56 [Source:RFAM;Acc:RF 00275]	Small_RNA
TC0800007532.hg.1	8.81	12.06	-9.46	3.50E-05	0.0204	kenimu	Transcript Identified by AceView	Coding
TC0800007185.hg.1	7.62	10.87	-9.47	0.0001	0.0415	RBPMS	RNA binding protein with multiple splicing	Multiple_Co mplex
TC0100014178.hg.1	10.02	13.27	-9.49	0.0001	0.0396	fermerbu	Transcript Identified by AceView	Coding
TC0400011263.hg.1	8.63	11.9	-9.69	0.0002	0.0436	SPARCL1	SPARC like 1	Multiple_Co mplex
TC1200010766.hg.1	4.28	7.55	-9.7	0.0007	0.0798	KRT2	keratin 2, type II	Multiple_Co mplex
TC0800008481.hg.1	4.77	8.07	-9.79	0.0001	0.0396	CTHRC1	collagen triple helix repeat containing 1	Multiple_Co mplex
TC0400007998.hg.1	7.81	11.11	-9.87	1.58E-06	0.0043			NonCoding
TC0100009183.hg.1	6.48	9.8	-10.02	2.95E-05	0.0186	PALMD	palmdelphin	Multiple_Co mplex
TC0700011626.hg.1	6.32	9.65	-10.09	0.0003	0.0566	SEMA3C	sema domain, immunoglobulin domain (Ig), short basic domain, secreted, (semaphorin) 3C	Multiple_Co mplex
TC1000010384.hg.1	4.95	8.31	-10.33	0.0006	0.076			NonCoding
TC2000006799.hg.1	8.48	11.89	-10.62	2.45E-05	0.0171	DSTN	destrin (actin depolymerizing factor)	Multiple_Co mplex
TC0900009571.hg.1	7.54	10.96	-10.65	2.09E-05	0.0164	temire	Transcript Identified by AceView	Unassigned
TC0800008324.hg.1	6.03	9.45	-10.75	4.17E-08	0.0004	MATN2	matrilin 2	Multiple_Co mplex
TC1500007511.hg.1	5.93	9.35	-10.76	3.89E-08	0.0004	garstaw	Transcript Identified by AceView	Unassigned
TC0100011219.hg.1	3.51	6.97	-10.97	1.55E-05	0.0141	PPP1R12B	protein phosphatase 1, regulatory subunit 12B	Multiple_Co mplex
TC0400007468.hg.1	3.92	7.41	-11.24	0.0004	0.0652			NonCoding
TC0100008520.hg.1	6.31	9.83	-11.46	0.0004	0.0661	luzawbo	Transcript Identified by AceView	Unassigned
TC1000011494.hg.1	4.02	7.56	-11.57	5.32E-06	0.0081	veemy	Transcript Identified by AceView	Unassigned
TC0300008357.hg.1	4.96	8.49	-11.59	1.25E-05	0.0124	klerbubu	Transcript Identified by AceView	Unassigned
TC0100018058.hg.1	3.37	6.98	-12.24	0.001	0.0891	lorleebo	Transcript Identified by AceView	Unassigned
TC1000011370.hg.1	7	10.63	-12.39	1.13E-05	0.0119	ACTA2	actin, alpha 2, smooth muscle, aorta	Multiple_Co mplex
TC0200014754.hg.1	6.81	10.44	-12.41	0.0008	0.0829			NonCoding
TC0300012212.hg.1	5.69	9.35	-12.65	1.20E-05	0.0124	MYLK	myosin light chain kinase	Multiple_Co mplex
TC0200007399.hg.1	7	10.68	-12.79	3.80E-07	0.002	PLEKHH2	pleckstrin homology domain containing, family H (with MyTH4 domain) member 2	Multiple_Co mplex
TC1000007201.hg.1	6.82	10.53	-13.15	7.74E-05	0.0311	moysler	Transcript Identified by AceView	Coding
TC1200010111.hg.1	4.79	8.52	-13.21	1.41E-05	0.013	ABCC9	ATP binding cassette subfamily C member 9	Coding
TC0600011171.hg.1	6.35	10.09	-13.37	0.0006	0.0727	bysnoybu	Transcript Identified by AceView	Unassigned
TC0400010678.hg.1	4.05	7.79	-13.37	0.0001	0.0395			NonCoding
TC0400012977.hg.1	7.75	11.52	-13.65	3.34E-08	0.0004	SH3D19	SH3 domain containing 19	Multiple_Co mplex
TC0200007847.hg.1	13.03	16.81	-13.7	0.0007	0.0796			NonCoding
TC0100010887.hg.1	8.26	12.05	-13.86	1.50E-06	0.0043	C1orf21	chromosome 1 open reading frame 21	Multiple_Co mplex
TC2200008123.hg.1	10.13	13.95	-14.15	0.0013	0.0999			NonCoding

TC0X00010082.hg.1	4.91	8.8	-14.8	7.42E-05	0.0307	shargoy	Transcript Identified by AceView	Unassigned
TC1800007411.hg.1	4.4	8.32	-15.06	6.66E-05	0.0291	NEDD4L	neural precursor cell expressed, developmentally down-regulated 4-like, E3 ubiquitin protein ligase	Multiple_Complex
TC1300008018.hg.1	4.04	7.99	-15.52	0.0001	0.0404	RP11-291I6.2	novel transcript sense intronic to COL4A2	NonCoding
TC1900010432.hg.1	3.83	7.8	-15.6	0.0003	0.0572	SCGB1B2P	secretoglobin, family 1B, member 2, pseudogene	NonCoding
TC1800007523.hg.1	4.98	9.08	-17.08	2.89E-06	0.0057	PHLPP1	PH domain and leucine rich repeat protein phosphatase 1	Multiple_Complex
TC2000009242.hg.1	5.9	10.01	-17.3	5.37E-05	0.0262	WFDC3	WAP four-disulfide core domain 3	Multiple_Complex
TC0100008177.hg.1	5.29	9.53	-18.82	6.29E-05	0.0283	ramawbu	Transcript Identified by AceView	Coding
TC0200008370.hg.1	3.19	7.74	-23.5	0.0004	0.0619			NonCoding
TC0400012173.hg.1	7.76	12.39	-24.78	3.76E-06	0.0065	SFRP2	secreted frizzled-related protein 2	Coding
TC0600006869.hg.1	7.88	12.6	-26.37	6.98E-06	0.0092	DSP	desmoplakin	Multiple_Complex
TC0400010767.hg.1	10.39	15.13	-26.69	9.30E-05	0.0341			NonCoding
TC0600012123.hg.1	8.18	12.94	-27.13	2.55E-06	0.0054	DST	dystonin	Multiple_Complex
TC0700009481.hg.1	6.04	10.86	-28.36	0.0002	0.0506	PIP	prolactin-induced protein	Coding
TC0500008868.hg.1	7.09	11.94	-28.98	0.0008	0.0838	VTRNA1-2	vault RNA 1-2	Multiple_Complex
TC0200010239.hg.1	5.61	10.51	-29.89	6.26E-05	0.0283	COL3A1; MIR3606	collagen, type III, alpha 1; microRNA 3606	Multiple_Complex
TC1600011505.hg.1	3.72	8.68	-31.17	0.0001	0.0371	NPIPB4	nuclear pore complex interacting protein family, member B4	Multiple_Complex
TC0300014031.hg.1	7.09	12.08	-31.77	2.12E-05	0.0164	CCDC80; LINC01279	coiled-coil domain containing 80; long intergenic non-protein coding RNA 1279	Multiple_Complex
TC0Y00006481.hg.1	9.33	14.39	-33.26	0.0006	0.0755	XGY2	Homo sapiens Xg pseudogene, Y-linked 2 (XGY2), non-coding RNA.	NonCoding
TC1200011496.hg.1	11.78	17.05	-38.72	1.93E-05	0.0156	DCN	decorin	Multiple_Complex
TC0200013412.hg.1	5.62	11.09	-44.16	6.57E-05	0.0289			NonCoding
TC1200010846.hg.1	3.93	9.6	-51.12	5.97E-05	0.0279	DCD	dermcidin	Coding
TC0100007367.hg.1	3.25	9.63	-83.21	2.07E-06	0.0048			NonCoding
TC1900010479.hg.1	7.02	13.79	-109.22	2.51E-05	0.0174	DMKN	dermokine	Multiple_Complex
TC1700010639.hg.1	4.75	12.08	-161.35	5.39E-05	0.0262	KRT10	keratin 10, type I	Multiple_Complex
TC0500008568.hg.1	4.39	12.11	-211.24	1.98E-05	0.0159	SLC12A2	solute carrier family 12 (sodium/potassium/chloride transporter), member 2	Multiple_Complex
TC1300007752.hg.1	3.78	11.69	-239.71	4.17E-05	0.0226	CLDN10	claudin 10	Multiple_Complex
TC0100015830.hg.1	4.81	13.13	-319.75	5.50E-06	0.0081	FLG	filaggrin	Multiple_Complex
TC0400007470.hg.1	4.1	12.87	-434.27	3.63E-07	0.002			NonCoding

Supplemental Table 2: Neutrophil-specific Mutation in Refractory Sweet Syndrome patient.

Function	Gene	Gene Description	Fold change	P-value	FDR P-Val	Exonic|Biotype	Transcript	LOF|NMD	Chr	Start	End	Ref	Obs
protein_protein_contact	PABPC1	poly(A) binding protein, cytoplasmic 1; microRNA 7705 (MIR7705)	-1.06	0.8176	0.8823	protein_coding	:c.1735G>A		chr8	101717237	101717237	C	T
splice_acceptor_variant+intron_variant	KCTD21-AS1	KCTD21 antisense RNA 1	1.3	0.0625	0.1503		NR_102280.1:n.389-1G>A		chr11	77860022	77860022	G	A
splice_acceptor_variant+intron_variant	MMEL1	membrane metallo-endopeptidase-like 1	1.07	0.5492	0.6769	protein_coding	NM_033467.3:c.536-1G>A	MMEL1:1.00(1);	chr1	2538509	2538509	C	T
splice_acceptor_variant+intron_variant	CPNE7	copine VII	1.07	0.6113	0.7279	protein_coding	NM_014427.4:c.658-1G>A	CPNE7:1.00(2);	chr16	89650435	89650435	G	A
splice_acceptor_variant+intron_variant	RAB41	RAB41, member RAS oncogene family	-1.14	0.2275	0.3683	protein_coding	NM_001032726.2:c.341-1G>A	RAB41:1.00(1);	chrX	69503362	69503362	G	A
splice_acceptor_variant+intron_variant	NUDCD3	NudC domain containing 3	-1.73	0.0008	0.0086	protein_coding	NM_015332.3:c.193-1G>A	NUDCD3:1.00(1);	chr7	44524884	44524884	C	T
splice_acceptor_variant+intron_variant	PIK3C3	phosphatidylinositol 3-kinase, catalytic subunit type 3	-2.48	0.0445	0.119	protein_coding	NM_002647.3:c.2650-2A>G	PIK3C3:1.00(2);	chr18	39661085	39661085	A	G
splice_donor_variant+intron_variant	NOM1	nucleolar protein with MIF4G domain 1	1.42	0.0001	0.0027	protein_coding	NM_138400.1:c.1911+1G>A	NOM1:1.00(1);	chr7	156755870	156755870	G	A
splice_donor_variant+intron_variant	CADM2	cell adhesion molecule 2	1.36	0.0322	0.0955	protein_coding	NM_153184.3:c.770+1G>T	CADM2:1.00(7);	chr3	85985008	85985008	G	T
splice_donor_variant+intron_variant	PYCR1	pyrroline-5-carboxylate reductase 1	1.28	0.0073	0.0356	protein_coding	NM_001282281.1:c.878+1G>A	PYCR1:1.00(5);	chr17	79892201	79892201	C	T
splice_donor_variant+intron_variant	MIOX	myo-inositol oxygenase	1.12	0.0553	0.138	protein_coding	NM_017584.5:c.340+1G>T	MIOX:1.00(1);	chr22	50926478	50926478	G	T
splice_donor_variant+intron_variant	KDM2B	lysine (K)-specific demethylase 2B	1.09	0.9253	0.9546	protein_coding	NM_032590.4:c.931+1G>A	KDM2B:1.00(2);	chr12	121970710	121970710	C	T

splice_donor_variant+intron_variant	TIMD4	T-cell immunoglobulin and mucin domain containing 4	-1.05	0.5782	0.7012	protein_coding	NM_138379.2:c.760+1G>A	TIMD4:1.00(2);	chr5	156376661	156376661	C	T
splice_donor_variant+intron_variant	FRG1BP	FSHD region gene 1 family member B, pseudogene	-1.53	0.0178	0.0643		NR_003579.1:n.290+1G>A		chr20	29614328	29614328	G	A
splice_donor_variant+intron_variant	CHD6	chromodomain helicase DNA binding protein 6	-1.61	0.0071	0.0349	protein_coding	NM_032221.4:c.4007+1G>A	CHD6:1.00(1);	chr20	40068639	40068639	C	T
stop_gained	CDAN1	codanin 1	1.68	0.0002	0.0041	NONSENSE	NM_138477.2:p.Gln1059*/c.3175C>T	CDAN1:1.00(1); CDAN1:1.00(1);	chr15	43018537	43018537	G	A
stop_gained	ABCC12	ATP binding cassette subfamily C member 12	1.47	0.0012	0.0116	NONSENSE	NM_033226.2:p.Arg94*/c.280C>T	ABCC12:1.00(1);ABCC12:1.00(1);	chr16	48175260	48175260	G	A
stop_gained	MYO9A	Memczak2013 ANTISENSE, CDS, coding, INTERNAL best transcript NM_006901	1.38	0.0341	0.0994	NONSENSE	NM_006901.3:p.Trp2021*/c.6063G>A	MYO9A:1.00(1); MYO9A:1.00(1);	chr15	72154869	72154869	C	T
stop_gained	FAM120C	family with sequence similarity 120C	1.33	0.0002	0.0039	NONSENSE	NM_001300788.1:p.Trp885*/c.2655G>A		chrX	54099690	54099690	C	T
stop_gained	DLGAP3	discs, large (Drosophila) homolog-associated protein 3	1.32	0.0017	0.0145	NONSENSE	NM_001080418.2:p.Ser305*/c.914C>A	DLGAP3:1.00(1); DLGAP3:1.00(1);	chr1	35370071	35370071	G	T
stop_gained	NRXN1	neurexin 1	1.19	0.1894	0.3239	NONSENSE	NM_001135659.2:p.Gln57*/c.169C>T	NRXN1:0.76(25);NRXN1:0.76(25);	chr2	51255243	51255243	G	A
stop_gained	MYO18A	TGFB1-induced anti-apoptotic factor 1; myosin XVIIIA	1.15	0.1144	0.2283	NONSENSE	NM_078471.3:p.Gln696*/c.2086C>T	MYO18A:1.00(2);MYO18A:1.00(2);	chr17	27442823	27442823	G	A
stop_gained	HEATR1	HEAT repeat containing 1	1.13	0.7791	0.855	NONSENSE	NM_018072.5:p.Glu1164*/c.3490G>T	HEATR1:1.00(1); HEAT	chr1	236736098	236736098	C	A

								R1:1. 00(1);					
stop_gain ed	C15orf 53	chromosome 15 open reading frame 53	1.13	0.19 49	0.33 05	NONSE NSE	NM_207444. 2:p.Trp67*/c. 200G>A		chr15	389904 06	389904 06	G	A
stop_gain ed	ZNF39 1	zinc finger protein 391	1.11	0.07 89	0.17 66	NONSE NSE	NM_001076 781.2:p.Gly8 2*/c.244G>T		chr6	273683 93	273683 93	G	T
stop_gain ed	C6orf5 8	chromosome 6 open reading frame 58	1.11	0.49 35	0.62 85	NONSE NSE	NM_001010 905.2:p.Trp3 3*/c.99G>A	C6orf 58:1.0 0(1);C 6orf5 8:1.00 (1);	chr6	127898 429	127898 429	G	A
stop_gain ed	LOC1 00129 697	uncharacterized LOC100129697 ; novel transcript, antisense to CBFA2T3	1.1	0.52 93	0.65 99	NONSE NSE	NM_001290 330.1:p.Trp3 10*/c.929G> A		chr16	890174 55	890174 55	G	A
stop_gain ed	LOC1 00129 697	uncharacterized LOC100129697 ; novel transcript, antisense to CBFA2T3	1.1	0.52 93	0.65 99	NONSE NSE	NM_001290 330.1:p.Trp3 10*/c.930G> A		chr16	890174 56	890174 56	G	A
stop_gain ed	TSGA 13	coatomer protein complex subunit gamma 2 (COPG2); testis specific 13	1.05	0.32 37	0.47 03	NONSE NSE	NM_001304 968.1:p.Tyr2 03*/c.609C> G		chr7	130356 550	130356 550	G	C
stop_gain ed	DNAJ C12	DnaJ (Hsp40) homolog, subfamily C, member 12	1.05	0.35 66	0.50 29	NONSE NSE	NM_021800. 2:p.Trp90*/c. 270G>A	DNAJ C12:0 .50(2) ;DNA JC12: 0.50(2);	chr10	695713 09	695713 09	C	T
stop_gain ed	VWA8	von Willebrand factor A domain containing 8; microRNA 5006	1.05	0.85 58	0.90 84	NONSE NSE	NM_015058. 1:p.Gln1872 */c.5614C>T		chr13	421424 37	421424 37	G	A
stop_gain ed	LRP1 B	LDL receptor related protein 1B	1.04	0.90 53	0.94 14	NONSE NSE	NM_018557. 2:p.Trp2285 */c.6854G>A	LRP1 B:1.0 0(1);L RP1B :1.00(1);	chr2	141359 154	141359 154	C	T
stop_gain ed	CEP6 8	centrosomal protein 68kDa	1.02	0.99 34	0.99 59	NONSE NSE	NM_001319 100.1:p.Ser5 32*/c.1595C >A	CEP6 8:0.50 (4);C EP68: 0.50(4);	chr2	652998 25	652998 25	C	A
stop_gain ed	OVGP 1	oviductal glycoprotein 1	-1.01	0.38 75	0.53 27	NONSE NSE	NM_002557. 3:p.Trp31*/c. 93G>A	OVG P1:1. 00(1);	chr1	111969 226	111969 226	C	T

								OVGP1:1.00(1);					
stop_gained	OVGP1	oviductal glycoprotein 1	-1.01	0.3875	0.5327	NONSENSE	NM_002557.3:p.Trp31*/c.92G>A	OVGP1:1.00(1);OVGP1:1.00(1);	chr1	111969227	111969227	C	T
stop_gained	FREM3	FRAS1 related extracellular matrix 3	-1.02	0.9767	0.9861	NONSENSE	NM_001168235.1:p.Glu1438*/c.4312G>T	FREM3:1.00(1);FREM3:1.00(1);	chr4	144617517	144617517	C	A
stop_gained	TNRC6C	trinucleotide repeat containing 6C	-1.02	0.9221	0.9526	NONSENSE	NM_001142640.1:p.Trp898*/c.2693G>A	TNRC6C:1.00(2);TNRC6C:1.00(2);	chr17	76063928	76063928	G	A
stop_gained	TNRC6C	trinucleotide repeat containing 6C	-1.02	0.9221	0.9526	NONSENSE	NM_001142640.1:p.Trp898*/c.2694G>A	TNRC6C:1.00(2);TNRC6C:1.00(2);	chr17	76063929	76063929	G	A
stop_gained	SLC37A1	solute carrier family 37 (glucose-6-phosphate transporter), member 1	-1.06	0.5129	0.6457	NONSENSE	NM_001320537.1:p.Trp214*/c.642G>A	SLC37A1:1.00(2);SLC37A1:1.00(2);	chr21	43963624	43963624	G	A
stop_gained	IFFO2	intermediate filament family orphan 2	-1.08	0.7153	0.8083	NONSENSE	NM_001136265.1:p.Glu430*/c.1288G>T	IFFO2:1.00(1);IFFO2:1.00(1);	chr1	19237907	19237907	C	A
stop_gained	CHN2	chimerin 2	-1.12	0.6131	0.7294	NONSENSE	NM_001293069.1:p.Arg273*/c.817C>T	CHN2:0.69(16);CHN2:0.69(16);	chr7	29519910	29519910	C	T
stop_gained	PLEKHA3	Transcript Identified by AceView, Entrez Gene ID(s) 65977	-1.22	0.0423	0.1149	NONSENSE	NM_019091.3:p.Lys265*/c.793A>T		chr2	179368504	179368504	A	T
stop_gained	NDE1	nudE neurodevelopment protein 1; microRNA 484	-1.22	0.2827	0.4279	NONSENSE	NM_001143979.1:p.Trp18*/c.54G>A	NDE1:1.00(2);NDE1:1.00(2);	chr16	15758689	15758689	G	A
stop_gained	MGAM2	maltase-glucoamylase 2 (putative)	-1.26	0.0582	0.1429	NONSENSE	NM_001293626.1:p.Trp88*/c.263G>A	MGAM2:1.00(1);MGA	chr7	141830824	141830824	G	A

								M2:1.00(1);					
stop_gained	KAT6A	K(lysine) acetyltransferase 6A	-1.32	0.0675	0.1585	NONSENSE	NM_006766.4:p.Arg1019*/c.3055C>T	KAT6A:0.50(2);KAT6A:0.50(2);	chr8	41795071	41795071	G	A
stop_gained	DDHD2	DDHD domain containing 2	-1.6	0.0268	0.0843	NONSENSE	NM_001164232.1:p.Arg476*/c.1426A>T	DDHD2:0.67(3);DDHD2:0.67(3);	chr8	38109511	38109511	A	T
stop_gained	NUDCD3	NudC domain containing 3	-1.73	0.0008	0.0086	NONSENSE	NM_015332.3:p.Gln176*/c.526C>T	NUDCD3:1.00(1);NUDCD3:1.00(1);	chr7	44467286	44467286	G	A
stop_gained	GLI2	Jeck2013 ALT_ACCEPTOR, ALT_DONOR, coding, INTERNAL, intronic best transcript NM_005270	-1.76	0.0048	0.0273	NONSENSE	NM_005270.4:p.Gln318*/c.952C>T	GLI2:1.00(1);GLI2:1.00(1);	chr2	121728075	121728075	C	T
stop_gained	AGL	amylo-alpha-1, 6-glucosidase, 4-alpha-glucanotransferase	-1.87	0.0196	0.0686	NONSENSE	NM_000028.2:p.Ser978*/c.2933C>A	AGL:1.00(6);AGL:1.00(6);	chr1	100356896	100356896	C	A
stop_gained	ZNF141	zinc finger protein 141	-1.95	3.40E-06	0.0004	NONSENSE	NM_003441.2:p.Gly363*/c.1087G>T		chr4	367313	367313	G	T
stop_gained	ZNF705A	zinc finger protein 705A; family with sequence similarity 66, member C	-2.48	1.63E-06	0.0002	NONSENSE	NM_001004328.2:p.Cys184*/c.552C>A		chr12	8329828	8329828	C	A
stop_gained	COL14A1	collagen, type XIV, alpha 1	-2.61	0.0289	0.0887	NONSENSE	NM_021110.3:p.Glu1366*/c.4096G>T	COL14A1:1.00(1);COL14A1:1.00(1);	chr8	121301865	121301865	G	T
stop_gained	FAF1	Fas (TNFRSF6) associated factor 1	-2.63	0.0065	0.0333	NONSENSE	NM_007051.2:p.Gln134*/c.400C>T	FAF1:1.00(1);FAF1:1.00(1);	chr1	51210415	51210415	G	A
stop_gained	CFH	complement factor H	-4.86	6.79E-07	0.0001	NONSENSE	NM_000186.3:p.Trp1037*/c.3111G>A	CFH:0.50(2);CF	chr1	196711159	196711159	G	A

								H:0.50(2);					
stop_gained+splice_region_variant	SNX10	sorting nexin 10	1.15	0.2853	0.4307	NONSENSE	NM_001318198.1:p.Ser202*/c.605C>A		chr7	26412113	26412113	C	A
structural_interaction_variant	LY96	lymphocyte antigen 96	1.63	0.1664	0.2963	protein_coding	:c.206G>A		chr8	74922239	74922239	G	A
structural_interaction_variant	FEN1	flap structure-specific endonuclease 1	1.51	0.0003	0.0052	protein_coding	:c.514G>A		chr11	61563347	61563347	G	A
structural_interaction_variant	DOCK2	Memczak2013 ANTISENSE, CDS, coding, INTERNAL best transcript NM_004946	1.46	0.0014	0.0126	protein_coding	:c.4208G>A		chr5	169477396	169477396	G	A
structural_interaction_variant	MGAM	maltase-glucoamylase	1.14	0.0421	0.1145	protein_coding	:c.4311G>A		chr7	141763352	141763352	G	A
structural_interaction_variant	SEC14L2	SEC14-like lipid binding 2	1.14	0.0539	0.1356	protein_coding	:c.788A>C		chr22	30811953	30811953	A	C
structural_interaction_variant	POLR2G	polymerase (RNA) II (DNA directed) polypeptide G	1.11	0.2858	0.4311	protein_coding	:c.213G>A		chr11	62530429	62530429	G	A
structural_interaction_variant	CYP2R1	cytochrome P450, family 2, subfamily R, polypeptide 1	1.03	0.7056	0.801	protein_coding	:c.559G>A		chr11	14902123	14902123	C	T
structural_interaction_variant	EPHA3	EPH receptor A3	-1	0.6445	0.7538	protein_coding	:c.362A>G		chr3	89259218	89259218	A	G
structural_interaction_variant	DPP7	dipeptidyl-peptidase 7	-1.14	0.6945	0.7927	protein_coding	:c.1132C>T		chr9	140006400	140006400	G	A
structural_interaction_variant	TLR4	toll-like receptor 4	-1.25	0.435	0.5763	protein_coding	:c.590T>A		chr9	120474996	120474996	T	A
structural_interaction_variant	HMGCS2	3-hydroxy-3-methylglutaryl-CoA synthase 2 (mitochondrial)	-1.4	0.0047	0.027	protein_coding	:c.1517G>A		chr1	120293435	120293435	C	T
structural_interaction_variant	PIK3R1	phosphoinositide-3-kinase, regulatory subunit 1 (alpha)	-6.98	2.74E-06	0.0003	protein_coding	:c.1005G>T		chr5	67588175	67588175	G	T
structural_interaction_variant	RNPC3	RNA binding region (RNP1, RRM) containing 3	-13.25	3.72E-11	5.04E-07	protein_coding	:c.1279T>A		chr1	104088941	104088941	T	A

structural_interaction_variant	CPB2					protein_coding	:c.938C>T		chr13	46632375	46632375	G	A
frameshift_variant	PTGS2	prostaglandin-endoperoxide synthase 2 (prostaglandin G/H synthase and cyclooxygenase)	1.17	0.4956	0.6301	protein_coding	NM_000963.3:p.Asn425fs/c.1272delG	PTGS2:1.00(1);	chr1	186644513	186644513	TC	T
frameshift_variant	MMADHC	methylmalonic aciduria (cobalamin deficiency) cblD type, with homocystinuria	-2.13	0.1104	0.2228	protein_coding	NM_015702.2:p.Ser255fs/c.764delC	MMADHC:1.00(1);	chr2	150426614	150426614	AG	A
frameshift_variant		PMS1 homolog 1, mismatch repair system component	1.19	0.0613	0.1483	protein_coding	NM_001321049.1:p.Lys163fs/c.488delA	PMS1:0.08(13);	chr2	190670539	190670539	TA	T

Supplemental Table 3: Primer sequences.

Gene	Forward Primer	Reverse Primer
GAPDH	GTCTCCTCTGACTTCAACAGCG	ACCACCCTGTTGCTGTAGCCAA
PIK3R1	TGCCTCCTAAACCACCAAAACC	GGTCCCGTCTGCTGTATCTCG
IL1B	CTGAAAGCTCTCCACCTCCA	CCAAGGCCACAGGTATTTTG
IL1R1	ATCGTGATGAATGTGGCTGA	AGGCCTTGTGGGTTTGTTTT
PTK2	TGTGCTCTTGGTTCAAGCTG	CAGCCCTTGTCCGTTAGGTA
SYK	CTCGGGAAGAATCTGAGCAA	CGTAGGAGCCGTTGTTGTCT
VAV1	GGAAGGCGACGAGATCTATG	CAGCAGCGCTTGTCATACTC
Site-Directed Mutagenesis Primer	AATGGTACTGtGGAGATATCTCG	CAGCATCTTGTAAGGACATATTG
Confirmation Mutation Primer	AGATTCTCAGCAGCCAGCTC	AAGCCATATTTCCCATCTCG
